# Molecular Shape, Electronic Factors, and the Ferroelectric Nematic Phase: Investigating the Impact of Structural Modifications

**DOI:** 10.1002/chem.202300073

**Published:** 2023-03-31

**Authors:** Naila Tufaha, Ewan Cruickshank, Damian Pociecha, Ewa Gorecka, John M.D. Storey, Corrie T. Imrie

**Affiliations:** ^1^ Department of Chemistry University of Aberdeen Old Aberdeen AB24 3UE UK; ^2^ Faculty of Chemistry University of Warsaw ul. Zwirki i Wigury 101 02-089 Warsaw Poland

**Keywords:** ferroelectric nematic phase, fluorine, lateral alkoxy chain, liquid crystal, nematic phase

## Abstract

The synthesis and characterisation of two series of low molar mass mesogens, the (4‐nitrophenyl) 2‐alkoxy‐4‐(4‐methoxybenzoyl)oxybenzoates (NT3.*m*) and the (3‐fluoro‐4‐nitrophenyl) 2‐alkoxy‐4‐(4‐methoxybenzoyl)oxybenzoates (NT3F.*m*), are reported in order to investigate the effect of changing the position of a lateral alkoxy chain from the methoxy‐substituted terminal ring to the central phenyl ring in these two series of materials based on RM734. All members of the NT3.*m* series exhibited a conventional nematic phase, N, which preceded the ferroelectric nematic phase, N_F_, whereas all the members of the NT3F.*m* series exhibited direct N_F_‐I transitions except for NT3F.1 which also exhibited an N phase. These materials cannot be described as wedge‐shaped, yet their values of the ferroelectric nematic‐nematic transition temperature, *T* 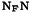 , exceed those of the corresponding materials with the lateral alkoxy chain located on the methoxy‐substituted terminal ring. In part, this may be attributed to the effect that changing the position of the lateral alkoxy chain has on the electronic properties of these materials, specifically on the electron density associated with the methoxy‐substituted terminal aromatic ring. The value of *T*_NI_ decreased with the addition of a fluorine atom *ortho* to the nitro group in NT3F.1, however, the opposite behaviour was found when the transition temperatures of the N_F_ phase were compared which are higher for the NT3F.*m* series. This may reflect a change in the polarity and polarizability of the NT3F.*m* series compared to the NT3.*m* series. Therefore, it is suggested that, rather than simply promoting a tapered shape, the role of the lateral chain in inhibiting anti‐parallel associations and its effect on the electronic properties of the molecules are the key factors in driving the formation of the N_F_ phase.

## Graphical abstract

**Molecular shape and its effects**: The synthesis and characterisation of two series of low molar mass mesogens is reported comparing the effect of having a lateral alkoxy chain in the central phenyl ring versus the chain being attached to the methoxy‐substituted terminal ring. Rather than the molecule being required to show a tapered shape, it is the role of the lateral chain in inhibiting anti‐parallel associations that is key.

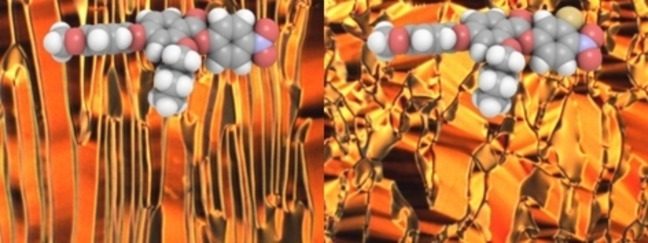

## Introduction

**Figure 1:**
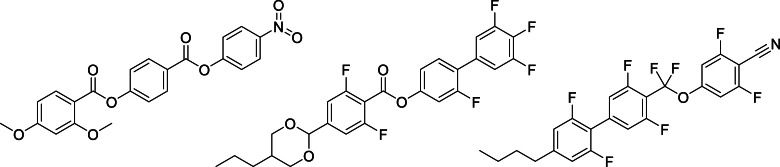
Molecular structures of RM734 (left), DIO (middle) and UUQU‐4‐N (right).

The conventional uniaxial nematic phase, N, although the least ordered liquid crystalline phase, is at the heart of one of the most successful optoelectronic technologies, namely liquid crystal displays.^[1]^ Within the nematic phase, the constituent molecules align along a common direction known as the director, **n**, whereas their centres of mass are randomly arranged. The director has inversion symmetry such that **n**=−**n**, and hence the phase is non‐polar. Over 100 years ago, however, Born suggested that if molecules had a sufficiently large molecular dipole then the interactions between them could drive the formation of the nematic phase, providing those interactions were strong enough to withstand thermal fluctuations and that this phase would be ferroelectric in nature.^[2]^ The first reported experimental observations of a polar ferroelectric nematic phase, N_F_, were reported in 2017 for RM734,[^3^, ^4^] and DIO,^[5]^ Figure 1. In the N_F_ phase there is a spontaneous alignment of the molecular dipoles and the inversion symmetry present in the N phase is lost, i.e. **n**≠−**n**, and the phase is polar.

The overwhelming majority of the materials known to exhibit the N_F_ phase are low molar mass compounds with a large dipole along the long axis of the molecule,[^6^, ^7^, ^8^, ^9^, ^10^, ^11^, ^12^, ^13^, ^14^, ^15^, ^16^, ^17^, ^18^] although a small number of polymers have also been shown to exhibit the N_F_ phase.[^7^, ^19^, ^20^] To date there have been around 200 molecules which have been shown to exhibit the N_F_ phase and these may broadly be categorised into three general structures, based on either RM734,^[3]^ DIO^[5]^ or UUQU‐4‐N,^[8]^ Figure 1. Exceptions to this classification exist such as the highly rigid fluorinated mesogens reported by Song et al.^[21]^ The three archetypal structures of RM734, DIO and UUQU‐4‐N appear somewhat chemically different, but similarities between them are evident and these may be crucial for the rational design of new materials. Specifically, the molecules that show the N_F_ phase possess a large longitudinal molecular dipole, and it has been suggested that this needs to be at least 9 D.^[7]^ In addition, the molecular shape incorporates an element of lateral bulk and this may arise from fluorination of the aromatic rings or from lateral alkoxy chains. These empirical observations are in accord with the computer simulations reported by Berardi et al.,^[22]^ who modelled tapered molecules using a generalised Gay‐Berne type of attractive‐repulsive potential and found that a combination of a non‐centrosymmetric shape with strong attractive forces arising from a large dipole moment showed the ferroelectric nematic phase.

The N_F_ phase has rapidly become the hottest topic in the field of liquid crystals not only because of its fundamental importance but also because of its true application potential. This application potential arises for a number of reasons including that the N_F_ phase is uniaxial and so the polarization is along the director unlike in the more conventional ferroelectric smectic materials;^[23]^ it is easy to align;^[24]^ it has a high non‐linear optical response;[^25^, ^26^] switching occurs for very low electric fields[^5^, ^13^, ^18^, ^27^, ^28^, ^29^] and the phase maintains the fluidity of the nematic phase.^[30]^

**Figure 2:**
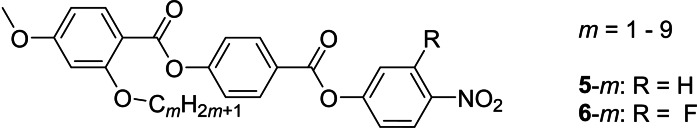
The structures of the **5** *‐m* and **6**‐*m* series where *m* refers to the number of carbon atoms in the lateral alkoxy chain.^16^

**Figure 3:**
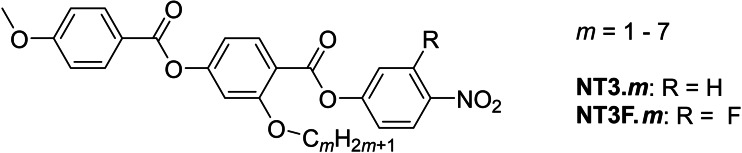
The structures of the NT3.*m* and NT3F.*m* series where *m* refers to the number of carbon atoms in the lateral alkoxy chain.

To enhance our understanding of the N_F_ phase and to exploit its application potential, new ferroelectric nematogens are required with improved temperature working ranges. To enable the rational design of these materials requires a better understanding of structure‐property relationships associated with the N_F_ phase but these remain at a very early stage. It is, therefore, critical that we now explore the effects that different structural modifications have on the stability of the N_F_ phase. Indeed, Mandle^[31]^ has recently reported hydrogen‐bonded supramolecular complexes that show the N_F_ phase and surprisingly, these have molecular dipole moments far smaller than the value of 9 D described earlier. Such studies will also provide further examples of the direct N_F_‐I transition which has only been observed for a rather limited number of molecules.[^6^, ^8^, ^11^, ^15^, ^16^, ^20^] With these goals in mind, here we report the synthesis and characterisation of two series of ferroelectric nematogens that are structurally analogous to the **5**‐*m* and **6**‐*m* series, Figure 2, that we recently reported,^[16]^ the (4‐nitrophenyl) 2‐alkoxy‐4‐(4‐methoxybenzoyl)oxybenzoates (NT3.*m*) and the (3‐fluoro‐4‐nitrophenyl) 2‐alkoxy‐4‐(4‐methoxybenzoyl)oxybenzoates (NT3F.*m*), Figure 3. The key difference between the **5**‐*m* and the **6**‐*m* series, and the NT3.*m* and NT3F.*m* series is that the lateral alkoxy chain is now attached to the central phenyl ring and this is expected to have a significant effect on the molecular shape. The NT3.*m* and NT3F.*m* series differ by a fluorine atom *ortho* to the terminal nitro group, and this allows the effects of molecular polarity and polarizability on the N_F_ phase to also be evaluated. We note that of the fourteen materials reported, three have been described previously.^7^

## Results and Discussion

The transitional properties of the NT3.*m* series are reported in Table 1. The transition temperatures of *m*=1–3 have been reported previously,^[7]^ and the data listed in Table 1 are in reasonable agreement with those in the literature. The value of *T* 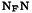 reported for NT3.3 is some 12 °C lower but, as we will see, this lower value perfectly fits the overall behaviour for the series. All the members of the NT3.*m* series showed a ferroelectric nematic phase preceded by a conventional monotropic nematic phase.

**Table 1:** Transition temperatures and associated scaled entropy changes for the NT3.*m* series.

*m*	T_CrI_/°C	*T* 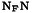 /°C	T_NI_/°C	▵S_CrI_/R	▵S_NFN_/R	▵S_NI_/R
1	192	–	–	12.5	–	–
	‐	126^[a]^	189^[a]^	‐	0.032^[a]^	0.21^[a]^
2	164	–	–	12.9	–	–
	–	104^[a]^	137^[a]^	‐	‐	0.25^[a]^
3	130	–	–	12.4	–	–
	–	93^[b]^	105^[a]^	–	–	0.25^[a]^
4	120	–	–	8.80	–	–
	–	76^[b]^	82^[a]^	–	–	0.13^[a]^
5	102	63	68	13.0	0.31	0.16
	–	61^[a]^	66^[a]^	–	0.30^[a]^	0.18^[a]^
6	94	55	58	12.9	–^[c]^	–^[c]^
	–	53^[a]^	57^[a]^	–	0.37^[a]^	0.13^[a]^
7	72	52	55	10.6	0.30	0.071
	–	51^[a]^	54^[a]^	–	0.36^[a]^	0.10^[a]^

[a] Values extracted from DSC cooling trace. [b] Measured using polarized optical microscopy. [c] Crystallisation precluded measurement of the entropy.

**Figure 4:**
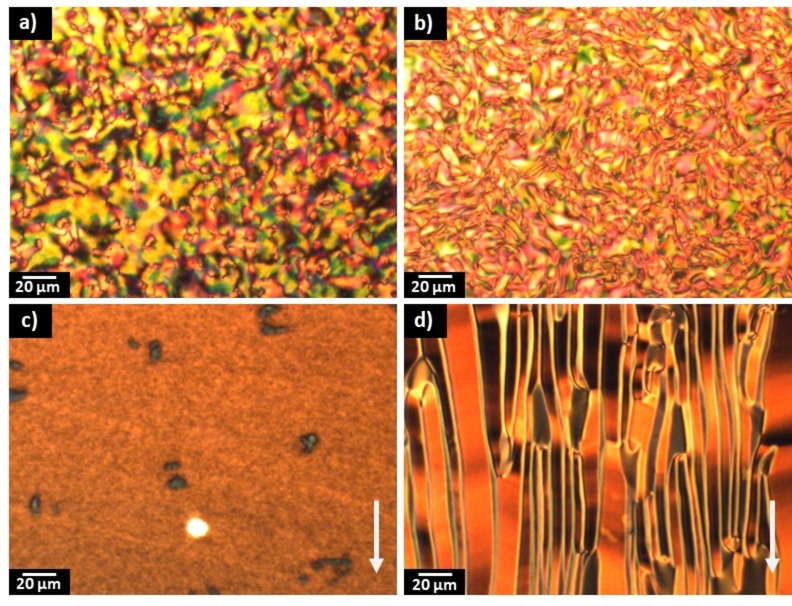
Polarized optical microscope textures observed for NT3.5: (a) Nematic schlieren texture between untreated glass slides (*T*=66 °C); (b) highly birefringent striped texture with boundaries in the N_F_ phase between untreated glass slides (*T*=61 °C); (c) uniform texture of the N phase in a planar aligned cell (*T*=66 °C); (d) banded texture of the N_F_ phase in a planar aligned cell (*T*=61 °C). The arrow shows the alignment direction.

The nematic phase was assigned by polarised optical microscopy based on the observation of characteristic schlieren textures which contained two and four‐point brush defects, together with the optical flickering associated with director fluctuations, Figure 4(a). Cooling the nematic phase saw a clear change in the birefringence of the sample along with the emergence of additional defect lines which acted as domain boundaries, Figure 4(b). These domains contained areas of differing birefringence and the boundary lines marked the division of regions where the director changed orientation and hence polarization. These domain walls have been described as “soft”,^[13]^ due to their ability to deform upon the application of a small electric field or to change position somewhat over time. In planar aligned cells, a uniform texture characteristic of the nematic phase, Figure 4(c), was followed by an increase in birefringence and the formation of a banded texture, characteristic of the ferroelectric nematic phase, Figure 4(d). The formation of these domains is thought to be driven by director twist deformations which are necessary to connect opposite polarization vectors on the lower and upper cell surfaces.^[15]^ The entropy changes associated with the nematic to isotropic transitions are lower than might be expected for low molar mass calamitic systems, but they are similar to those found for the **5**‐*m* series.^[16]^ The small values of Δ*S*_NI_/*R* found here for the NT3.*m* series have been attributed to the enhanced molecular biaxiality arising from the lateral alkoxy chain that reduces the orientational order of the nematic phase and hence, decreases *Δ*S_NI_/*R*.[^34^, ^35^]

**Figure 5:**
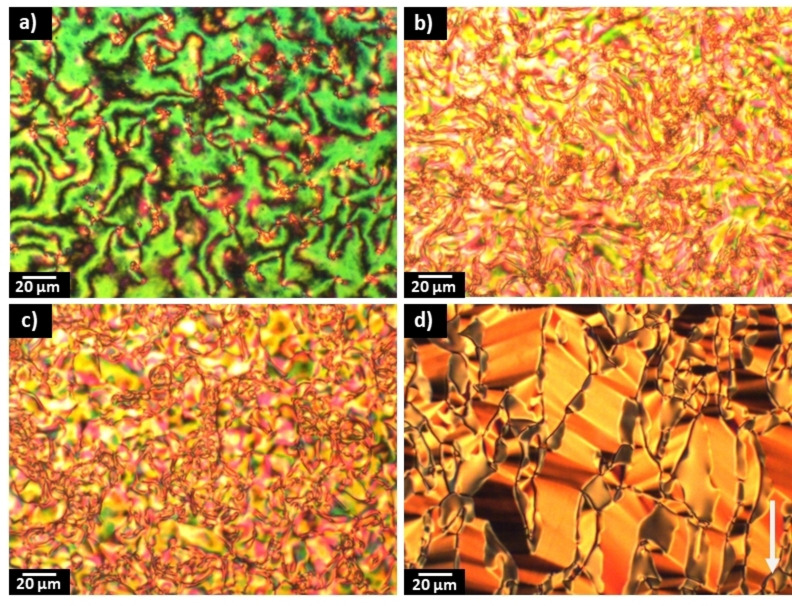
Polarized optical microscope textures: (a) Nematic schlieren texture of NT3F.1 between untreated glass slides (*T*=156 °C); (b) highly birefringent striped texture of NT3F.1 with boundaries in the N_F_ phase between untreated glass slides (*T*=141 °C); (c) highly birefringent texture with boundaries in the N_F_ phase of NT3F.5 between untreated glass slides (*T*=67 °C); (d) banded texture of the N_F_ phase for NT3F.5 in a planar aligned cell (*T*=67 °C). The arrow shows the alignment direction.

The transitional properties of the NT3F.*m* series are reported in Table 2. All the members of the NT3F.*m* series exhibited direct N_F_ to isotropic transitions, except for *m*=1 which showed a N phase preceding the N_F_ phase. The N and N_F_ phases seen for *m*=1, were assigned based on the observation of similar textures to those described for the NT3.*m* series, Figures 5(a–b). In the other members of the series the ferroelectric nematic phase developed directly from the isotropic liquid and in cells with planar anchoring this transition was marked by the emergence of spherical droplets which coalesced to give a banded texture with large domains, Figure 5(c–d). The scaled entropy changes associated with the N_F_‐I transitions for the NT3F.*m* series, Table 2, are on average six times larger than the scaled entropy changes associated with the N−I transitions for the NT3.*m* series, Table 1. This presumably reflects the additional entropic contribution associated with the ordering of the dipoles in the N_F_ phase compared to the conventional N phase and we have reported similar observations for other ferroelectric nematogens.[^6^, ^15^, ^16^]

**Table 2:** Transition temperatures and associated scaled entropy changes for the NT3F.*m* series.

*m*	T_CrI_/°C	*T* 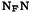 /°C *T 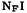 /°C	T_NI_/°C	▵S_CrI_/R	▵S_NFN_/R *▵S_NFI_/R	▵S_NI_/R
1	180	142	157	13.7	0.34	0.28
	–	141^[a]^	156^[a]^	–	0.34^[a]^	0.29^[a]^
2	179	‐	–	13.8	–	–
	–	118^[b]^*	–	–	–	–
3	135	‐	–	12.0	–	–
	–	92^[a]^*	–	–	0.91^[a]^*	–
4	124	‐	–	16.9	–	–
	–	79^[a]^*	–	–	1.52^[a]^*	–
5	104	70*	–	8.28	0.89*	–
	–	68^[a]^*	–	–	0.91^[a]^*	–
6	80	60*	–	12.6	–^[c]^*	–
	–	58^[a]^*	–	–	0.97^[a]^*	–
7	73	57*	–	12.9	0.93*	–
	–	55^[a]^*	–	–	0.96^[a]^*	–

[a] Values extracted from DSC cooling trace. [b] Measured using polarized optical microscopy. [c] Crystallisation precluded measurement of the entropy.

**Figure 6:**
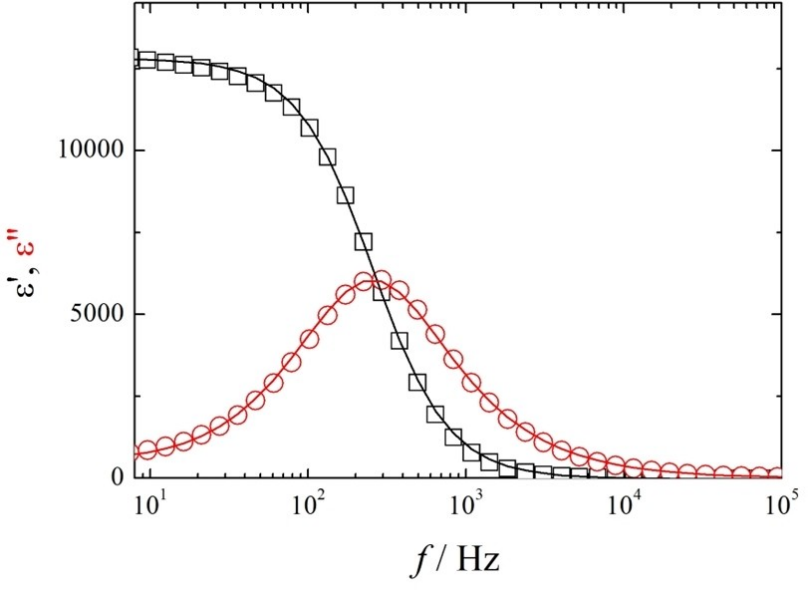
Complex dielectric permittivity measured for NT3F.7 at 52 °C in a 9.7‐μm‐thick cell with ITO electrodes and no alignment layer: the □ represents the measured real part of the dielectric permittivity, the ○ represents the imaginary part and the lines represent the fitting of the dispersion to the Cole‐Cole formula, where Δ*ϵ*=12860, *f*_r_=256 Hz, and *α*=0.04.

**Figure 7:**
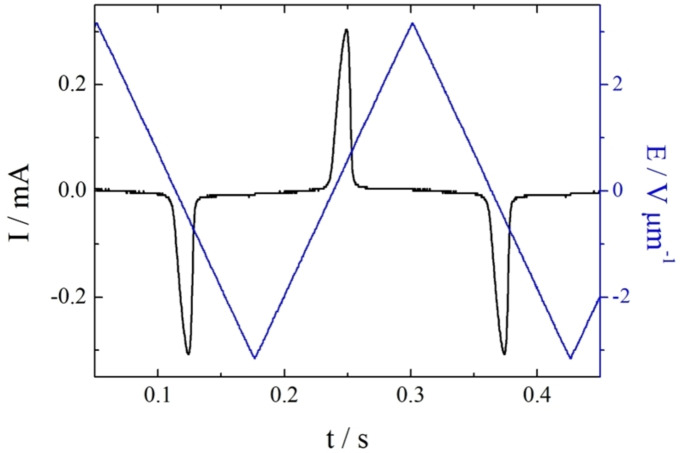
The switching current (black line) associated with polarization reversal under applied triangular wave voltage (blue line) for NT3F.7. Measurements were performed in a 9.7‐μm‐thick cell with ITO electrodes and no alignment layer.

Dielectric measurements were performed using NT3F.7. In the N_F_ phase a strong dielectrically active relaxation process was detected with dielectric strength reaching 13000 and relaxation frequency of the order of 100 Hz, Figures 6 and S1. This behaviour agrees well with that reported for other ferroelectric nematogens;[^6^, ^8^, ^11^, ^14^, ^15^, ^20^] the strong dielectric mode might be attributed to the collective movement of the polarization direction, phason mode.^[36]^ The polar character of the N_F_ phase was also confirmed by the observation of electric switching. Thus, when an AC voltage was applied to the cell, a single current peak per half cycle was registered and this is associated with the reversal of the spontaneous electric polarization, Figure 7. The Ps value for this material was found to be as high as 4.5 μC cm^−2^.

**Figure 8:**
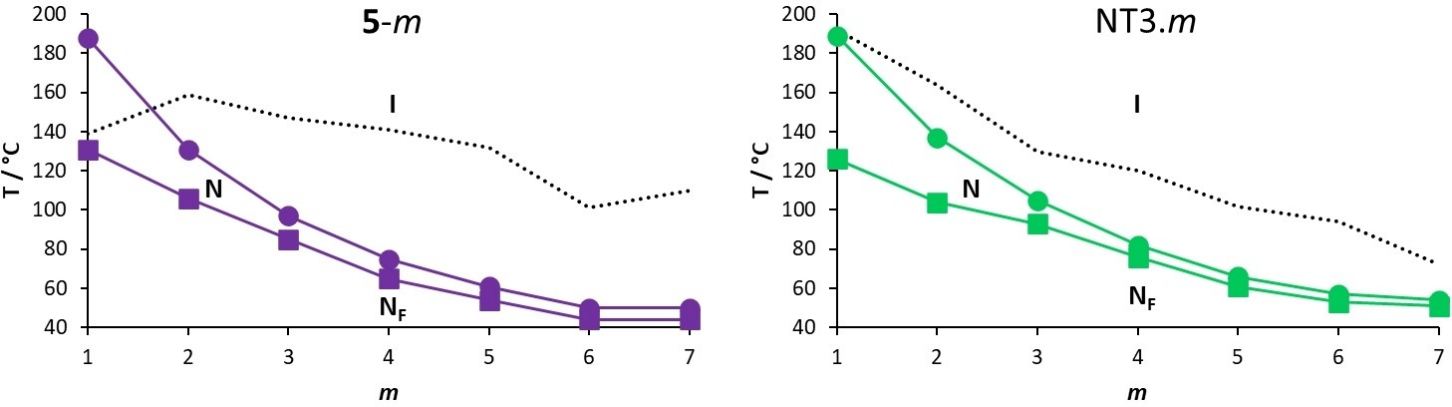
Dependence of the transition temperatures with respect to the number of carbon atoms in the lateral alkoxy chain, *m*, of the **5**‐*m* (purple symbols) and NT3.*m* (green symbols) series where ⋅⋅⋅ represents *T*_Cr‐_, • represents *T*_NI_, and ▪ *T* 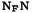 .

In Figure 8, the transition temperatures shown by the NT3.*m* series are compared to those of the corresponding series in which the lateral alkoxy chain is attached to the methoxy‐terminated terminal ring, **5‐** *m*.^[16]^ As the length of the lateral alkoxy chain in the NT3.*m* series is increased, the melting points decrease reflecting the disruption of the interactions between the molecules by the lateral substituent that also inhibits the packing of the molecules in the crystalline phase. These effects become more pronounced as the chain length is increased. The members with shorter lateral chains are more prone to crystallisation than those with longer terminal chains. The highest values of both *T*_NI_ and *T* 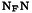 were found for *m*=1 and these values decreased as the length of the alkoxy chain increased, reaching limiting values for the longest homologues. Similar behaviour in terms of *T*_NI_ was first reported for low molar mass mesogens containing a lateral chain by Weissflog and Demus.[^37^, ^38^] It was suggested that the alkyl chain adopts conformations that allow it to lie along the molecular long axis and thus the shape anisotropy, for a given range of chain lengths, does not change and *T*_NI_ is effectively constant. Such an assumption is not, however, necessarily required to account for such behaviour.^[39]^ *T*_NI_ falls more quickly than *T* 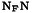 , on increasing the alkoxy chain length and the temperature range of the N phase narrows. The dependence of the transition temperatures on chain length of the NT3.*m* series is similar to that seen for the **5**‐*m* series.

**Figure 9:**
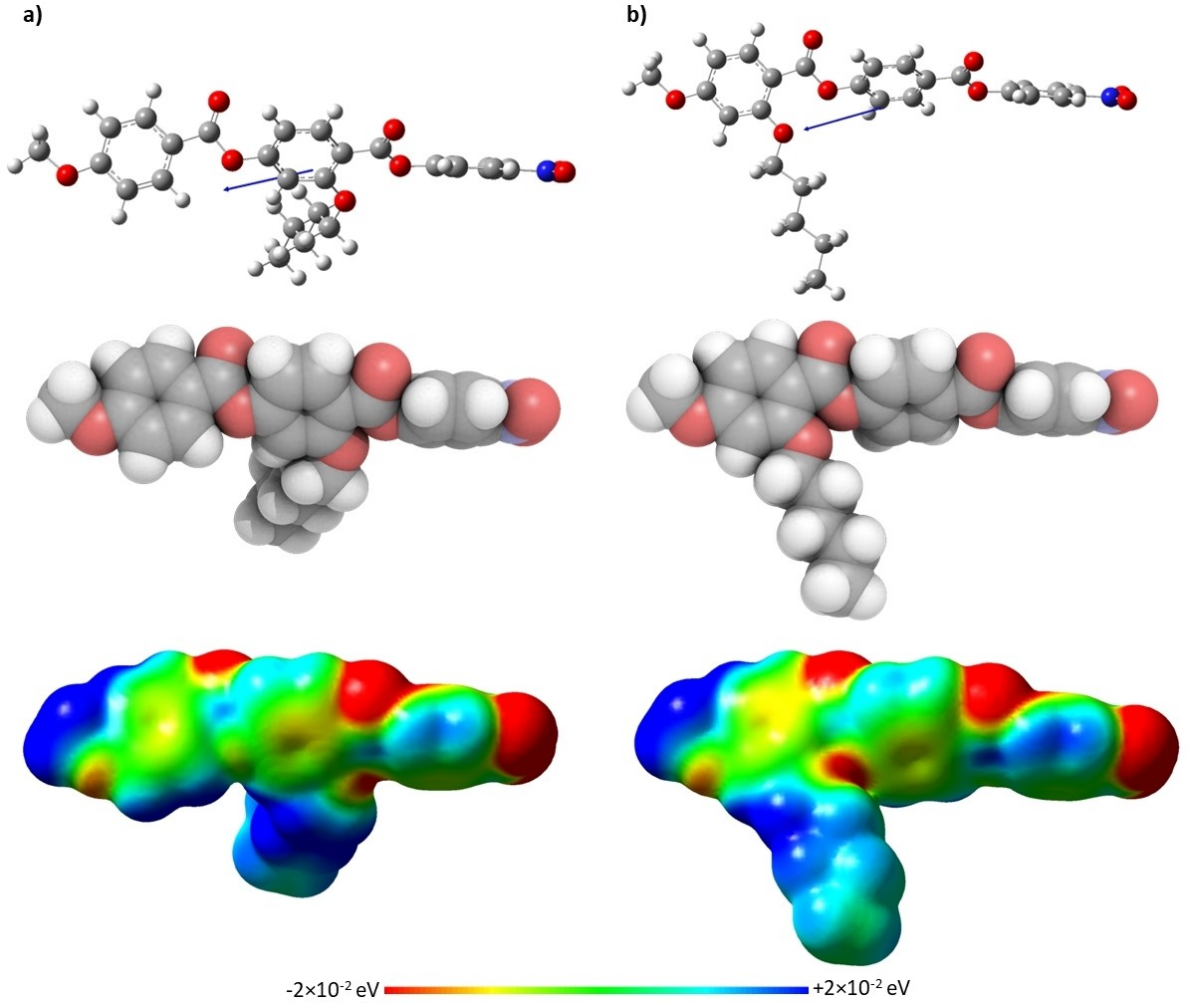
Ball‐and‐stick models (top), space‐filling models (middle) and electrostatic potential surfaces (bottom) of (a) NT3.5 and (b) **5‐**5, calculated at the B3LYP/6‐31(d) level of theory. The arrow indicates the positive direction of the calculated dipole moment.

The NT3.*m* series exhibits higher values of *T*_NI_ than the corresponding members of the **5**‐*m* series, although this difference was particularly small for *m*=1. This suggests that changing the position of the lateral chain from a terminal ring in the **5**‐*m* series to the middle ring in the NT3.*m* series enhances the structural anisotropy, albeit marginally. The values of *T* 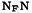 for the shortest lateral chains, *m*=1,2, are higher for the **5**‐*m* series, but on increasing *m* the NT3.*m* series showed higher values. This suggests that the stability of the N_F_ phase is a more subtle balance of shape and electronic effects and that these differ on increasing chain length. Figure 9 shows the ball‐and‐stick models, space‐filling models and associated electrostatic potential surfaces for NT3.5 and **5**‐5. Changing the position of the alkoxy chain clearly has a marked effect on molecular shape, and although **5**‐5 may be considered to have a tapered shape, NT3.5 cannot. The average molecular dipole moment of the NT3.*m* series is 11.67 D and this is around 0.45 D larger than for the corresponding members of the **5**‐*m* series. For example, for **5‐**5 the molecular dipole moment is 11.18 D compared to 11.72 D for NT3.5, the angle between the molecular axis and the molecular dipole is 13.3 ° for **5**‐5 and 19.2 ° for NT3.5. This increase in the angle between the dipole moment and the long molecular axis may be attributed to the increase in the dipole vector component along the y‐axis arising from the change in electron density on changing the position of the lateral chain from the methoxy‐substituted terminal ring in **5‐**5 to the middle ring in NT3.5. The angle found for **5**‐5 is smaller than that reported for RM734^[40]^ and this suggests that the extension of the lateral alkoxy chain contributes to this difference. It is important to note that although these calculations were undertaken with a different basis set than that used by Mandle et al.,^[40]^ we report an identical dipole moment for RM734 using the B3LYP/6‐31G(d) level of theory.^[15]^ Furthermore, the angle between the molecular dipole and the molecular axis was 15.8 ° using the B3LYP/6‐31G(d) level of theory, which is in good agreement with that found by Mandle et al.^[40]^ The molecular dipole moments of each member of the NT3.*m* series are given in Table SI7. It is apparent from the electrostatic potential surfaces that the ester linkages are isolating regions of electron density such that electronic conjugation does not extend along the molecular axis, Figure 9, and it has been suggested that such a distribution drives the formation of the N_F_ phase.^[31]^ In the framework of a molecular model developed by Madhusudana to describe the N_F_ phase, the calamitic molecules are considered to possess longitudinal surface charge density waves and these interact inhibiting the formation of antiparallel structures.^[41]^ This model also suggests that the parallel alignment of the molecules is enhanced by minimising the amplitude of the charge density wave at either end of the molecule. It is noteworthy that changing the position of the lateral alkoxy chain changes the electron distribution of the aromatic rings. In the case of **5**‐5, Figure 9, the lateral chain donates electrons into the terminal aromatic ring, increasing the amplitude of the charge density wave at the molecular terminus compared to that seen for NT3.5, and destabilises the ferroelectric nematic phase in accord with the predictions of the model proposed by Madhusudana.^[41]^

**Figure 10:**
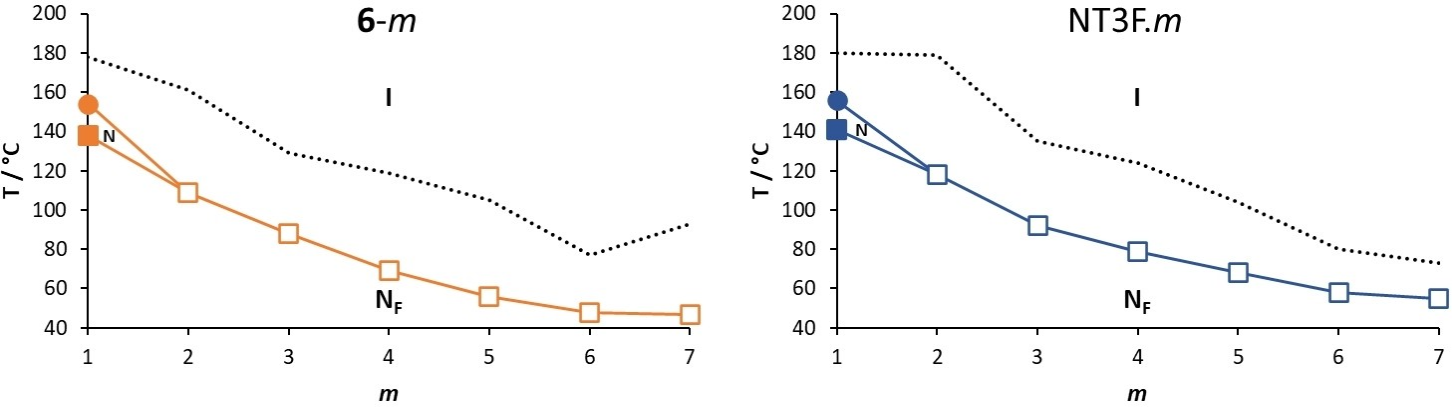
Dependence of the transition temperatures with respect to the number of carbon atoms in the lateral alkoxy chain, *m*, of the **6**‐*m* (orange symbols) and NT3F.*m* (blue symbols) series where ⋅⋅⋅ represents *T*_Cr‐_, • *T*_NI_, ▪ *T* 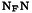 , and □ *T* 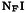 .

In Figure 10, the transition temperatures shown by the NT3F.*m* and **6**‐*m* series are compared.^[16]^ As with the NT3.*m* series, on increasing the length of the lateral alkoxy chain for the NT3F.*m* series, the melting points show a general decrease in temperature. As we discussed earlier, only NT3F.1 shows a conventional nematic phase. For this series the value of *T*_NI_ must fall sharply on increasing *m* and the remaining members show a N_F_‐I transition. This implies an increase in the stability of the N_F_ phase relative to that of the N phase on increasing *m*. The values of *T*_NI_ fall on increasing *m* reaching a limiting value for the longest homologues. The values of both *T*_NI_ and *T* 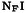 are higher for the NT3F.*m* series than for the corresponding members of the **6**‐*m* series.^[16]^ This also suggests that having the lateral chain attached to the middle aromatic ring is more favourable for the stability of the ferroelectric nematic phase than if it is located on the terminal ring as in the **6**‐*m* series. Again, this may be attributed to the difference in electron density in the methoxy‐substituted terminal ring arising from changing the position of the lateral alkoxy chain, as described earlier for the **5**‐*m* and NT3.*m* series. The average molecular dipole moment of the NT3F.*m* series is 12.75 D and this is around 0.55 D larger than for the corresponding members of the **6**‐*m* series. For example, for **6**‐4 the molecular dipole moment is 12.24 D compared to 12.79 D for NT3F.4, and the angle between the long molecular axis and the molecular dipole is 14.0 ° for **6**‐4 and 19.0 ° for NT3F.4. The difference in these angles is slightly smaller than that seen for **5**‐5 and NT3.5. The molecular dipole moments of each member of the NT3F.*m* series are given in Table SI7.

**Figure 11:**
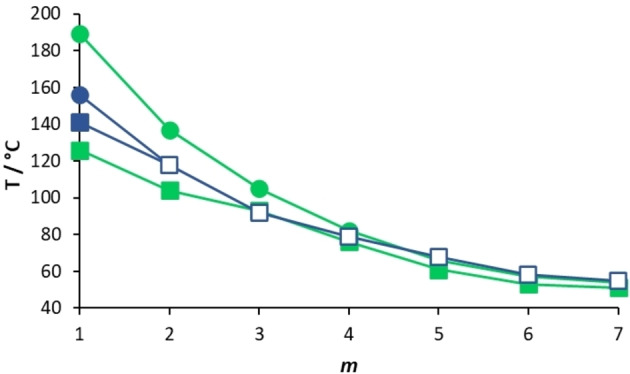
Dependence of the transition temperatures with respect to the number of carbon atoms in the lateral alkoxy chain, *m*, of the NT3.*m* (green symbols) and NT3F.*m* (blue symbols) series where • represents *T*_NI_, *▪ T* 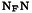 , and □ *T* 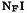 .

**Figure 12:**
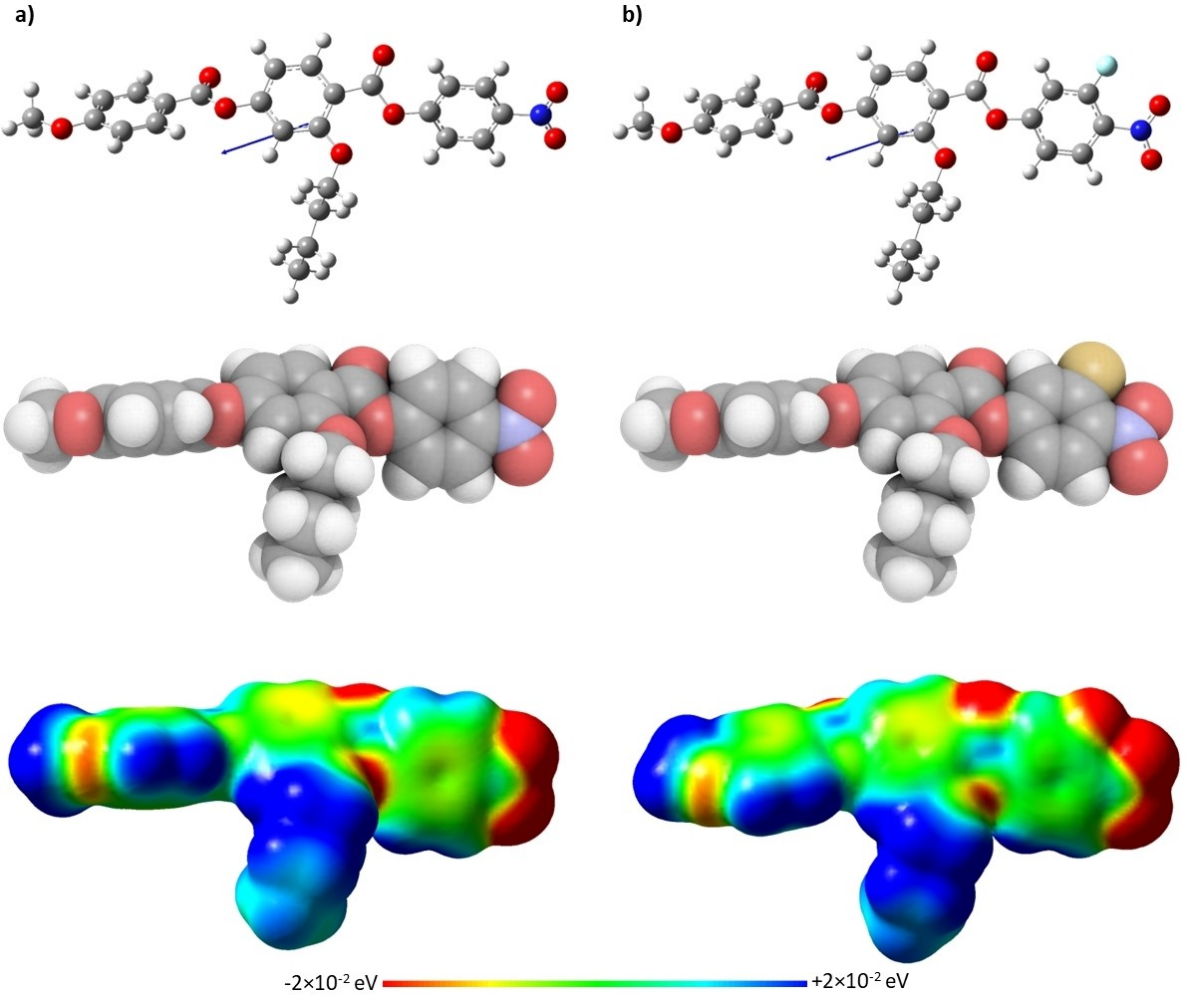
Ball‐and‐stick models (top), space‐filling models (middle) and electrostatic potential surfaces (bottom) of (a) NT3.4 and (b) NT3F.4, calculated at the B3LYP/6‐31(d) level of theory. The arrow indicates the positive direction of the calculated dipole moment.

A comparison of the transition temperatures of the NT3.*m* and NT3F.*m* series is shown in Figure 11. The striking difference between the two series is the loss of the conventional N phase in the NT3F.*m* series, except for *m*=1, whereas all members of the NT3.*m* series show the nematic phase. The fluorine atom *ortho* to the nitro group in NT3F.1 reduces *T*_NI_ by 35 °C compared to that of NT3.1. This reduction may be attributed to the decrease in shape anisotropy associated with replacing the smaller hydrogen atom by a larger fluorine atom in a lateral position, Figure 12. The size of this reduction of *T*_NI_ is very much in line with the values reported for similar materials.[^3^, ^16^] It is interesting to note that applying a similar value for the decrease in *T*_NI_ for the remaining members of the NT3F.*m* series, sees the predicted values fall well below the experimentally observed values of *T* 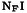 .

The values of *T* 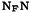 for the NT3.*m* and *T* 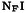 for the NT3F.*m* series show a similar dependence on increasing the length of the lateral alkoxy chain, Figure 11. Whereas *T*_NI_ decreased with the addition of a fluorine atom, the opposite behaviour is found comparing the transition temperatures of the N_F_ phase which are higher for the NT3F.*m* series. Similar behaviour was reported for the **5**‐*m* and **6**‐*m* series, and for materials based on similar structures.^[3]^ The increase in the stability of the ferroelectric nematic phase associated with the addition of the fluorine atom *ortho* to the terminal nitro group may be attributed to the change in polarity and polarizability of the molecule along with the change in shape.

The ball‐and‐stick models, space‐filling models, and electrostatic potential surfaces of NT3.4 and NT3F.4, are shown in Figure 12. Again, it is apparent that these materials cannot be described as being wedge‐shaped, and there is a clear difference in the electron density distribution in the aromatic ring containing the nitro group between the two molecules. This difference causes the average molecular dipole moment for the members of the NT3F.*m* series to be around 1.08 D higher than the NT3.*m* series. For example, the molecular dipole moments of NT3.4 and NT3F.4 are 11.69 D and 12.79 D, respectively, and the angles between the long molecular axis and the dipoles is 19.5 ° and 19.0 °, respectively. The addition of the fluorine atom *ortho* to the terminal nitro group, removes electron density from the nitro group and spreads the charge helping to stabilise the ferroelectric nematic phase, Figure 12, in accord with the model proposed by Madhusudana,^[41]^ and described earlier.

By comparing the behaviour of the **5**‐*m* and **6**‐*m* series with that of the NT3.*m* and NT3F.*m* series, respectively, it is clear that the change in the position of the alkoxy chain also contributed to the enhanced stability of the N_F_ phase. It is not clear, however, whether this is a steric effect, an electronic effect or as is perhaps more likely, a combination of both.

## Conclusions

We have reported the effects of changing the position of a lateral alkoxy chain from the methoxy‐substituted terminal ring to the central phenyl ring in two series of materials based on RM734. These materials cannot be described as being tapered and yet their values of *T* 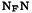 exceed those of the corresponding materials with the lateral alkoxy chain in the terminal ring. Indeed, this structural change is accompanied by both an absolute increase in *T* 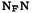 and a relative increase in *T* 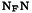 /*T*_NI_. In part, this may be attributed to electronic changes. Specifically, for the NT3.*m* series, the lateral alkoxy chain no longer donates electrons into the methoxy‐substituted terminal ring. This reduces the amplitude of the charge density wave compared to the corresponding members of the **5**‐*m* series, stabilising the ferroelectric nematic phase. It is possible, however, that for the design of new ferroelectric nematogens, the conventional approach of using a wedge‐shaped molecule is limiting, and rather than the shape, it is the role of the lateral chain in inhibiting anti‐parallel associations that is key. We clearly have a great deal more to learn about the structure‐property relationships in the remarkable N_F_ phase.

## Experimental Section

**Scheme 1:**
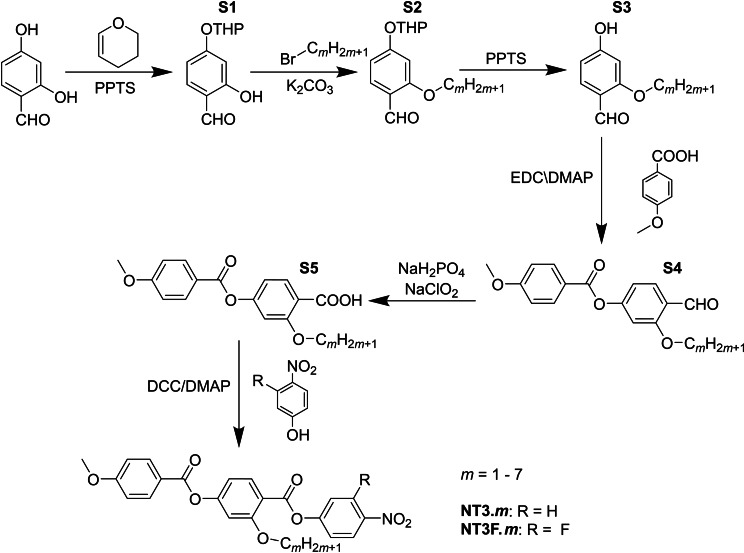
Overall synthetic route used to synthesise the (4‐nitrophenyl) 2‐alkoxy‐4‐(4‐methoxybenzoyl)oxybenzoates, NT3.*m* series and (3‐fluoro‐4‐nitrophenyl) 2‐alkoxy‐4‐(4‐methoxybenzoyl)oxybenzoates, NT3F.*m* series.

**Synthesis**: The synthetic route used to prepare the NT3.*m* and NT3F.*m* series is shown in Scheme 1. A detailed description of the preparation of both series, including the structural characterisation data for all intermediates and final products, is provided in the Supporting Information.

**Optical studies**: Phase characterisation was performed by polarised light microscopy, using an Olympus BH2 polarising light microscope equipped with a Linkam TMS 92 hot stage. The untreated glass slides were 0.17 mm thickness while the planar aligned cells were purchased from INSTEC with a cell thickness between 2.9–3.5 μm, and an ITO conducting layer.

**Differential scanning calorimetry**: The phase behaviour of the materials was studied by differential scanning calorimetry performed using Mettler Toledo DSC1 or DSC3 differential scanning calorimeters equipped with TSO 801RO sample robots and calibrated using indium and zinc standards. Heating and cooling rates were 10 °C min^−1^, with a 3‐min isotherm between either heating or cooling, and all samples were measured under a nitrogen atmosphere. Transition temperatures and associated enthalpy changes were extracted from the heating traces unless otherwise noted. For monotropic transitions, the sample was cooled to around 10 °C below the transition, held in a 3‐min isotherm, reheated and if crystallisation did not occur during either the cooling or isothermal segment, the transitional properties were extracted from the heating segment. The entropy changes associated with the transition temperatures were scaled with the universal gas constant, *R*, using a value of 8.314 J K^−1^ mol^−1^.

**Molecular modelling**: The geometric parameters of the NT3.*m* and NT3F.*m* series were obtained using quantum mechanical DFT calculations with Gaussian09 software.^[32]^ Optimisation of the molecular structures was carried out at the B3LYP/6‐31G(d) level of theory. A frequency check was used to confirm that the minimum energy conformation found was an energetic minimum. Visualisations of electronic surfaces and ball‐and‐stick models were generated from the optimised geometries using the GaussView 5 software. The electronic surfaces were found with the cubegen utility in GaussView by generating a total density cube using a SCF density matrix and course grid, overlayed by an ESP surface map. Visualisations of the space‐filling models were produced post‐optimisation using the QuteMol package.^[33]^

**Spontaneous electric polarization measurements**: Values of the spontaneous electric polarization were obtained from the current peaks recorded during Ps switching upon applying triangular voltage at a frequency of 2 Hz. The 9.7 μm‐thick cells with ITO electrodes and no polymer aligning layers were used and the switching current was determined by recording the voltage drop at the resistivity of 50 kOhm in serial connection with the sample. The current peak was integrated over time to calculate the surface electric charge and evaluate polarization value.

**Dielectric spectroscopy**: The complex dielectric permittivity, ϵ*, was studied using a Solatron 1260 impedance analyzer, measurements were conducted in 1 Hz–1 MHz frequency (*f*) range, with the probe voltage of 20 mV, and it was checked by optical observations that such a voltage is below the Fredericks transition threshold. The material was placed in 9.7 μm‐thick glass cells with ITO electrodes and no polymer aligning layers. Lack of a surfactant layer resulted in the random configuration of the director in the LC phases, microscopic observations of optical textures suggested dominant planar orientation without preferable direction of long molecular axis. The relaxation frequency, *f*_r_, and dielectric strength of the mode, Δ*ϵ*, were evaluated by fitting the complex dielectric permittivity to the Cole‐Cole formula:

$$\epsilon -\;\epsilon_{\infty }=\;\sum \frac{\Delta \epsilon }{{\left(1+\;\frac{if}{f_{r}}\right)}^{1-\;\alpha }}+i\left(\frac{\delta }{2\pi \epsilon_{0}f}\right)$$

where $\epsilon_{\infty }$ is the high frequency dielectric constant, *α* is the distribution parameter of the mode and *δ* is the low frequency conductivity, respectively.

## Conflict of interest

The authors declare no conflict of interest.

1

## Supporting information

As a service to our authors and readers, this journal provides supporting information supplied by the authors. Such materials are peer reviewed and may be re‐organized for online delivery, but are not copy‐edited or typeset. Technical support issues arising from supporting information (other than missing files) should be addressed to the authors.

Supporting Information

## Data Availability

The data that support the findings of this study are available in the supplementary material of this article.
