# Supplementary material for: Molecular Shape, Electronic Factors, and the Ferroelectric Nematic Phase: Investigating the Impact of Structural Modifications
Source: Chemistry. 2023 Mar 31;29(28):e202300073. doi: 10.1002/chem.202300073 (PMC10962687; doi:10.1002/chem.202300073)
Supplement: Supplementary file 1 — Supporting Information [file CHEM-29-0-s001.pdf]

Chemistry–A European Journal

Supporting Information

Molecular Shape, Electronic Factors, and the Ferroelectric Nematic Phase: Investigating the Impact of Structural Modifications

Naila Tufaha, Ewan Cruickshank,* Damian Pociecha, Ewa Gorecka, John M.D. Storey, and Corrie T. Imrie

Contents

General Information 2
Reagents 2
Thin Layer Chromatography 2
Column Chromatography 2
Structure Characterisation 2
Purity Analysis 2
Synthesis 3
2-Hydroxy-4-(oxan-2-yloxy)benzaldehyde (**S1**) 3
2-Alkoxy-4-(oxan-2-yloxy)benzaldehydes (**S2**) 4
2-Alkoxy-4-hydroxybenzaldehydes (**S3**) 6
(3-Alkoxy-4-formylphenyl) 4-methoxybenzoates (**S4**) 8
2-Alkoxy-4-(4-methoxybenzoyl)oxybenzoic acids (**S5**) 10
(3-Fluoro[a]-4-nitrophenyl) 2-alkoxy-4-(4-methoxybenzoyl)oxybenzoates (NT3.*m* – NT3F.*m*[a]) . 13
Additional Experimental Results 40
References 41

General Information

Reagents

All reagents and solvents that were available commercially were purchased from Sigma Aldrich, Fisher Scientific or Fluorochem and were used without further purification unless otherwise stated. Where required, solvents were dried over molecular sieves for a minimum of 24 hours prior to use.

Thin Layer Chromatography

Reactions were monitored using thin layer chromatography, and the appropriate solvent system, using aluminium-backed plates with a coating of Merck Kieselgel 60 F254 silica which were purchased from Merck KGaA. The spots on the plate were visualised by UV light (254 nm) or by oxidation using either a potassium permanganate stain or iodine dip.

Column Chromatography

For normal phase column chromatography, the separations were carried out using silica gel grade 60 Å, 40-63 µm particle size, purchased from Fluorochem and using an appropriate solvent system.

Structure Characterisation

All final products and intermediates that were synthesised were characterised using ^{1}H NMR, ^{13}C NMR and infrared spectroscopies. The ^{1}H and ^{13}C NMR spectra were recorded on a 400 MHz Bruker Advance III HD NMR spectrometer. The infrared spectra were recorded on a Perkin Elmer Spectrum Two FTIR with an ATR diamond cell.

Purity Analysis

In order to determine the purity of the final products, elemental analysis was used. C, H, N microanalysis were carried out by the Sheffield Analytical and Scientific Services Elemental Microanalysis Service at the University of Sheffield using an Elementar Vario MICRO Cube. The instrument was calibrated using series of different masses of sulphanilamide and acetanilide. High-resolution mass spectrometry was carried out at the University of Aberdeen by Dr Morag Douglas using a Waters XEVO G2 Q-Tof mass spectrometer. The instrument was calibrated with sodium formate, and the lock mass was leucine enkephalin, Formula: $C_{28}H_{37}N_5O_7$, $[M+H]^+$: 556.2771.

Synthesis

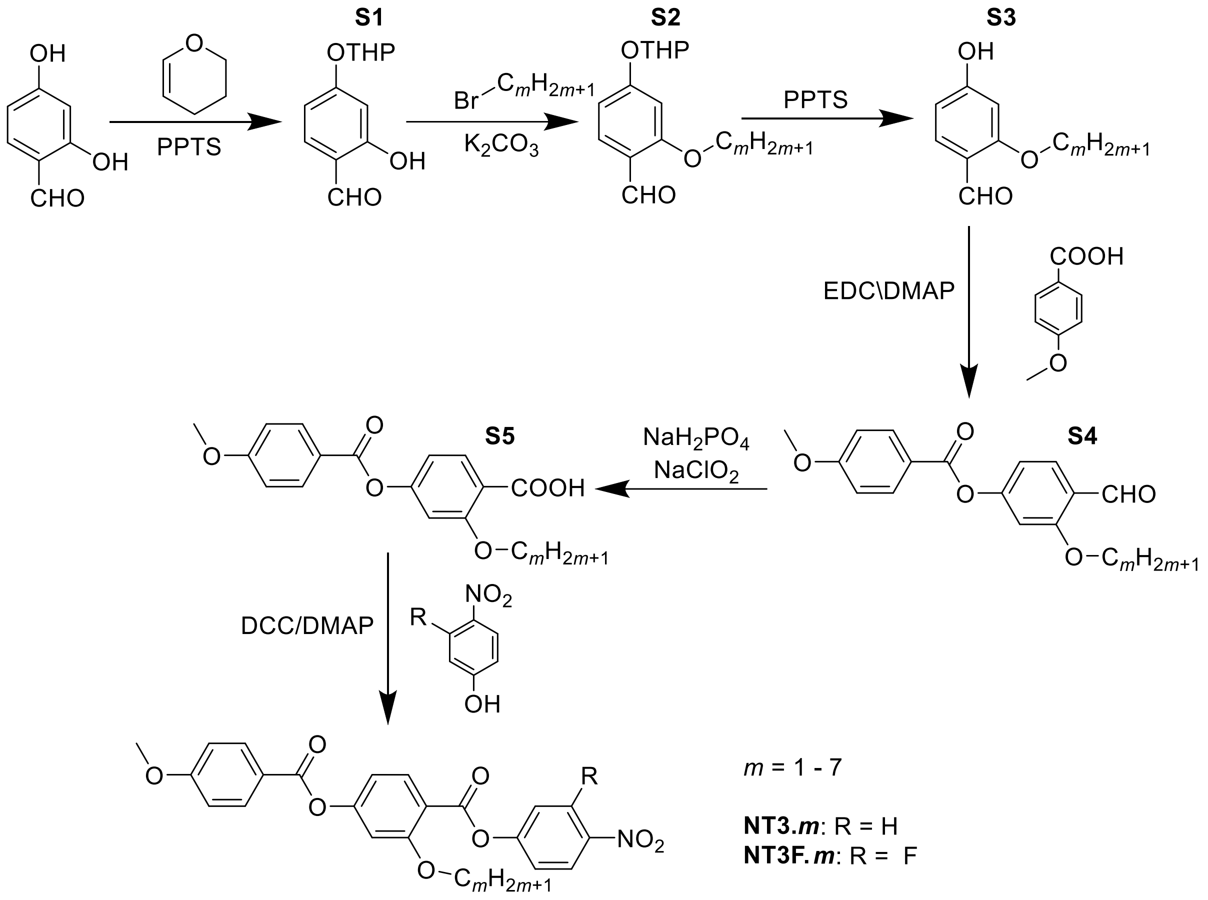

Scheme 1: Overall synthetic route used to synthesise the (4-nitrophenyl) 2-alkoxy-4-(4-methoxybenzoyl)oxybenzoates, NT3.*m* series, and (3-fluoro-4-nitrophenyl) 2-alkoxy-4-(4-methoxybenzoyl)oxybenzoates, NT3F.*m* series.

2-Hydroxy-4-(oxan-2-yloxy)benzaldehyde (**S1**)

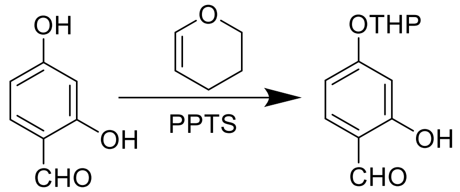

Scheme 2: Synthesis of the 2-hydroxy-4-(oxan-2-yloxy)benzaldehyde intermediates.

Under inert conditions, to a solution of 2,4-dihydroxybenzaldehyde (1 eq, 10 g, 7.24×10^{-2} mol) and 3,4-dihydropyran (1.2 eq, 7.3 g, 7.9 mL, 8.68×10^{-2} mol) in 30 mL dichloromethane (DCM), pyridinium p-toluenesulfonate (0.1 eq, 1.8 g, 7.16×10^{-3} mol) was added dissolved in 5 mL of DCM. The reaction mixture was allowed to react for 2 h and quenched by the addition of aqueous $NaHCO_3$. The mixture was extracted with dichloromethane (3 × 50 mL) and then dried with $MgSO_4$. The drying agent was filtered, and the solvent removed *in vacuo*. The product was then purified by flash column chromatography using a mixture of ethyl acetate and hexane (5:95) to obtain the pure compound as a colourless oil with a yield of 65%.

RF: 0.38 (10:90 EtOAc:Hex)

IR cm^{-1}: 3228 (-OH), 2945 (-CH_2), 2854 (-CH), 1627 (RC(=O)H), 1506 (C=C aryl), 1426, 1340, 1283, 1218, 1103, 1086, 952, 906, 867, 830, 772, 712, 626, 591, 544, 479, 458, 420.
^{1}H NMR (400 MHz, DMSO) δ ppm: 11.35 (s, 1H, -HCO), 9.71 (s, 1H, OH), 7.42 (d, J = 8.6 Hz, 1H, Ar), 6.64 (dd, J = 8.6, 2.2 Hz, 1H, Ar), 6.61 (d, J = 2.3 Hz, 1H, Ar), 5.49 (t, J = 3.2 Hz, 1H, THP), 3.81 (ddd, J = 11.2, 9.9, 3.1 Hz, 1H, THP), 3.62 (dtd, J = 11.4, 4.1, 1.4 Hz, 1H, THP), 1.98 – 1.41 (m, 6H, THP).
^{13}C NMR (101 MHz, DMSO) δ ppm: 194.69, 164.46, 164.29, 135.41, 115.87, 109.52, 103.79, 96.35, 62.31, 30.07, 25.08, 18.55.
Data consistent with reported literature.[1]

2-Alkoxy-4-(oxan-2-yloxy)benzaldehydes (**S2**)

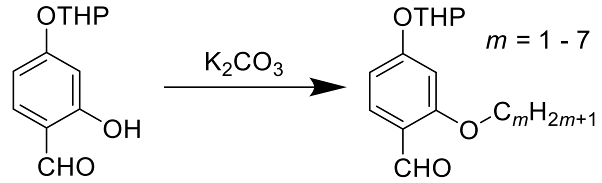

Scheme 3: Synthesis of the 2-alkoxy-4-(oxan-2-yloxy)benzaldehyde intermediates.

A two-neck round bottom flask is charged with **S1** (1 eq) and potassium carbonate (1.2 eq) under inert conditions. The solids were dissolved in dimethylformamide (DMF) and the required 1-bromoalkane chain (1.2 eq) was injected. The quantities of reagents used in the reaction are listed in Table SI1. The reaction was allowed to proceed overnight at 60 °C. After cooling, the reaction mixture was diluted with water, extracted with ethyl acetate (3 × 50 mL) and then dried with $MgSO_4$. The drying agent was filtered, and the solvent removed *in vacuo* to obtain the pure compound without further purification.

Table SI1: Quantities of reagents used in the synthesis of 2-alkoxy-4-(oxan-2-yloxy)benzaldehyde intermediates, **S2**.*m*.

Product name	**S1**	1-Bromoalkane	Potassium Carbonate
S2.2	4.00 g, 1.79×10^{-2} mol	2.28 g, 1.56 mL, 2.16×10^{-2} mol	2.97 g, 2.16×10^{-2} mol
S2.3	3.00 g, 1.34×10^{-2} mol	1.98 g, 1.46 mL, 1.61×10^{-2} mol	2.23 g, 1.61×10^{-2} mol
S2.4	8.84 g, 3.97×10^{-2} mol	6.54 g, 5.15ml, 4.77×10^{-2} mol	6.60 g, 4.77×10^{-2} mol
S2.5	11.0 g, 4.90×10^{-2} mol	8.88 g, 7.29 mL, 5.80×10^{-2} mol	8.17 g, 5.90×10^{-2} mol
S2.6	10.0 g, 4.47×10^{-2} mol	6.60 g, 5.62 mL, 5.37×10^{-2} mol	8.87 g, 5.37×10^{-2} mol
S2.7	6.00 g, 2.69×10^{-2} mol	5.77 g, 5.06 mL, 3.23×10^{-2} mol	5.32 g, 3.23×10^{-2} mol

2-Ethoxy-4-(oxan-2-yloxy)benzaldehyde (S2.2)

Yield: 4.27 g, 95%. RF: 0.45 (20:80 EtOAc:Hex)
Colourless oil
IR cm^{-1}: 2941 (-CH_2), 2852 (-CH), 1674 (RC(=O)H), 1596 (Aryl C=C), 1575, 1498, 1436, 1389, 1356, 1253, 1173, 1097, 1034 (C-O-C), 996, 948, 912, 872, 817, 675, 605, 574, 462.
^{1}H NMR (400 MHz, DMSO) δ ppm: 10.21 (s, 1H, -HCO), 7.65 (d, J = 8.6 Hz, 1H, Ar), 6.75 (d, J = 2.1 Hz, 1H, Ar), 6.72 (dd, J = 8.6, 2.2, 1H, Ar), 5.65 (t, J = 3.1 Hz, 1H, THP), 4.16 (qd, J = 6.9, 2.9 Hz, 2H, -O$\underline{CH_2}$$CH_3$), 3.72 (m, 1H, THP), 3.59 (m, 1H, THP), 1.94 – 1.47 (m, 6H, THP), 1.38 (t, J = 7.0 Hz, 3H, -O$CH_2$$\underline{CH_3}$).

^{13}C NMR (101 MHz, DMSO) δ ppm: 187.89, 163.72, 163.09, 129.89, 119.19, 108.95, 101.48, 96.05, 64.62, 62.10, 29.95, 25.00, 18.75, 14.82.
Data consistent with reported literature.[1]

2-Propoxy-4-(oxan-2-yloxy)benzaldehyde (S2.3)

Yield: 3.76 g, 95%. RF: 0.45 (20:80 EtOAc:Hex)
Colourless oil
IR cm^{-1}: 2978 (CH_3), 2942 ($-CH_2$), 2852 (-CH), 1738, 1676 (RC(=O)H), 1597 (Aryl C=C), 1436, 1390, 1254, 1174, 1099, 1035 (C-O-C), 949, 914, 818, 676, 606, 575, 464.
^{1}H NMR (400 MHz, DMSO) δ ppm: 10.23 (s, 1H, -HCO), 7.65 (d, J = 8.6 Hz, 2H, Ar), 6.76 (d, J = 2.1 Hz, 1H, Ar), 6.71 (dd, J = 8.6, 2.1 Hz, 1H, Ar), 5.66 (t, J = 3.0 Hz, 1H, THP), 4.12 – 4.00 (m, 2H, $-O\underline{CH_2}CH_2CH_3$) 3.80 – 3.67 (m, 1H, THP), 3.64 – 3.54 (m, 1H, THP), 1.94 – 1.70 (m, 6H, THP), 1.68 – 1.44 (m, 2H, $-OCH_2\underline{CH_2}CH_3$), 1.00 (t, J = 7.4 Hz, 3H, $-OCH_2CH_2\underline{CH_3}$).
^{13}C NMR (101 MHz, DMSO) δ ppm: 188.36, 163.70, 163.27, 129.85, 119.44, 108.41, 100.32, 96.10, 69.94, 61.97, 30.03, 25.02, 22.40, 18.36, 10.54.
Data consistent with reported literature.[1]

2-Butoxy-4-(oxan-2-yloxy)benzaldehyde (S2.4)

Yield: 11.0 g, 100%. RF: 0.52 (20:80 EtOAc:Hex)
Yellow oil
IR cm^{-1}: 2939 ($-CH_2$), 2873 (-CH), 1738, 1676 (RC(=O)H), 1597 (Aryl C=C), 1435, 1387, 1254, 1173, 1099, 1036 (C-O-C), 1021, 962, 905, 808, 676, 606, 463.
^{1}H NMR (400 MHz, DMSO) δ ppm: 10.22 (s, 1H, -HCO), 7.65 (d, J = 8.6 Hz, 1H, Ar), 6.79 – 6.68 (m, 2H, Ar), 5.66 (t, J = 3.1 Hz, 1H, THP), 4.10 (m, 2H, $-O\underline{CH_2}CH_2CH_2CH_3$), 3.73 (m, 1H, THP), 3.60 (m, 1H, THP), 1.92 – 1.69 (m, 4H, THP), 1.60 (m, 2H, THP), 1.51 – 1.41 (m, 4H, $-OCH_2\underline{CH_2}\underline{CH_2}CH_3$), 0.95 (t, J = 7.4 Hz, 3H, $-OCH_2CH_2CH_2\underline{CH_3}$).
^{13}C NMR (101 MHz, DMSO) δ ppm: 187.32, 163.27, 162.79, 129.41, 118.78, 108.49, 100.98, 95.55, 68.05, 61.60, 59.75, 30.50, 29.48, 24.54, 18.73, 13.66.

2-Pentoxy-4-(oxan-2-yloxy)benzaldehyde (S2.5)

Yield: 14.4 g, 100%. RF: 0.52 (20:80 EtOAc:Hex)
Orange oil
IR cm^{-1}: 2936 ($-CH_2$), 2871 (-CH), 1677 (RC(=O)H), 1596 (Aryl C=C), 1435, 1389, 1254, 1172, 1098, 1036 (C-O-C), 1020, 980, 907, 817, 676, 605, 584, 462.
^{1}H NMR (400 MHz, DMSO) δ ppm: 10.21 (s, 1H, -HCO), 7.64 (d, J = 8.6 Hz, 1H, Ar), 6.75 (d, J = 2.1 Hz, 1H, Ar), 6.71 (dd, J = 8.9, 1.8 Hz, 1H, Ar), 5.65 (t, J = 3.1 Hz, 1H, THP), 4.09 (t, J = 6.4 Hz, 2H, $-O\underline{CH_2}CH_2CH_2CH_2CH_3$), 3.72 (m, 1H, THP), 3.63 – 3.47 (m, 1H, THP), 1.95 – 1.68 (m, 6H, THP, $-OCH_2\underline{CH_2}CH_2CH_2CH_3$), 1.68 – 1.47 (m, 2H, THP), 1.48 – 1.27 (m, 4H, $-OCH_2CH_2\underline{CH_2}\underline{CH_2}CH_3$), 0.89 (t, J = 7.1 Hz, 3H, $-OCH_2CH_2CH_2CH_2\underline{CH_3}$).
^{13}C NMR (101 MHz, DMSO) δ ppm: 187.38, 163.29, 162.82, 129.45, 118.79, 108.53, 101.01, 95.58, 68.37, 61.64, 29.50, 28.12, 27.69, 24.56, 21.86, 18.28, 13.92.

2-Hexoxy-4-(oxan-2-yloxy)benzaldehyde (S2.6)

Yield: 12.4 g, 90%. RF: 0.56 (20:80 EtOAc:Hex)
Brown oil
IR cm^{-1}: 2932 ($-CH_2$), 2855 (-CH), 1676 (RC(=O)H), 1598 (Aryl C=C), 1498, 1436, 1386, 1254, 1173, 1099, 1036 (C-O-C), 1020, 950, 903, 817, 658, 585, 463.
^{1}H NMR (400 MHz, DMSO): 10.21 (s, 1H, -HCO), 7.64 (d, J = 8.5 Hz, 1H, Ar), 6.75 (d, J = 2.1 Hz, 1H, Ar), 6.74 – 6.68 (dd, 1H, Ar), 5.65 (q, J = 3.0 Hz, 1H, THP), 4.13 (m, 2H, $-O\underline{CH_2}CH_2CH_2CH_2CH_2CH_3$), 3.72 (m, 1H, THP), 3.64 – 3.54 (m, 1H, THP), 1.92 – 1.68 (m, 6H, THP, $-OCH_2\underline{CH_2}CH_2CH_2CH_2CH_3$), 1.70 – 1.49 (m, 2H, THP), 1.43 (m, 2H, $-OCH_2CH_2\underline{CH_2}CH_2CH_2CH_3$), 1.31 (m, 4H, $-OCH_2CH_2CH_2\underline{CH_2}\underline{CH_2}CH_3$), 0.87 (q, J = 6.6 Hz, 3H, $-OCH_2CH_2CH_2CH_2CH_2\underline{CH_3}$).

^{13}C NMR (101 MHz, DMSO) δ ppm: 187.31, 163.26, 162.78, 129.40, 118.76, 108.49, 100.99, 95.54, 68.34, 61.60, 30.91, 29.47, 28.35, 25.11, 24.51, 22.02, 18.25, 13.86.

2-Heptoxy-4-(oxan-2-yloxy)benzaldehyde (S2.7)

Yield: 8.61 g, 100%. RF: 0.59 (20:80 EtOAc:Hex)
Brown oil
IR cm^{-1}: 2928 ($-CH_2$), 2854 (-CH), 1738, 1676 (RC(=O)H), 1598 (Aryl C=C), 1498, 1467, 1435, 1387, 1255, 1203, 1172, 1098, 1035 (C-O-C), 1019, 952, 904, 872, 817, 676, 658, 605, 462.
^{1}H NMR (400 MHz, DMSO) δ ppm: 10.21 (s, 1H, -HCO), 7.64 (d, J = 8.6 Hz, 1H, Ar), 6.76 (d, J = 2.2 Hz, 1H, Ar), 6.71 (dd, J = 8.7, 2.1 Hz, 1H, Ar), 5.66 (t, J = 3.1 Hz, 2H, THP), 4.20 – 4.06 (m, 2H, $-O\underline{CH_2}CH_2CH_2CH_2CH_2CH_2CH_3$), 3.77 – 3.67 (m, 1H, THP), 3.64 – 3.55 (m, 1H, THP), 1.96 – 1.70 (m, 4H, THP), 1.66 – 1.49 (m, 4H, THP, $-OCH_2\underline{CH_2}CH_2CH_2CH_2CH_2CH_3$), 1.48 – 1.23 (m, 8H, $-OCH_2CH_2\underline{CH_2CH_2CH_2CH_2}CH_3$), 0.86 (t, J = 6.9 Hz, 3H, $-OCH_2CH_2CH_2CH_2CH_2CH_2\underline{CH_3}$).
^{13}C NMR (101 MHz, DMSO) δ ppm: 187.27, 163.26, 162.78, 129.37, 118.76, 108.47, 100.96, 95.53, 68.33, 61.58, 31.20, 29.47, 28.39, 28.37, 25.43, 24.53, 22.01, 18.24, 13.89.

2-Alkoxy-4-hydroxybenzaldehydes (**S3**)

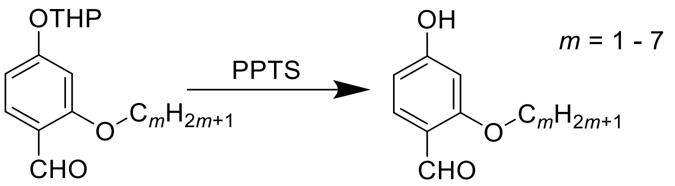

Scheme 4: Synthesis of the 2-alkoxy-4-hydroxybenzaldehyde intermediates.

To a solution of **S2** (1 eq) in tetrahydrofuran/ethanol (1:1), solid pyridinium p-toluenesulfonate (1.5 eq) was added. The quantities of reagents used in the reaction are listed in Table SI2. The reaction mixture was allowed to react at reflux overnight and then quenched by evaporation of the solvent to dryness. The reaction crude was dissolved in dichloromethane and washed with water and brine. The organic layer was dried with $MgSO_4$, the drying agent was filtered, and the solvent removed *in vacuo*. The product was then purified by flash column chromatography using a mixture of ethyl acetate and hexane (50:50).

Table SI2: Quantities of reagents used in the synthesis of 2-alkoxy-4-hydroxybenzaldehyde intermediates, **S3**.*m*.

Product name	**S2**.*m*	Pyridinium p-toluenesulfonate
S3.2	4.00 g, 1.60×10^{-2} mol	6.05 g, 2.40×10^{-2} mol
S3.3	3.76 g, 1.49×10^{-2} mol	2.67 g, 2.24×10^{-2} mol
S3.4	10.0 g, 3.61×10^{-2} mol	13.6 g, 5.41×10^{-2} mol
S3.5	14.0 g, 4.77×10^{-2} mol	18.0 g, 7.15×10^{-2} mol
S3.6	11.7 g, 3.81×10^{-2} mol	14.4 g, 5.72×10^{-2} mol
S3.7	8.61 g, 2.69×10^{-2} mol	10.1 g, 4.03×10^{-2} mol

2-Ethoxy-4-hydroxybenzaldehyde (S3.2)

Yield: 2.12 g, 80%. RF: 0.11 (20:80 EtOAc:Hex)
M.P. = 167 °C Yellow crystal
IR cm^{-1}: 3015 (-OH), 2980 (CH_3), 2876 ($-CH_2$), 2805, 2707, 2585, 1639 (RC(=O)H), 1615, 1568 (Aryl C=C), 1466, 1407, 1283, 1246, 1185, 1105, 1033 (C-O-C), 804, 737, 654, 584, 502, 461, 410.
^{1}H NMR (400 MHz, DMSO) δ ppm: 10.62 (s, 1H, Ar-OH), 10.14 (s, 1H, -HCO), 7.56 (d, J = 8.4 Hz, 1H, Ar), 6.50 – 6.41 (m, 2H, Ar), 4.10 (q, J = 6.9 Hz, 2H, $-O\underline{CH_2}CH_3$), 1.37 (t, J = 6.9 Hz, 3H, $-OCH_2\underline{CH_3}$).

^{13}C NMR (101 MHz, DMSO) δ ppm: 186.89, 165.13, 163.15, 129.77, 116.99, 108.47, 99.52, 63.82, 14.39.
Data consistent with reported literature.[1]

2-Propoxy-4-hydroxybenzaldehyde (S3.3)
Yield: 1.30 g, 48%. RF: 0.13 (20:80 EtOAc:Hex)
M.P. = 167 °C Pink crystal
IR cm^{-1}: 3126 (-OH), 2968 (CH_3), 2877 ($-CH_2$), 1639 (RC(=O)H), 1598, 1575 (Aryl C=C), 1455, 1391, 1318, 1277, 1185, 1103, 1040 (C-O-C), 1015, 972, 820, 763, 742, 658, 584, 511, 465.
^{1}H NMR (400 MHz, DMSO) δ ppm: 10.61 (s, 1H, Ar-OH), 10.16 (s, 1H, -HCO), 7.56 (d, J = 8.4 Hz, 1H, Ar), 6.51 – 6.37 (m, 2H, Ar), 3.99 (t, J = 6.4 Hz, 2H, $-O\underline{CH_2}CH_2CH_3$), 1.77 (h, J = 6.3 Hz, 2H, $-OCH_2\underline{CH_2}CH_3$), 0.99 (t, J = 7.4 Hz, 3H, $-OCH_2CH_2\underline{CH_3}$).
^{13}C NMR (101 MHz, DMSO) δ ppm: 186.85, 165.16, 163.34, 129.81, 117.10, 108.49, 99.54, 69.48, 21.89, 10.42.
Data consistent with reported literature.[1]

2-Butoxy-4-hydroxybenzaldehyde (S3.4)
Yield: 4.99 g, 71%. RF: 0.13 (20:80 EtOAc:Hex)
M.P. = 88 °C Brown crystal
IR cm^{-1}: 3068 (-OH), 2954 (CH_3), 2933 ($-CH_2$), 2868 ($-CH_2$), 1637 (RC(=O)H), 1598, 1574 (Aryl C=C), 1458, 1407, 1322, 1276, 1184, 1113, 1056 (C-O-C), 1020, 992, 835, 820, 656, 598, 502, 462.
^{1}H NMR (400 MHz, DMSO) δ ppm: 10.49 (s, 1H, Ar-OH), 10.14 (s, 1H, -HCO), 7.56 (d, J = 8.5, 1H, Ar), 6.50 – 6.41 (m, 2H, Ar), 4.04 (t, J = 6.3 Hz, 2H, $-O\underline{CH_2}CH_2CH_2CH_3$), 1.74 (dq, J = 8.3, 6.4 Hz, 2H, $-OCH_2\underline{CH_2}CH_2CH_3$), 1.53 – 1.37 (m, 2H, $-OCH_2CH_2\underline{CH_2}CH_3$), 0.98 – 0.90 (m, 3H, $-OCH_2CH_2CH_2\underline{CH_3}$).
^{13}C NMR (101 MHz, DMSO) δ ppm: 186.84, 165.20, 163.34, 129.79, 117.08, 108.50, 99.54, 67.76, 30.52, 18.78, 13.70.

2-Pentoxy-4-hydroxybenzaldehyde (S3.5)
Yield: 11.0 g, 100%. RF: 0.17 (20:80 EtOAc:Hex)
M.P. = 109 °C Brown crystal
IR cm^{-1}: 3070 (-OH), 2953 (CH_3), 2926 ($-CH_2$), 2870 ($-CH_2$), 1639 (RC(=O)H), 1596, 1574 (Aryl C=C), 1454, 1392, 1321, 1277, 1235, 1187, 1101, 1012, 838, 821, 771, 734, 659, 589, 589, 509, 467, 439.
^{1}H NMR (400 MHz, DMSO) δ ppm: 10.61 (s, 1H, Ar-OH), 10.14 (s, 1H, -HCO), 7.56 (d, J = 8.4 Hz, 1H, Ar), 6.49 – 6.41 (m, 2H, Ar), 4.03 (t, J = 6.8 Hz, 2H, $-O\underline{CH_2}CH_2CH_2CH_2CH_3$), 1.81 – 1.67 (m, 2H, $-OCH_2\underline{CH_2}CH_2CH_2CH_3$), 1.52 – 1.27 (m, 4H, $-OCH_2CH_2\underline{CH_2CH_2}CH_3$), 0.89 (t, J = 7.1 Hz, 3H, $-OCH_2CH_2CH_2CH_2\underline{CH_3}$).
^{13}C NMR (101 MHz, DMSO) δ ppm: 186.79, 165.16, 163.32, 129.74, 117.06, 108.47, 99.51, 68.03, 28.12, 27.70, 21.85, 13.90.

2-Hexoxy-4-hydroxybenzaldehyde (S3.6)
Yield: 9.61 g, 100%. RF: 0.20 (20:80 EtOAc:Hex)
M.P. = 106 °C Brown crystal
IR cm^{-1}: 3182 (-OH), 2932 ($-CH_2$), 2870 ($-CH_2$), 1660 (RC(=O)H), 1598, 1579 (Aryl C=C), 1458, 1320, 12651, 1179, 1106, 1035 (C-O-C), 1019, 838, 736, 657, 632, 499, 461.
^{1}H NMR (400 MHz, DMSO) δ ppm: 10.62 (s, 1H, Ar-OH), 10.15 (s, 1H, -HCO), 7.56 (d, J = 8.4 Hz, 1H, Ar), 6.48 – 6.42 (m, 2H, Ar), 4.01 (t, J = 6.3 Hz, 2H, $-O\underline{CH_2}CH_2CH_2CH_2CH_2CH_3$), 1.82 – 1.64 (m, 2H, $-OCH_2\underline{CH_2}CH_2CH_2CH_2CH_3$), 1.49 – 1.34 (m, 4H, $-OCH_2CH_2\underline{CH_2CH_2}CH_2CH_3$), 1.28 (m, 2H, $-OCH_2CH_2CH_2CH_2\underline{CH_2}CH_3$), 0.92 – 0.78 (m, 3H, $-OCH_2CH_2CH_2CH_2CH_2\underline{CH_3}$).
^{13}C NMR (101 MHz, DMSO) δ ppm: 186.75, 165.22, 163.37, 129.70, 117.14, 108.48, 99.48, 68.05, 31.01, 28.45, 25.23, 22.10, 13.85.

2-Heptoxy-4-hydroxybenzaldehyde (S3.7)
Yield: 5.69 g, 52%. RF: 0.20 (20:80 EtOAc:Hex)
M.P. = 86 °C Brown crystal
IR cm^{-1}: 3034 (-OH), 2923 ($-CH_2$), 2854 ($-CH_2$), 2724, 1636 (RC(=O)H), 1568 (Aryl C=C), 1458, 1396, 1324, 1280, 1245, 1182, 1112, 1019 (C-O-C), 824, 786, 659, 585, 524, 502, 460, 428.
^{1}H NMR (400 MHz, DMSO) δ ppm: 10.60 (s, 1H, Ar-OH), 10.15 (s, 1H, -HCO), 7.56 (d, J = 8.4 Hz, 1H, Ar), 6.51 – 6.41 (m, 2H, Ar), 4.02 (t, J = 6.3 Hz, 2H, $-O\underline{CH_2}CH_2CH_2CH_2CH_2CH_2CH_3$), 1.74 (hept, J = 6.3 Hz, 2H, $-OCH_2\underline{CH_2}CH_2CH_2CH_2CH_2CH_3$), 1.48 – 1.20 (m, 8H, $-OCH_2CH_2\underline{CH_2}\underline{CH_2}\underline{CH_2}\underline{CH_2}CH_3$), 0.92 – 0.79 (m, 3H, $-OCH_2CH_2CH_2CH_2CH_2CH_2\underline{CH_3}$).
^{13}C NMR (101 MHz, DMSO) δ ppm: 186.79, 165.13, 163.32, 129.75, 117.08, 108.46, 99.52, 68.04, 31.21, 28.39, 25.46, 22.04, 18.56, 13.93.

(3-Alkoxy-4-formylphenyl) 4-methoxybenzoates (**S4**)

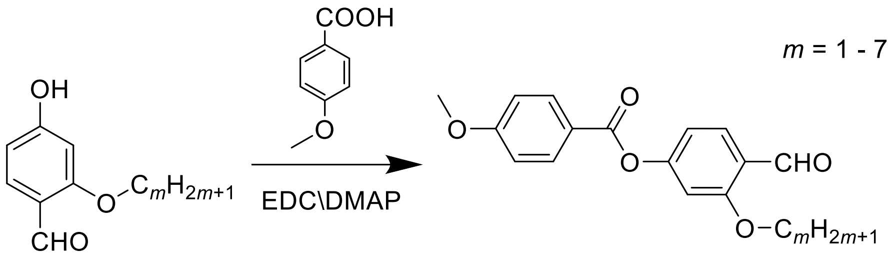

Scheme 5: Synthesis of the (3-alkoxy-4-formylphenyl) 4-methoxybenzoate intermediates.

Under inert conditions, a mixture of the required **S3** (1 eq) and 4-methoxybenzoic acid (1.5 eq) in dichloromethane (60 mL) was cooled with an ice bath. To this, dissolved 1-ethyl-3-(3-dimethylaminopropyl)carbodiimide , EDC, or *N,N'*-Dicyclohexylcarbodiimide, DCC, (1.5 eq) in 10 mL DCM was added and allowed to react at 0 °C for 30-40 min. A catalytic amount of solid 4-dimethylaminopyridine (DMAP) was added to the solution and left to react overnight slowly warming up to room temperature. The quantities of reagents used in the reaction are listed in Table SI3. The reaction mixture was then quenched by the addition of distilled water (40 mL) and washed with water and brine. The organic layer was dried with $MgSO_4$, the drying agent was filtered, and the solvent removed *in vacuo*. The product was then purified by hot recrystallization in ethanol.

Table SI3: Quantities of reagents used in the synthesis of (3-alkoxy-4-formylphenyl) 4-methoxybenzoate intermediates, **S4**.*m*.

Product name	**S3**.*m*	4-Methoxybenzoic acid	Coupling agent
S4.1	3.30 g, 2.17×10^{-2} mol	3.00 g, 1.97×10^{-2} mol	DCC: 5.28 g, 2.56×10^{-3} mol
S4.2	0.300 g, 1.8×10^{-3} mol	0.329 g, 2.16×10^{-3} mol	EDC: 0.517 g, 2.70×10^{-3} mol
S4.3	0.320 g, 1.75×10^{-3} mol	0.320 g, 2.10×10^{-3} mol	EDC: 0.203 g, 2.63×10^{-3} mol
S4.4	0.600 g, 3.09×10^{-3} mol	0.705 g, 4.63×10^{-3} mol	EDC: 0.887 g, 4.63×10^{-3} mol
S4.5	2.00 g, 9.60×10^{-3} mol	2.19 g, 1.44×10^{-3} mol	EDC: 2.76 g, 1.44×10^{-3} mol
S4.6	1.50 g, 6.75×10^{-3} mol	1.54 g, 1.01×10^{-2} mol	EDC: 1.94 g, 1.01×10^{-2} mol
S4.7	1.50 g, 6.35×10^{-3} mol	1.49 g, 9.52×10^{-3} mol	EDC: 1.83 g, 9.52×10^{-3} mol

(3-Methoxy-4-formylphenyl) 4-methoxybenzoate (S4.1)
Yield: 4.54 g, 81%. RF: 0.54 (40:60 EtOAc:Pet)
M.P. = 145 °C White crystal
IR cm^{-1}: 2844 (CH_3), 1735 (COOR), 1678 (RC(=O)H), 1602 (Aryl C=C), 1511, 1401, 1249, 1194, 1054 (C-O-C), 1024, 1006, 946, 878, 841, 821, 807, 757, 686, 666, 509, 501, 463, 436, 405.
^{1}H NMR (400 MHz, DMSO) δ ppm: 10.40 (s, 1H, -HCO), 8.12 (d, J = 8.9 Hz, 2H, Ar), 7.87 (d, J = 8.5 Hz, 1H, Ar), 6.97 (d, J = 8.9 Hz, 2H, Ar), 6.88 (m, 2H, Ar), 3.91 (s, 3H, $-O\underline{CH_3}$), 3.88 (s, 3H, $-O\underline{CH_3}$)

^{13}C NMR (101 MHz, DMSO) δ ppm: 188.63, 164.24, 164.13, 162.84, 157.24, 132.42, 129.82, 122.55, 121.10, 114.36, 114.00, 105.82, 55.92, 55.57.
Data consistent with reported literature.[1]

(3-Ethoxy-4-formylphenyl) 4-methoxybenzoate (S4.2)
Yield: 0.472 g, 87%. RF: 0.28 (20:80 EtOAc:Hex)
M.P. = 170 °C White crystal
IR cm^{-1}: 2983(CH_3), 2864 (-CH_2), 2793, 1738 (COOR), 1679 (RC(=O)H), 1602 (Aryl C=C), 1509. 1423, 1389, 1247, 1163, 1055 (C-O-C), 1014, 1006, 877, 843, 808, 759, 692, 668, 625, 528, 469, 408.
^{1}H NMR (400 MHz, DMSO) δ ppm: 10.34 (s, 1H, -HCO), 8.10 (d, J = 8.8 Hz, 2H, Ar), 7.77 (d, J = 8.4 Hz, 1H, Ar), 7.21 (d, J = 2.0 Hz, 1H, Ar), 7.15 (d, J = 9.0 Hz, 2H, Ar), 7.02 – 6.95 (m, 1H, Ar), 4.20 (q, J = 6.9 Hz, 2H, -O$\underline{CH_2}$$CH_3$), 3.88 (s, 3H, -O$\underline{CH_3}$), 1.39 (t, J = 6.9 Hz, 3H, -O$CH_2$$\underline{CH_3}$).
^{13}C NMR (101 MHz, DMSO) δ ppm: 188.25, 163.95, 163.61, 161.96, 156.99, 132.20, 128.95, 122.06, 120.56, 114.59, 114.35, 107.76, 64.65, 55.70, 14.33.
Data consistent with reported literature.[1]

(3-Propoxy-4-formylphenyl) 4-methoxybenzoate (S4.3)
Yield: 0.375 g, 64%. RF: 0.33 (20:80 EtOAc:Hex)
M.P. = 116 °C White crystal
IR cm^{-1}: 2974 (CH_3), 2906, 2868 (-CH_2), 2766, 1736 (COOR), 1680 (RC(=O)H), 1600 (Aryl C=C), 1577, 1509, 1476, 1390, 1247, 1161, 1053 (C-O-C), 997, 877, 847, 761, 693, 668, 624, 535, 405.
^{1}H NMR (400 MHz, DMSO) δ ppm: 10.35 (d, J = 0.8 Hz, 1H. -HCO), 8.09 (d, J = 8.8 Hz, 2H, Ar), 7.77 (d, J = 8.5 Hz, 1H, Ar), 7.22 (d, J = 2.0 Hz, 1H, Ar), 7.14 (d, J = 9.1 Hz, 2H, Ar), 6.98 (dd, J = 8.4, 1.5 Hz, 1H, Ar), 4.09 (t, J = 6.4 Hz, 2H, -O$\underline{CH_2}$$CH_2$$CH_3$), 3.88 (s, 3H, -O$\underline{CH_3}$), 1.79 (h, J = 7.0 Hz, 2H, -O$CH_2$$\underline{CH_2}$$CH_3$), 1.01 (t, J = 7.4 Hz, 3H, -O$CH_2$$CH_2$$\underline{CH_3}$).
^{13}C NMR (101 MHz, DMSO) δ ppm: 188.18, 163.95, 163.61, 162.11, 157.00, 132.20, 128.99, 122.12, 120.56, 114.59, 114.35, 107.79, 70.26, 55.71, 21.81, 10.33.
Data consistent with reported literature.[1]

(3-Butoxy-4-formylphenyl) 4-methoxybenzoate (S4.4)
Yield: 0.757 g, 74%. RF: 0.40 (20:80 EtOAc:Hex)
M.P. = 83 °C White crystal
IR cm^{-1}: 2999, 2957 (CH_3), 2862 (-CH_2), 1736 (COOR), 1684 (RC(=O)H), 1601 (Aryl C=C), 1509, 1430, 1389, 1250, 1160, 1054 (C-O-C), 1017, 991, 881, 846, 760, 624, 512, 404.
^{1}H NMR (400 MHz, DMSO) δ ppm: 10.34 (s, 1H, -HCO), 8.09 (d, J = 8.9 Hz, 2H, Ar), 7.77 (d, J = 8.4 Hz, 1H, Ar), 7.23 (d, J = 2.0 Hz, 1H, Ar), 7.14 (t, J = 8.8 Hz, 2H, Ar), 6.99 (dd, J = 8.6, 1.5 Hz, 1H, Ar), 4.13 (t, J = 6.4 Hz, 2H, -O$\underline{CH_2}$$CH_2$$CH_2$$CH_3$), 3.88 (s, 3H, -O$\underline{CH_3}$), 1.77 (q, J = 6.7 Hz, 2H, -O$CH_2$$\underline{CH_2}$$CH_2$$CH_3$), 1.47 (h, J = 7.4 Hz, 2H, -O$CH_2$$CH_2$$\underline{CH_2}$$CH_3$), 0.94 (t, J = 7.3 Hz, 3H, -O$CH_2$$CH_2$$CH_2$$\underline{CH_3}$).
^{13}C NMR (101 MHz, DMSO) δ ppm: 188.20, 163.96, 163.63, 162.14, 157.02, 132.21, 128.99, 122.12, 120.57, 114.60, 114.37, 107.80, 68.58, 55.72, 30.43, 18.69, 13.67.

(3-Pentoxy-4-formylphenyl) 4-methoxybenzoate (S4.5)
Yield: 1.35 g, 41%. RF: 0.42 (20:80 EtOAc:Hex)
M.P. = 77 °C Pink crystal
IR cm^{-1}: 2951 (CH_3), 2859 (-CH_2), 1736 (COOR), 1682 (RC(=O)H), 1600 (Aryl C=C), 1509, 1430, 1388, 1274, 1250, 1160, 1056 (C-O-C), 1017, 995, 882, 848, 870, 760, 692, 623, 590, 512, 465.
^{1}H NMR (400 MHz, DMSO) δ ppm: 10.34 (s, 1H, -HCO), 8.09 (d, J = 8.1 Hz, 2H, Ar), 7.77 (d, J = 8.5 Hz, 1H, Ar), 7.23 (d, J = 2.0 Hz, 1H, Ar), 7.14 (d, 2H, Ar), 6.99 (dd, J = 8.5, 2.0 Hz, 1H, Ar), 4.13 (t, J = 6.4 Hz, 2H, -O$\underline{CH_2}$$CH_2$$CH_2$$CH_2$$CH_3$), 3.88 (s, 3H, -O$\underline{CH_3}$), 1.79 (q, J = 6.7 Hz, 2H, -O$CH_2$$\underline{CH_2}$$CH_2$$CH_2$$CH_3$), 1.49 – 1.29 (m, 4H, -O$CH_2$$CH_2$$\underline{CH_2}$$\underline{CH_2}$$CH_3$), 0.90 (t, J = 7.1 Hz, 3H, -O$CH_2$$CH_2$$CH_2$$CH_2$$\underline{CH_3}$).
^{13}C NMR (101 MHz, DMSO) δ ppm: 188.17, 163.95, 163.61, 162.12, 157.01, 132.20, 128.96, 122.10, 120.56, 114.59, 114.36, 107.79, 68.84, 55.71, 28.04, 27.60, 21.81, 13.90.

(3-Hexoxy-4-formylphenyl) 4-methoxybenzoate (S4.6)
Yield: 1.28 g, 45%. RF: 0.42 (20:80 EtOAc:Hex)
M.P. = 78 °C White crystal
IR cm^{-1}: 2950 (CH_3), 2937, 2855 ($-CH_2$), 1736 (COOR), 1682 (RC(=O)H), 1601 (Aryl C=C), 1509, 1430, 1388, 1248, 1159, 1099, 1055 (C-O-C), 1016, 997, 881, 843, 760, 692, 662, 609, 512, 466.
^{1}H NMR (400 MHz, DMSO) δ ppm: 10.34 (d, J = 0.7 Hz, 1H, -HCO), 8.09 (d, J = 8.6 Hz, 2H, Ar), 7.77 (d, J = 8.5 Hz, 1H, Ar), 7.22 (d, J = 2.0 Hz, 1H, Ar), 7.15 (d, J = 8.6 Hz, 2H, Ar), 6.99 (dd, J = 8.4, 2.0 Hz, 1H, Ar), 4.13 (t, J = 6.4 Hz, 2H, $-O\underline{CH_2}CH_2CH_2CH_2CH_2CH_3$), 3.88 (s, 3H, $-O\underline{CH_3}$), 1.78 (q, J = 6.5 Hz, 2H, $-OCH_2\underline{CH_2}CH_2CH_2CH_2CH_3$), 1.50 – 1.37 (m, 2H, $-OCH_2CH_2\underline{CH_2}CH_2CH_2CH_3$), 1.38 – 1.24 (m, 4H, $-OCH_2CH_2CH_2\underline{CH_2}\underline{CH_2}CH_3$), 0.87 (t, J = 7.1 Hz, 3H, $-OCH_2CH_2CH_2CH_2CH_2\underline{CH_3}$).
^{13}C NMR (101 MHz, DMSO) δ ppm: 188.15, 163.95, 163.61, 162.12, 157.01, 132.19, 128.96, 122.10, 120.56, 114.58, 114.35, 107.79, 68.84, 55.70, 30.88, 28.29, 25.06, 22.03, 13.88.

(3-Heptoxy-4-formylphenyl) 4-methoxybenzoate (S4.7)
Yield: 1.50 g, 63%. RF: 0.44 (20:80 EtOAc:Hex)
M.P. = 75 °C White crystal
IR cm^{-1}: 2956 (CH_3), 2854 ($-CH_2$), 1737 (COOR), 1682 (RC(=O)H), 1601 (Aryl C=C), 1587, 1510, 1387, 1249 (C-O-C), 1160, 1055, 996, 848, 760, 692, 623, 513, 466, 405.
^{1}H NMR (400 MHz, DMSO) δ ppm: 10.34 (d, J = 0.8 Hz, 1H, -HCO), 8.09 (d, J = 9.1 Hz, 2H, Ar), 7.77 (d, J = 8.4 Hz, 1H, Ar), 7.22 (d, J = 2.0 Hz, 1H, Ar), 7.14 (d, J = 9.0 Hz, 2H, Ar), 6.98 (dd, J = 8.4, 2.0 Hz, 1H, Ar), 4.12 (t, J = 6.4 Hz, 2H, $-O\underline{CH_2}CH_2CH_2CH_2CH_2CH_2CH_3$), 3.88 (s, 3H, $-O\underline{CH_3}$), 1.83 – 1.72 (m, 2H, $-OCH_2\underline{CH_2}CH_2CH_2CH_2CH_2CH_3$), 1.44 (q, J = 6.7 Hz, 2H, $-OCH_2CH_2\underline{CH_2}CH_2CH_2CH_2CH_3$), 1.38 – 1.22 (m, 6H, $-OCH_2CH_2CH_2\underline{CH_2}\underline{CH_2}\underline{CH_2}CH_3$), 0.86 (t, J = 6.6 Hz, 3H, $-OCH_2CH_2CH_2CH_2CH_2CH_2\underline{CH_3}$).
^{13}C NMR (101 MHz, DMSO) δ ppm: 188.15, 163.95, 163.61, 162.12, 157.01, 132.19, 128.96, 122.10, 120.56, 114.58, 114.35, 107.79, 68.84, 55.70, 31.20, 28.34, 28.33, 25.37, 22.04, 13.94.

2-Alkoxy-4-(4-methoxybenzoyl)oxybenzoic acids (**S5**)

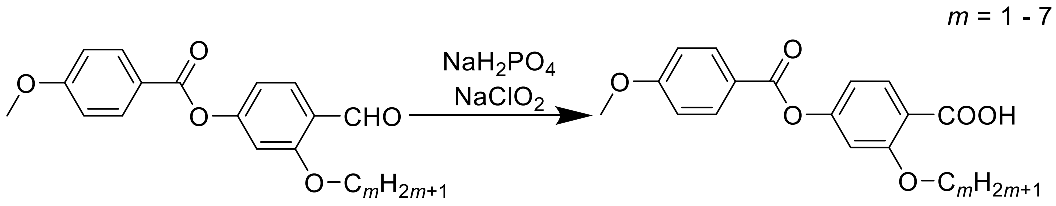

Scheme 6: Synthesis of the 2-alkoxy-4-(4-methoxybenzoyl)oxybenzoic acid intermediates.

To a mixture of **S4** (1 eq) and resorcinol (1.5 eq) in DMSO (30 mL), a solution of $H_2NaO_4P.H_2O$ (4 eq) and $ClNaO_2$ (3.5 eq) in water was slowly added dropwise. The quantities of reagents used in the reaction are listed in Table SI4. The reaction mixture was allowed to react overnight and then diluted with water (150 mL). The pH was adjusted to 8 with $NaHCO_3$ saturated and left for 1 h, then acidified with 1 M HCl solution to pH 4 until precipitation was observed. The precipitated solid was filtered and recrystallized in hot ethanol to obtain the pure product.

Table SI4: Quantities of reagents used in the synthesis of 2-alkoxy-4-(4-methoxybenzoyl)oxybenzoic acid intermediates, **S5**.*m*.

Product name	**S4**.*m*	$H_2NaO_4P.H_2O$	$ClNaO_2$	Resorcinol
S5.1	4.30 g, 1.50×10^{-2} mol	7.24 g, 5.25×10^{-2} mol	5.43 g, 6.00×10^{-2} mol	2.48 g. 2.25×10^{-2} mol
S5.2	0.750 g, 2.49×10^{-3} mol	1.38 g, 9.99×10^{-3} mol	0.788 g, 8.72×10^{-3} mol	-
S5.3	0.600 g, 1.90×10^{-3} mol	1.05 g, 7.63×10^{-3} mol	0.601 g, 6.65×10^{-3} mol	-
S5.4	0.560 g, 1.70×10^{-3} mol	0.941 g, 6.82×10^{-3} mol	0.538 g, 5.95×10^{-3} mol	-
S5.5	1.20 g, 3.50×10^{-3} mol	2.49 g, 1.39×10^{-2} mol	1.11 g, 1.22×10^{-2} mol	0.578 g, 5.25×10^{-3} mol
S5.6	1.00 g, 2.80×10^{-3} mol	1.99 g, 1.12×10^{-2} mol	0.886 g, 9.80×10^{-2} mol	0.463 g, 4.21×10^{-3} mol
S5.7	1.40 g, 3.78×10^{-3} mol	2.69 g, 1.51×10^{-2} mol	1.20 g, 1.32×10^{-2} mol	0.624 g, 5.67×10^{-3} mol

2-Ethoxy-4-(4-methoxybenzoyl)oxybenzoic acid (S5.1)

Yield: 3.95 g, 87.1%. RF: 0 (20:80 EtOAc:Hex)
M.P. = 204 °C White crystal
IR cm^{-1}: 3028 (COOH), 2987 (CH_3), 2905 ($-CH_2$), 2850, 1738 (COOR), 1669 (COOH), 1606 (Aryl C=C), 1511, 1450, 1250 (C-O-C), 1168, 1059, 1019, 878, 845, 760, 692, 660, 612, 468, 465.
3012 (COOH), 1723 (COOR), 1700, 1667 (COOH), 1606 (Aryl C=C), 1579, 1517, 1501, 1461, 1435, 1424, 1401, 1309, 1247 (C-O-C), 1189, 1164, 1024, 876, 847, 760, 692, 659, 626, 457, 439.
^{1}H NMR (400 MHz, DMSO) δ ppm: 12.65 (s, 1H, -COOH), 8.09 (d, J = 8.7 Hz, 2H, Ar), 7.74 (d, J = 8.4 Hz, 1H, Ar), 7.13 (d, J = 8.7 Hz, 2H, Ar), 7.09 (d, J = 2.0 Hz, 1H, Ar), 6.91 (dd, J = 8.4 Hz, 2.0 Hz, 1H, Ar), 3.88 (s, 3H, -O$\underline{CH_3}$), 3.82 (s, 3H, -O$\underline{CH_3}$).
^{13}C NMR (101 MHz, DMSO) δ ppm: 167.05, 164.32, 164.27, 159.90, 154.86, 132.58, 132.31, 121.17, 119.04, 114.77, 114.09, 107.30, 56.58, 56.14.
Data consistent with reported literature.[1]

2-Ethoxy-4-(4-methoxybenzoyl)oxybenzoic acid (S5.2)

Yield: 0.549 g, 63%. RF: 0 (20:80 EtOAc:Hex)
M.P. = 130 °C White crystal
IR cm^{-1}: 3028 (COOH), 2987 (CH_3), 2905 ($-CH_2$), 2850, 1738 (COOR), 1669 (COOH), 1606 (Aryl C=C), 1511, 1450, 1250 (C-O-C), 1168, 1059, 1019, 878, 845, 760, 692, 660, 612, 468, 465.
^{1}H NMR (400 MHz, DMSO) δ ppm: 12.58 (s, 1H, -COOH), 8.09 (d, J = 9.1 Hz, 2H, Ar), 7.70 (d, J = 8.4 Hz, 1H, Ar), 7.13 (d, J = 9.3 Hz, 2H, Ar), 7.06 (d, J = 2.1 Hz, 1H, Ar), 6.88 (dd, J = 8.4, 2.1 Hz, 1H, Ar), 4.08 (q, J = 7.0 Hz, 2H, -O$\underline{CH_2}CH_3$), 3.87 (s, 3H, -O$\underline{CH_3}$), 1.32 (t, J = 6.9 Hz, 3H, -$OCH_2\underline{CH_3}$).
^{13}C NMR (101 MHz, DMSO) δ ppm: 166.81, 163.85, 158.69, 154.18, 132.13, 131.67, 120.76, 114.31, 113.52, 107.65, 70.01, 55.69, 21.93, 10.37.
Data consistent with reported literature.[1]

2-Propoxy-4-(4-methoxybenzoyl)oxybenzoic acid (S5.3)

Yield: 0.477 g, 76%. RF: 0 (20:80 EtOAc:Hex)
M.P. = 130 °C White crystal
IR cm^{-1}: 3080 (COOH), 2976 (CH_3), 2904 ($-CH_2$), 2873, 1729 (COOR), 1666 (COOH), 1604 (Aryl C=C), 1578, 1447, 1395, 1247 (C-O-C), 1167, 1055, 1001, 946, 880, 843, 761, 692, 660, 607, 585, 513, 428.
^{1}H NMR (400 MHz, DMSO) δ ppm: 12.58 (s, 1H, -COOH), 8.09 (d, J = 8.2 Hz, 2H, Ar), 7.71 (d, J = 8.4 Hz, 1H, Ar), 7.13 (d, J = 8.9 Hz, 2H, Ar), 7.07 (d, J = 2.1 Hz, 1H, Ar), 6.89 (dd, J = 8.4, 2.1 Hz,

1H, Ar), 3.98 (t, J = 6.4 Hz, 2H, -O$\underline{CH_2}CH_2CH_3$), 3.88 (s, 3H, -O$\underline{CH_3}$), 1.73 (hept, J = 6.7 Hz, 2H, -O$CH_2\underline{CH_2}CH_3$), 0.99 (t, J = 7.4 Hz, 3H, -O$CH_2CH_2\underline{CH_3}$).
^{13}C NMR (101 MHz, DMSO) δ ppm: 166.80, 163.86, 158.72, 154.22, 132.14, 131.71, 120.76, 119.04, 114.32, 113.53, 107.66, 70.03, 55.70, 21.93, 10.38.
Data consistent with reported literature.[1]

2-Butoxy-4-(4-methoxybenzoyl)oxybenzoic acid (S5.4)

Yield: 0.514 g, 87%. RF: 0 (20:80 EtOAc:Hex)
M.P. = 123 °C White crystal
IR cm^{-1}: 3031 (COOH), 2985 (CH_3), 2859 (-CH_2), 2902, 2876, 1735 (COOR), 1676 (COOH), 1605 (Aryl C=C), 1580, 1513, 1450, 1252 (C-O-C), 1169, 1058, 1019, 885, 846, 760, 661, 608, 467.
^{1}H NMR (400 MHz, DMSO) δ ppm: 12.57 (s, 1H, -COOH), 8.09 (d, J = 8.9 Hz, 2H, Ar), 7.70 (d, J = 8.4 Hz, 1H, Ar), 7.13 (d, J = 8.6 Hz, 2H, Ar), 7.08 (d, J = 2.1 Hz, 1H, Ar), 6.88 (dd, J = 8.4, 2.1 Hz, 1H, Ar), 4.02 (t, J = 6.4 Hz, 2H, -O$\underline{CH_2}CH_2CH_2CH_3$), 3.88 (s, 3H, -O$\underline{CH_3}$), 1.69 (p, J = 8.1 Hz, 2H, -O$CH_2\underline{CH_2}CH_2CH_3$), 1.45 (h, J = 7.8 Hz, 2H, -O$CH_2CH_2\underline{CH_2}CH_3$), 0.92 (t, J = 7.4 Hz, 3H, -O$CH_2CH_2CH_2\underline{CH_3}$).
^{13}C NMR (101 MHz, DMSO) δ ppm: 166.79, 163.84, 158.68, 154.18, 132.12, 131.63, 120.75, 119.10, 114.31, 113.51, 107.64, 68.26, 55.68, 30.58, 18.60, 13.64.

2-Pentoxy-4-(4-methoxybenzoyl)oxybenzoic acid (S5.5)

Yield: 0.750 g, 60%. RF: 0.04 (20:80 EtOAc:Hex)
M.P. = 85 °C White crystal
IR cm^{-1}: 3030 (COOH), 2958 (CH_3), 2938, 2855 (-CH_2), 1734 (COOR), 1667 (COOH), 1603 (Aryl C=C), 1578, 1512, 1247 (C-O-C), 1162, 1057, 1023 998, 883, 844, 760, 691, 760, 691, 660, 607, 511, 438.
^{1}H NMR (400 MHz, DMSO) δ ppm: 12.45 (s, 1H, -COOH), 8.09 (d, J = 8.7 Hz, 2H, Ar), 7.70 (d, J = 8.4 Hz, 1H, Ar), 7.13 (d, J = 8.5 Hz, 2H, Ar), 7.07 (d, J = 2.1 Hz, 1H, Ar), 6.88 (dd, J = 8.5, 2.1 Hz, 1H, Ar), 4.01 (t, J = 6.4 Hz, 2H, -O$\underline{CH_2}CH_2CH_2CH_2CH_3$), 3.88 (s, 3H, -O$\underline{CH_3}$), 1.71 (p, J = 6.7 Hz, 2H, -O$CH_2\underline{CH_2}CH_2CH_2CH_3$), 1.47 – 1.26 (m, 4H, -O$CH_2CH_2\underline{CH_2CH_2}CH_3$), 0.88 (t, J = 7.1 Hz, 3H, -O$CH_2CH_2CH_2CH_2\underline{CH_3}$).
^{13}C NMR (101 MHz, DMSO) δ ppm: 166.83, 163.85, 158.67, 154.15, 132.13, 131.62, 120.76, 119.21, 114.31, 113.52, 107.66, 68.57, 55.69, 28.17, 27.55, 21.77, 13.93.

2-Hexoxy-4-(4-methoxybenzoyl)oxybenzoic acid (S5.6)

Yield: 0.960 g, 92%. RF: 0.04 (20:80 EtOAc:Hex)
M.P. = 86 °C White crystal
IR cm^{-1}: 3030 (COOH), 2956 (CH_3), 2854 (-CH_2), 1736 (COOR), 1682 (COOH), 1601 (Aryl C=C), 1511, 1389, 1247 (C-O-C), 1161, 1055, 997, 882, 845, 760, 692, 624, 511.
^{1}H NMR (400 MHz, DMSO) δ ppm: 12.46 (s, 1H, -COOH), 8.09 (d, J = 8.8 Hz, 2H, Ar), 7.70 (d, J = 8.4 Hz, 1H, Ar), 7.13 (d, J = 9.0 Hz, 2H, Ar), 7.07 (d, J = 2.1 Hz, 1H, Ar), 6.88 (dd, J = 8.4, 2.1 Hz, 1H, Ar), 4.01 (t, J = 6.4 Hz, 2H, -O$\underline{CH_2}CH_2CH_2CH_2CH_2CH_3$), 3.88 (s, 3H, -O$\underline{CH_3}$), 1.70 (p, J = 6.6 Hz, 2H, -O$CH_2\underline{CH_2}CH_2CH_2CH_2CH_3$), 1.48 – 1.37 (m, 2H, -O$CH_2CH_2\underline{CH_2}CH_2CH_2CH_3$), 1.35 – 1.25 (m, 4H, -O$CH_2CH_2CH_2\underline{CH_2CH_2}CH_3$), 0.87 (t, J = 7.3 Hz, 3H, -O$CH_2CH_2CH_2CH_2CH_2\underline{CH_3}$).
^{13}C NMR (101 MHz, DMSO) δ ppm: 166.87, 163.85, 158.66, 154.12, 132.12, 131.60, 120.76, 119.28, 114.30, 113.49, 107.64, 68.55, 56.03, 30.86, 28.44, 24.99, 22.06, 18.56, 13.88.

2-Heptoxy-4-(4-methoxybenzoyl)oxybenzoic acid (S5.7)

Yield: 1.29 g, 88%. RF: 0.04 (20:80 EtOAc:Hex)
M.P. = 65 °C White crystal
IR cm^{-1}: 3030 (COOH), 2930 (CH_3), 2850 (-CH_2), 1734 (COOR), 1663 (COOH), 1604 (Aryl C=C), 1510, 1394, 1246 (C-O-C), 1168, 1053, 1005, 886, 845, 760, 691, 660, 606, 509, 445.
^{1}H NMR (400 MHz, DMSO) δ ppm: 12.56 (s, 1H, -COOH), 8.09 (d, J = 8.8 Hz, 2H, Ar), 7.71 (d, J = 8.4 Hz, 1H, Ar), 7.13 (d, J = 9.7 Hz, 2H, Ar), 7.07 (d, J = 2.1 Hz, 1H, Ar), 6.88 (dd, J = 8.4, 2.1 Hz, 1H, Ar), 4.01 (t, J = 6.4 Hz, 2H, -O$\underline{CH_2}CH_2CH_2CH_2CH_2CH_2CH_3$), 3.88 (s, 3H, -O$\underline{CH_3}$), 1.70 (p, J =

6.2 Hz, 2H, $-OCH_2\underline{CH_2}CH_2CH_2CH_2CH_2CH_3$), 1.48 – 1.37 (m, 2H, $-OCH_2CH_2\underline{CH_2}CH_2CH_2CH_2CH_3$), 1.36 – 1.19 (m, 6H, $-OCH_2CH_2CH_2\underline{CH_2CH_2CH_2}CH_3$), 0.91 – 0.77 (m, 2H, $-OCH_2CH_2CH_2CH_2CH_2CH_2\underline{CH_3}$).
^{13}C NMR (101 MHz, DMSO) δ ppm: 166.78, 163.84, 158.69, 154.18, 132.11, 131.64, 120.75, 119.10, 114.30, 113.50, 107.64, 68.54, 55.67, 31.25, 28.48, 28.32, 25.28, 22.02, 13.96.

(3-Fluoro[a]-4-nitrophenyl) 2-alkoxy-4-(4-methoxybenzoyl)oxybenzoates (NT3.*m* – NT3F.*m*[a])

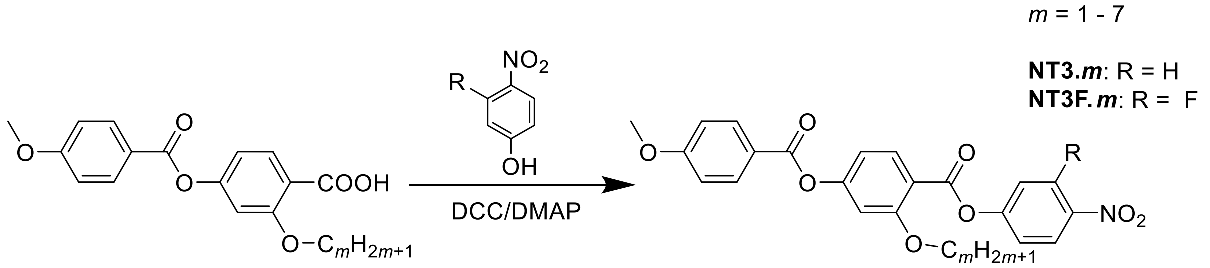

Scheme 7: Synthesis of the (4-nitrophenyl) 2-alkoxy-4-(4-methoxybenzoyl)oxybenzoates.

Under inert conditions, a mixture of **S5** (1.1 eq) and either 3-fluoro-4-nitrophenol (1 eq) or 4-nitrophenol (1 eq) were dissolved in dry dichloromethane (10 mL). To this, *N,N'*-dicyclohexylcarbodiimide (1.25 eq) in 2 mL DCM was added and allowed to react for 1h. A catalytic amount of solid 4-dimethylaminopyridine (DMAP) was added to the solution and allowed to react overnight. The quantities of reagents used in the reaction are listed in Table SI5 and SI6. The reaction mixture was then quenched by filtration of the precipitated DCU and then purified by flash column chromatography with DCM:EtOAc (97:3). The product was then purified by hot recrystallization in ethanol.

Table SI5: Quantities of reagents used in the synthesis of (4-nitrophenyl) 2-alkoxy-4-(4-methoxybenzoyl)oxybenzoate, NT3.*m*.

Product name	**S5**.*m*	4-Nitrophenol	DCC
NT3.1	0.300 g, 9.92×10^{-4} mol	0.207 g, 1.49×10^{-3} mol	0.307 g, 1.49×10^{-3} mol
NT3.2	0.300 g, 9.48×10^{-4} mol	0.119 g, 8.62×10^{-4} mol	0.222 g, 1.08×10^{-3} mol
NT3.3	0.200 g, 6.05×10^{-4} mol	0.0766 g, 5.50×10^{-4} mol	0.141 g, 6.87×10^{-4} mol
NT3.4	0.250 g, 7.25×10^{-4} mol	0.0918 g, 6.59×10^{-4} mol	0.169 g, 8.23×10^{-4} mol
NT3.5	0.200 g, 5.58×10^{-4} mol	0.0705 g, 5.07×10^{-4} mol	0.131 g, 6.33×10^{-4} mol
NT3.6	0.300 g, 5.73×10^{-4} mol	0.0720 g, 5.20×10^{-4} mol	0.134 g, 6.51×10^{-4} mol
NT3.7	0.300 g, 7.76×10^{-4} mol	0.0980g, 7.06×10^{-4} mol	0.182 g, 8.82×10^{-4} mol

(4-Nitrophenyl) 2-methoxy-4-(4-methoxybenzoyl)oxybenzoate (NT3.1)

Yield: 0.022 g, 5.2%. RF: 0.35 (40:60 EtOAc:Hex)
T_{CrI} 192 °C T_{N_FN} (126 °C) T_{NI} (189 °C).
IR cm^{-1}: 1728 (COOR), 1717, 1614 (Aryl C=C), 1593, 1519 (NO_2), 1490, 1349, 1263, 1242 (C-O-C), 1216, 1194, 1173, 1160, 1127, 886, 863, 758, 689, 614, 457.
^{1}H NMR (400 MHz, DMSO) δ ppm: 8.35 (d, J 9.0 Hz, 2H, Ar), 8.11 (m, 3H, Ar), 7.59 (d, J = 9.0 Hz, 2H, Ar), 7.26 (d, J = 2.0 Hz, 1H, Ar), 7.15 (d, J = 8.7 Hz, 2H, Ar), 7.06 (dd, J = 8.5 Hz, 2.0 Hz, 1H, Ar), 3.90 (s, 3H, $-O\underline{CH_3}$), 3.89 (s, 3H, $-O\underline{CH_3}$).
^{13}C NMR (101 MHz, DMSO) δ ppm: 164.41, 164.13, 162.69, 161.25, 156.50, 155.99, 145.54, 133.63, 132.67, 125.78, 123.87, 121.03, 115.47, 114.82, 114.59, 107.78, 56.97, 56.18.
Data consistent with reported literature.[1]

(4-Nitrophenyl) 2-ethoxy-4-(4-methoxybenzoyl)oxybenzoate (NT3.2)

Yield: 0.033 g, 10%. RF: 0.71 (97:3 DCM:EtOAc)
T_{CrI} 164 °C T_{NI} (137 °C) T_{N_FN} (104 °C).

IR cm^{-1}: 3090 (Aryl -H), 2944 ($-CH_2$), 1915, 1738 (COOR), 1711, 1610 (Aryl C=C), 1581, 1520 (NO_2), 1490, 1428, 1352, 1289, 1240 (C-O-C), 1208, 1171, 1115, 1039, 877, 836, 757, 686, 611, 495, 413.
^{1}H NMR (400 MHz, DMSO) δ ppm: 8.36 (d, J = 8.9 Hz, 2H, Ar), 8.11 (d, J = 9.4 Hz, 2H, Ar), 8.06 (d, J = 8.5 Hz, 1H, Ar), 7.58 (d, J = 9.6 Hz, 2H, Ar), 7.23 (d, J = 2.1 Hz, 1H, Ar), 7.15 (d, J = 8.7 Hz, 2H, Ar), 7.04 (dd, J = 8.5, 2.1 Hz, 1H, Ar), 4.17 (q, J = 6.9 Hz, 2H, $-O\underline{CH_2}CH_3$), 3.89 (s, 3H), $-O\underline{CH_3}$, 1.35 (t, J = 6.9 Hz, 3H, $-OCH_2\underline{CH_3}$).

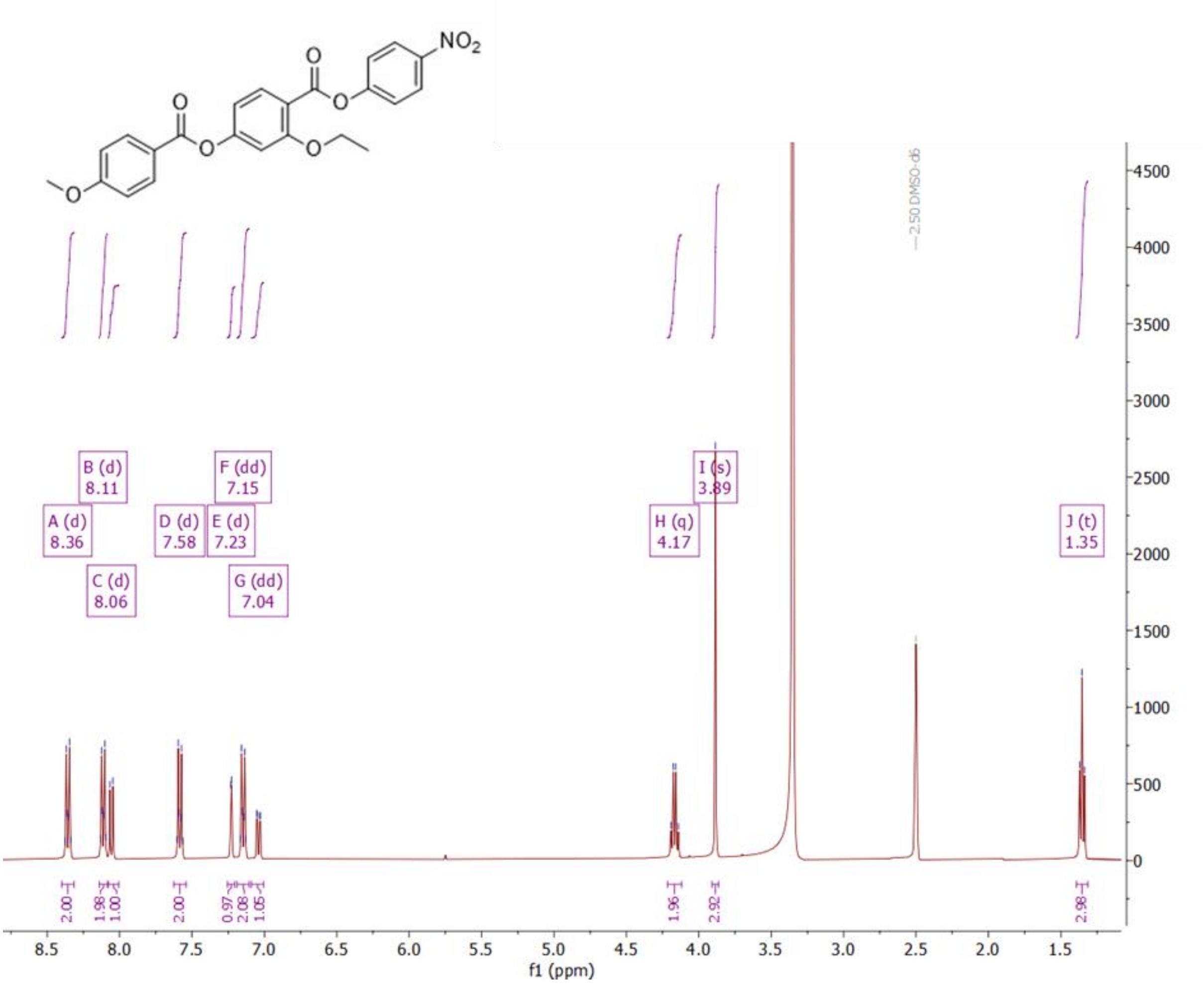

^{13}C NMR (101 MHz, DMSO) δ ppm: 163.99, 163.75, 162.52, 160.01, 155.94, 155.62, 145.10, 133.09, 132.26, 125.40, 123.36, 120.63, 115.37, 114.40, 114.11, 108.13, 64.80, 55.75, 14.45.

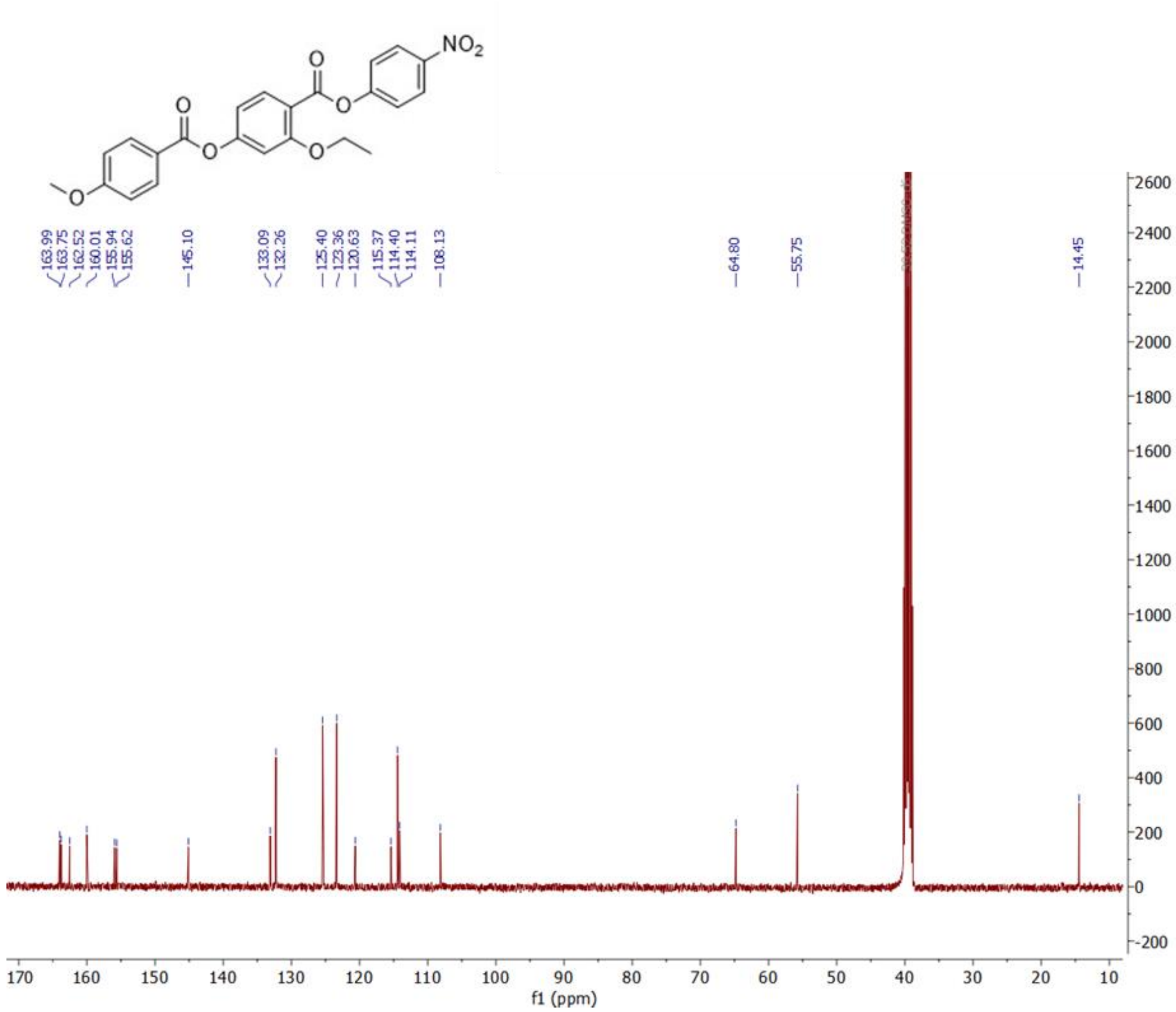

Elemental Analysis: Calculated: C 63.16%, H 4.38%, N 3.20%. Found: C 63.46%, H 4.09%, N 3.06%. Data consistent with reported literature.[1]

(4-Nitrophenyl) 2-propoxy-4-(4-methoxybenzoyl)oxybenzoate (NT3.3)

Yield: 0.131 g, 53%. RF: 0.65 (97:3 DCM:EtOAc)

T_{CrI} 130 °C T_{NI} (105 °C) T_{N_FN} (93 °C).

IR cm^{-1}: 3085 (Aryl -H), 2965 (-CH$_2$), 2844, 1738 (COOR), 1704, 1605 (Aryl C=C), 1521 (NO$_2$), 1433, 1349, 1258 (C-O-C), 1208, 1163, 1117, 1055, 1019, 881, 846, 760, 688, 670, 608, 579, 499, 416.

^{1}H NMR (400 MHz, DMSO) δ ppm: 8.36 (d, J = 9.1 Hz, 2H, Ar), 8.12 (dd, J = 7.6, 3.0 Hz, 2H, Ar), 8.06 (d, J = 8.5 Hz, 1H, Ar), 7.58 (d, J = 9.1 Hz, 2H, Ar), 7.24 (d, J = 2.1 Hz, 1H, Ar), 7.15 (dd, J = 196.3, 7.6 Hz, 2H, Ar), 7.04 (dd, J = 8.6, 2.1 Hz, 1H, Ar), 4.07 (t, J = 6.3 Hz, 2H, -O$\underline{CH_2}$CH$_2$CH$_3$), 3.89 (s, 3H, -O$\underline{CH_3}$), 1.75 (h, J = 7.0 Hz, 2H, -OCH$_2\underline{CH_2}$CH$_3$), 0.98 (t, J = 7.4 Hz, 3H, -OCH$_2$CH$_2\underline{CH_3}$).

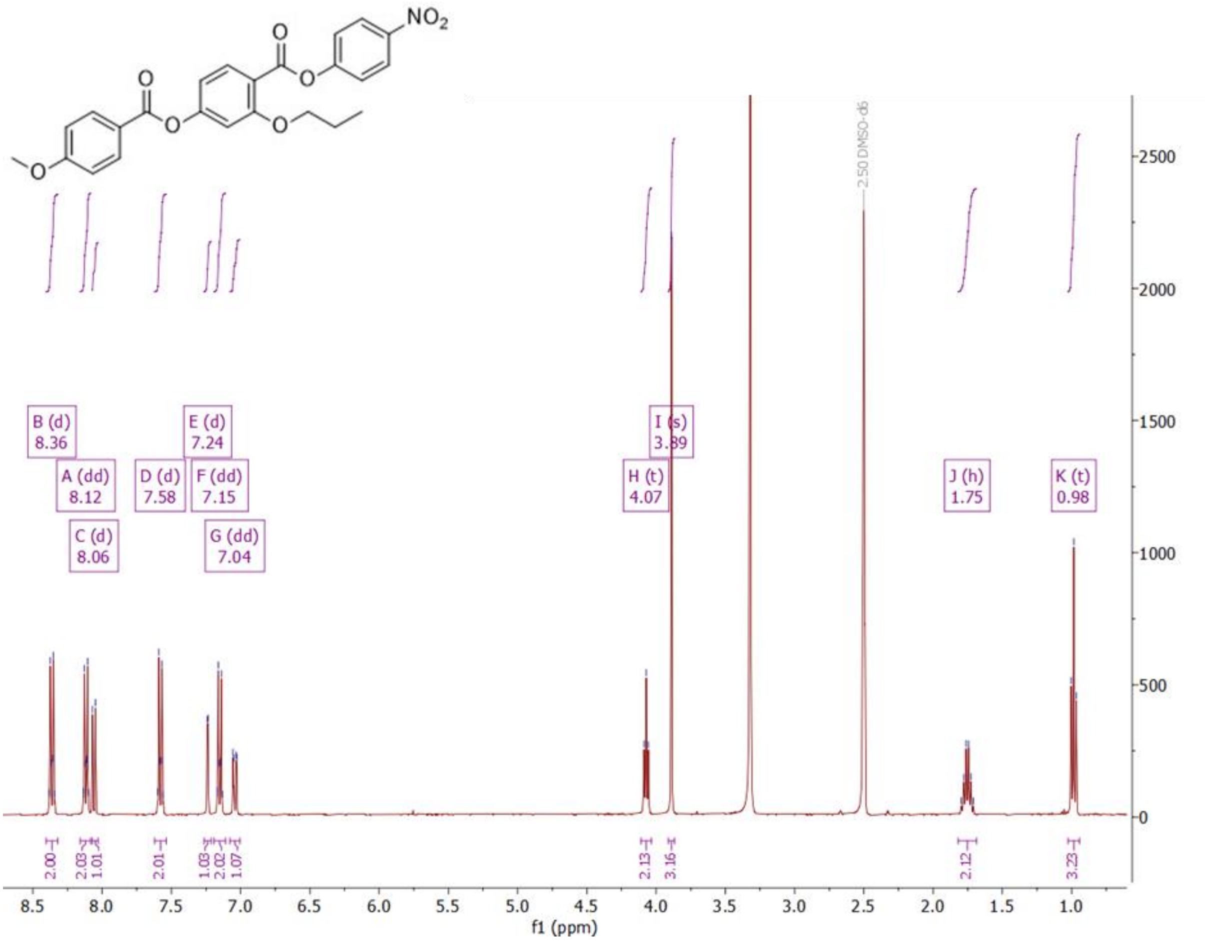

^{13}C NMR (101 MHz, DMSO) δ ppm: 163.94, 163.69, 162.55, 160.13, 155.95, 155.59, 145.06, 133.13, 132.20, 125.37, 123.29, 120.59, 115.19, 114.35, 114.02, 108.01, 70.33, 55.71, 21.90, 10.37.

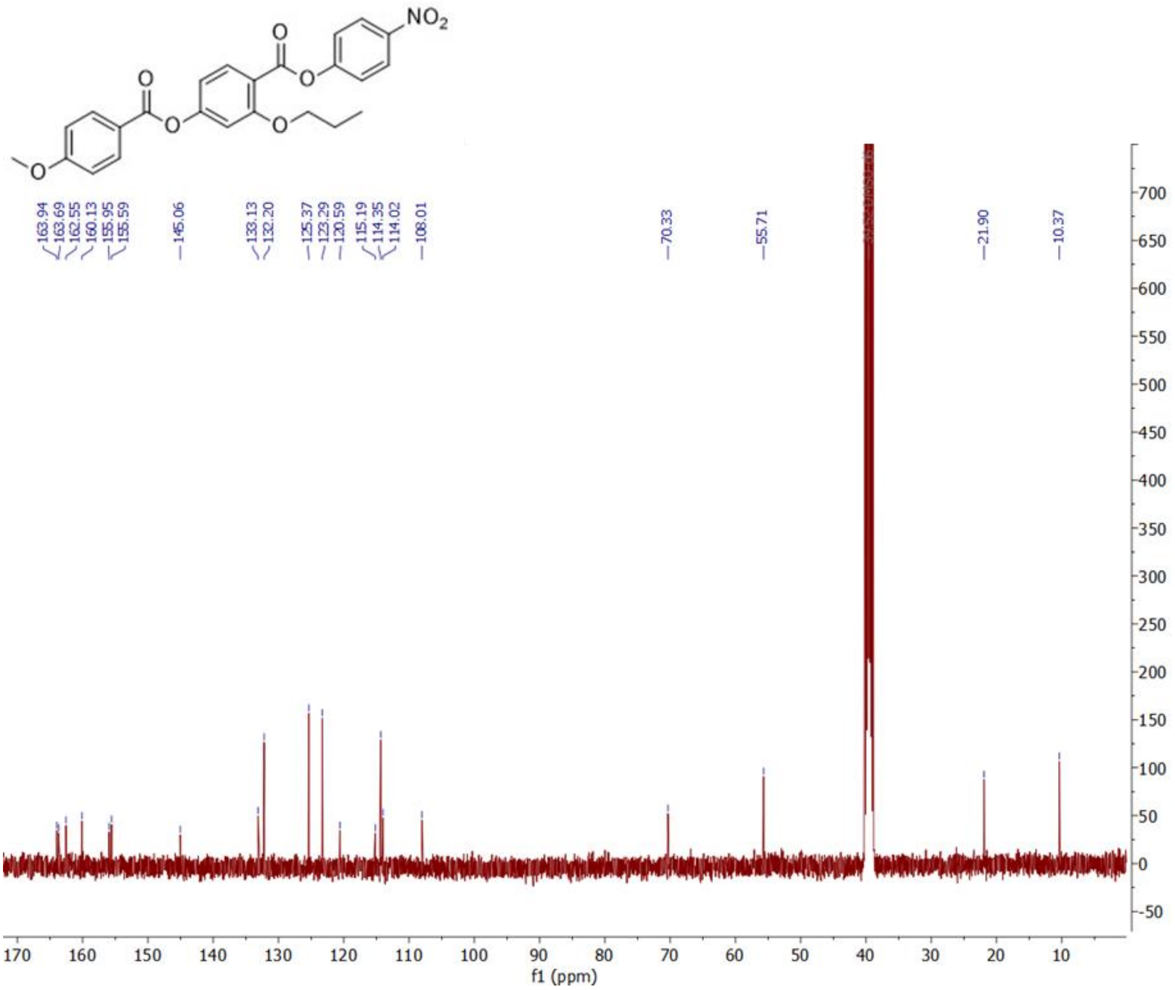

Elemental Analysis: Calculated: C 63.86%, H 4.82%, N 2.91%. Found: C 63.85%, H 4.56%, N 3.08%. Data consistent with reported literature.[1]

(4-Nitrophenyl) 2-butoxy-4-(4-methoxybenzoyl)oxybenzoate (NT3.4)

Yield: 0.175 g, 51%. RF: 0.16 (DCM)

T_{CrI} 120 °C T_{NI} (82 °C) T_{N_FN} (76 °C).

IR cm^{-1}: 3092 (Aryl -H), 2953 (-CH_2), 2873, 1750 (COOR), 1725, 1605 (Aryl C=C), 1530 (NO_2), 1432, 1350, 1256 (C-O-C), 1201, 1168, 1127, 1077, 1004, 845, 759, 742, 687, 492.

^{1}H NMR (400 MHz, DMSO) δ ppm: 8.36 (d, J = 9.2 Hz, 2H, Ar), 8.11 (d, J = 231.1 Hz, 2H, Ar), 8.06 (d, J = 8.5 Hz, 1H, Ar), 7.57 (dd, J = 484.1, 9.5 Hz, 2H, Ar), 7.24 (d, J = 2.1 Hz, 1H, Ar), 7.14 (d, J = 7.2 Hz, 2H, Ar), 7.03 (dd, J = 8.5, 2.1 Hz, 1H, Ar), 4.10 (d, J = 6.2 Hz, 2H, -O$\underline{CH_2}CH_2CH_2CH_3$), 3.88 (s, 3H, -O$\underline{CH_3}$), 1.72 (p, J = 5.6 Hz, 2H, -$OCH_2\underline{CH_2}CH_2CH_3$), 1.45 (h, J = 7.4 Hz, 2H, -$OCH_2CH_2\underline{CH_2}CH_3$), 0.89 (t, J = 6.2 Hz, 3H, -$OCH_2CH_2CH_2\underline{CH_3}$).

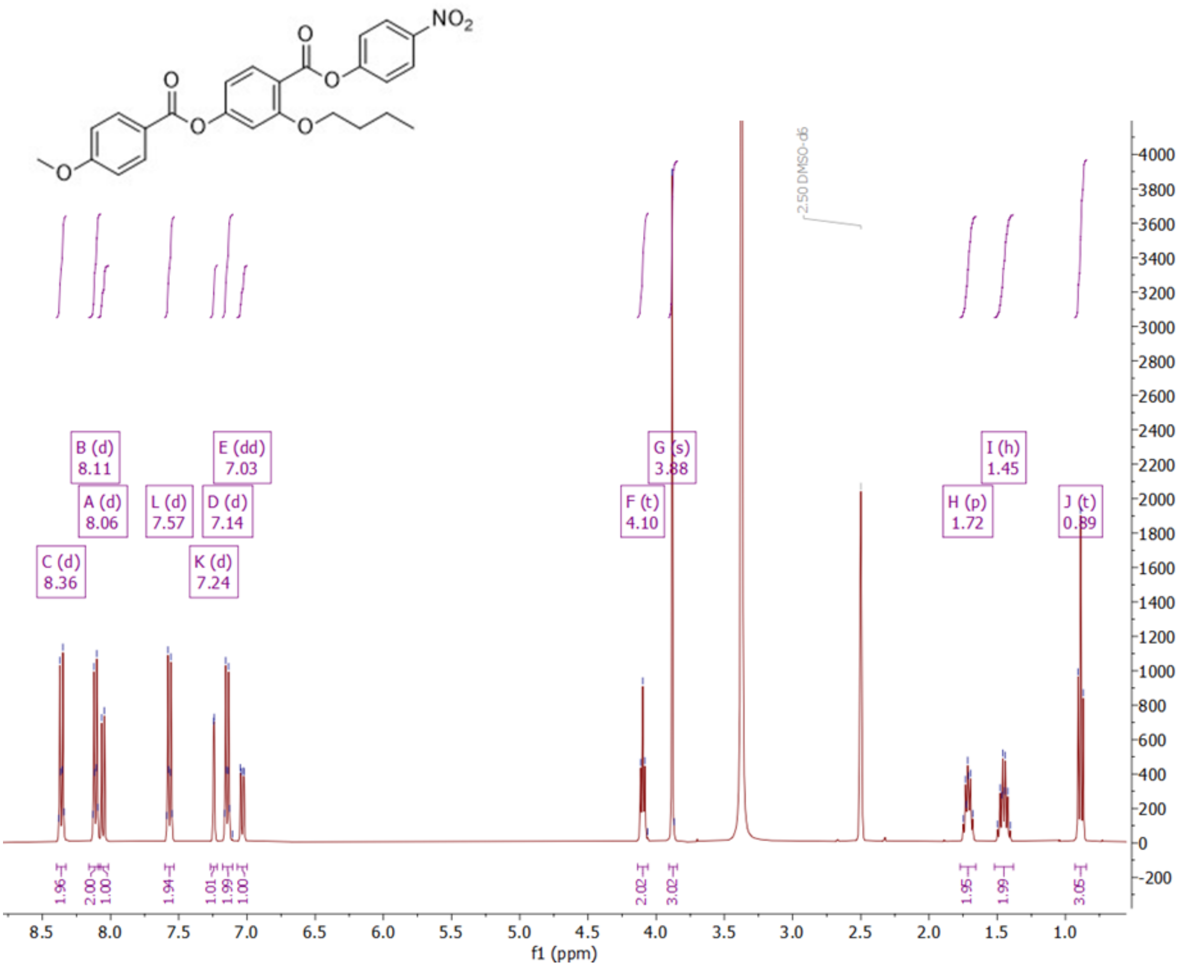

^{13}C NMR (101 MHz, DMSO) δ ppm: 163.98, 163.75, 162.56, 160.22, 156.01, 155.64, 145.09, 133.19, 132.26, 125.43, 123.34, 120.62, 115.18, 114.40, 114.07, 108.03, 68.57, 55.75, 30.55, 18.65, 13.63.

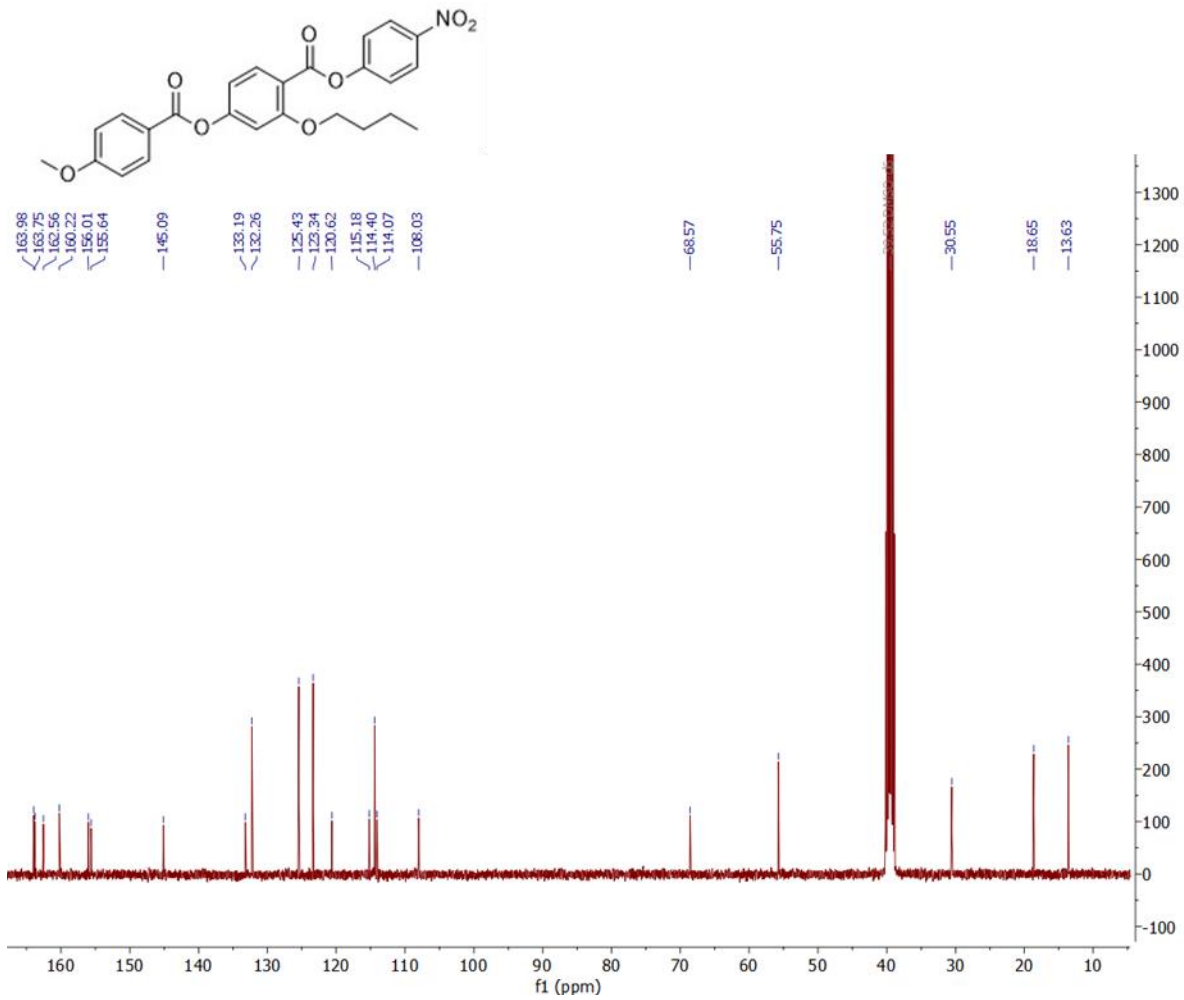

Elemental Analysis: Calculated: C 64.51%, H 4.98%, N 3.01%. Found: C 65.01%, H 4.54%, N 2.96%.

(4-Nitrophenyl) 2-pentoxy-4-(4-methoxybenzoyl)oxybenzoate (NT3.5)
Yield: 0.145 g, 54%. RF: 0.18 (DCM)
T_{CrI} 102 °C T_{NI} (66 °C) T_{N_FN} (61 °C).

IR cm^{-1}: 3088 (Aryl -H), 2953 (-CH_2), 2871, 1736 (COOR), 1713, 1603 (Aryl C=C), 1578, 1511 (NO_2), 1423, 1352, 1285, 1254 (C-O-C), 1205, 1163, 1112, 1074, 1043, 997, 881, 845, 760, 689, 612, 495, 408.

^1H NMR (400 MHz, DMSO) δ ppm: 8.36 (d, J = 8.4 Hz, 2H, Ar), 8.11 (d, J = 9.4 Hz, 2H, Ar), 8.05 (d, J = 8.6 Hz, 1H, Ar), 7.57 (d, J = 8.7 Hz, 2H, Ar), 7.24 (d, J = 1.4 Hz, 1H, Ar), 7.15 (d, J = 8.8 Hz, 2H, Ar), 7.03 (dd, J = 8.4, 1.4 Hz, 1H, Ar), 4.10 (t, J = 6.3 Hz, 2H, -O$\underline{CH_2}CH_2CH_2CH_2CH_3$), 3.89 (s, 3H, -O$\underline{CH_3}$), 1.73 (p, J = 6.5 Hz, 2H, -$OCH_2\underline{CH_2}CH_2CH_2CH_3$), 1.47 – 1.23 (m, 4H, -$OCH_2CH_2\underline{CH_2}\underline{CH_2}CH_3$), 0.82 (t, J = 7.0 Hz, 3H, -$OCH_2CH_2CH_2CH_2\underline{CH_3}$).

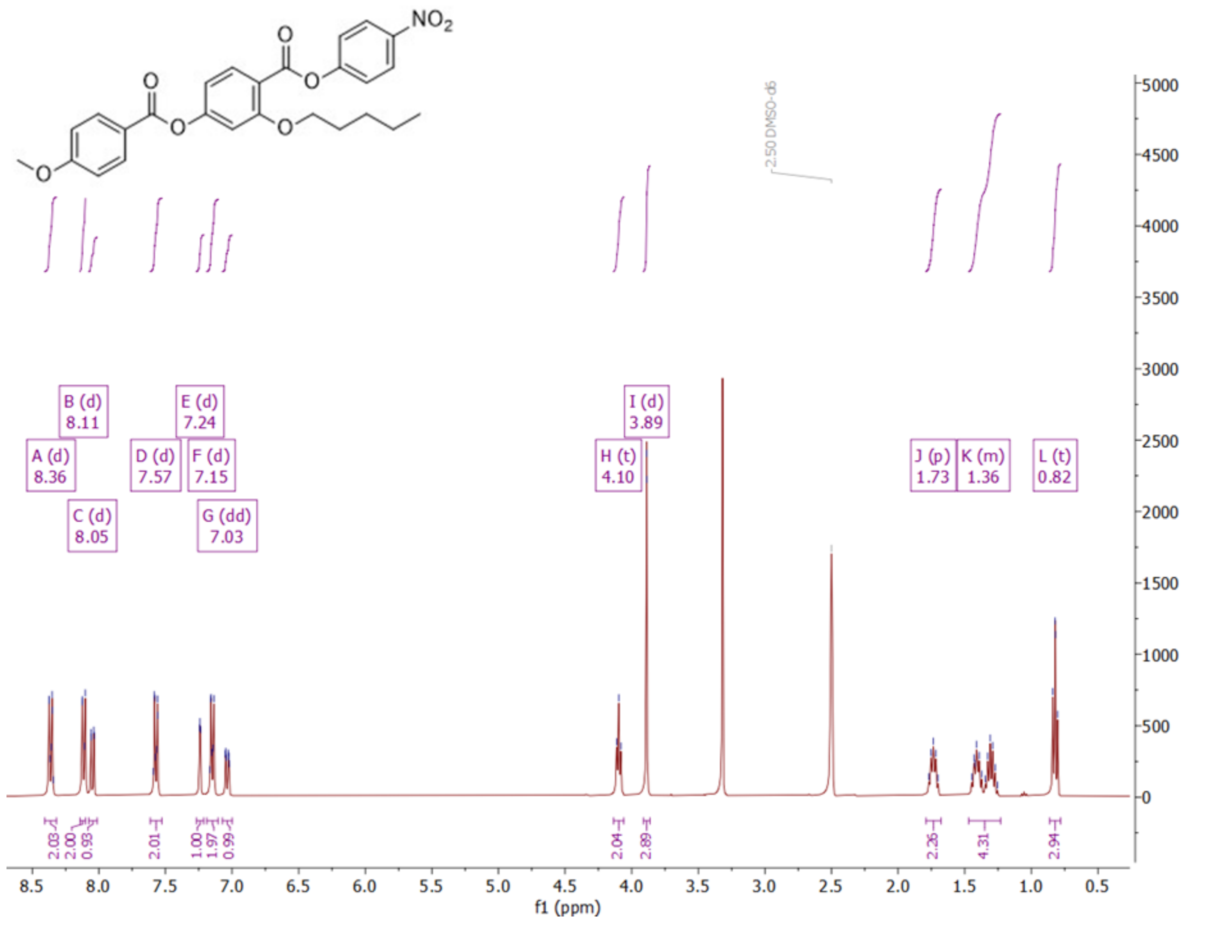

^{13}C NMR (101 MHz, DMSO) δ ppm: 163.93, 163.68, 162.59, 160.12, 155.95, 155.60, 145.04, 133.11, 132.19, 125.35, 123.25, 120.59, 115.20, 114.34, 114.01, 108.00, 68.83, 55.70, 28.13, 27.55, 21.71, 13.86.

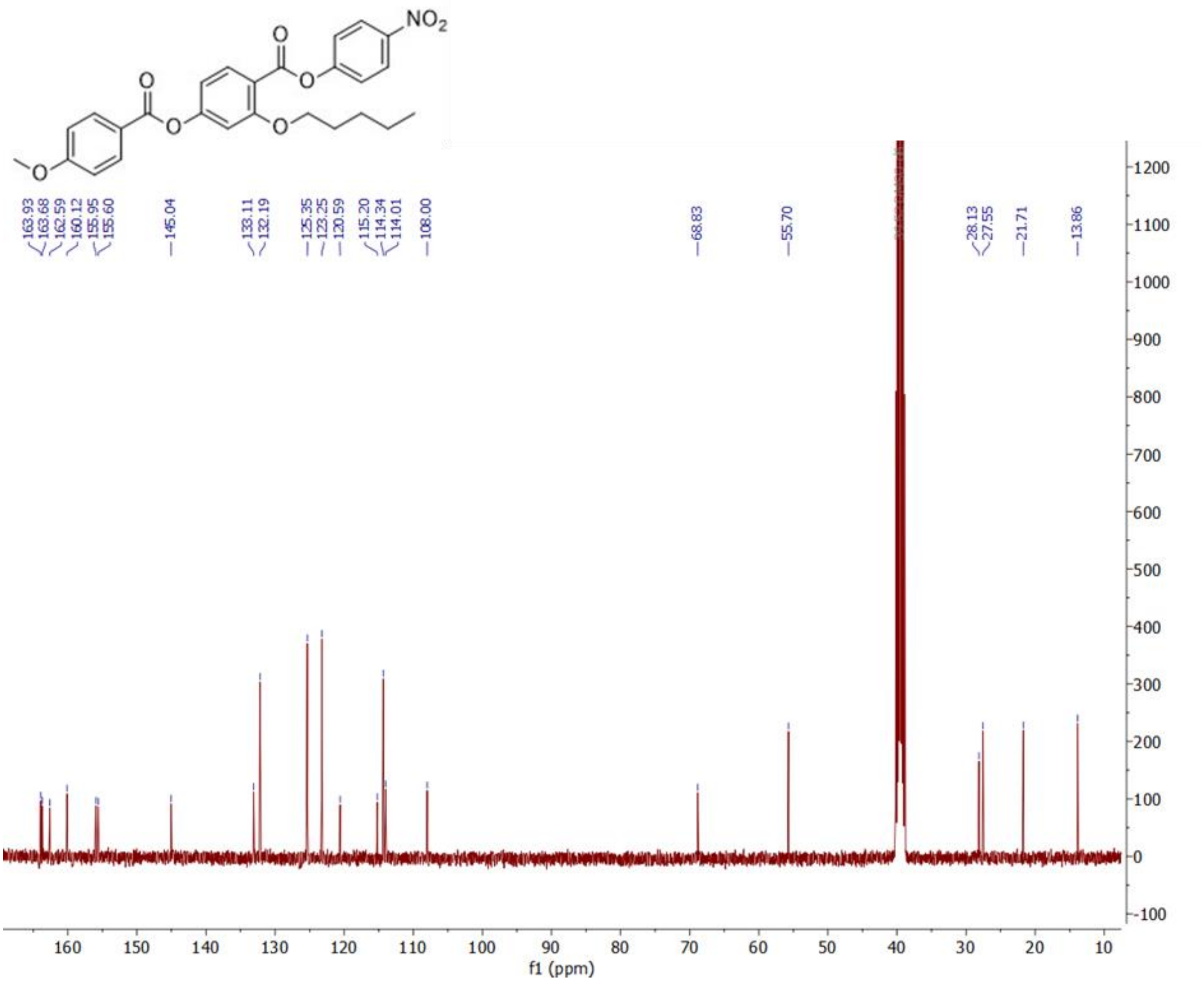

Elemental Analysis: Calculated: C 65.13%, H 5.26%, N 2.92%. Found: C 65.05%, H 5.37%, N 2.86%.

(4-Nitrophenyl) 2-hexoxy-4-(4-methoxybenzoyl)oxybenzoate (NT3.6)
Yield: 0.164 g, 58%. RF: 0.21 (DCM)
T_{CrI} 94 °C T_{NI} (57 °C) T_{N_FN} (53 °C).

IR cm^{-1}: 3070 (Aryl -H), 2952 (-CH_2), 2856, 1734 (COOR), 1711, 1605 (Aryl C=C), 1575, 1520 (NO_2), 1431, 1347, 1259 (C-O-C), 1203, 1164, 1118, 1044, 1010, 885, 843, 759, 690, 606, 500, 408.
^{1}H NMR (400 MHz, DMSO) δ ppm: 8.36 (d, J = 9.0 Hz, 2H, Ar), 8.11 (d, J = 8.7 Hz, 2H, Ar), 8.04 (d, J = 8.5 Hz, 1H, Ar), 7.57 (d, J = 8.9 Hz, 2H, Ar), 7.24 (d, J = 2.1 Hz, 1H, Ar), 7.15 (d, J = 8.7 Hz, 2H, Ar), 7.03 (dd, J = 8.5, 2.1 Hz, 1H, Ar), 4.10 (t, J = 6.3 Hz, 2H, $-O\underline{CH_2}CH_2CH_2CH_2CH_2CH_3$), 3.89 (s, 3H, $-O\underline{CH_3}$), 1.72 (p, J = 6.6 Hz, 2H, $-OCH_2\underline{CH_2}CH_2CH_2CH_2CH_3$), 1.47 – 1.36 (m, 2H, $-OCH_2CH_2\underline{CH_2}CH_2CH_2CH_3$), 1.32 – 1.15 (m, 4H, $-OCH_2CH_2CH_2\underline{CH_2}\underline{CH_2}CH_3$), 0.80 (t, J = 6.9 Hz, 3H, $-OCH_2CH_2CH_2CH_2CH_2\underline{CH_3}$).

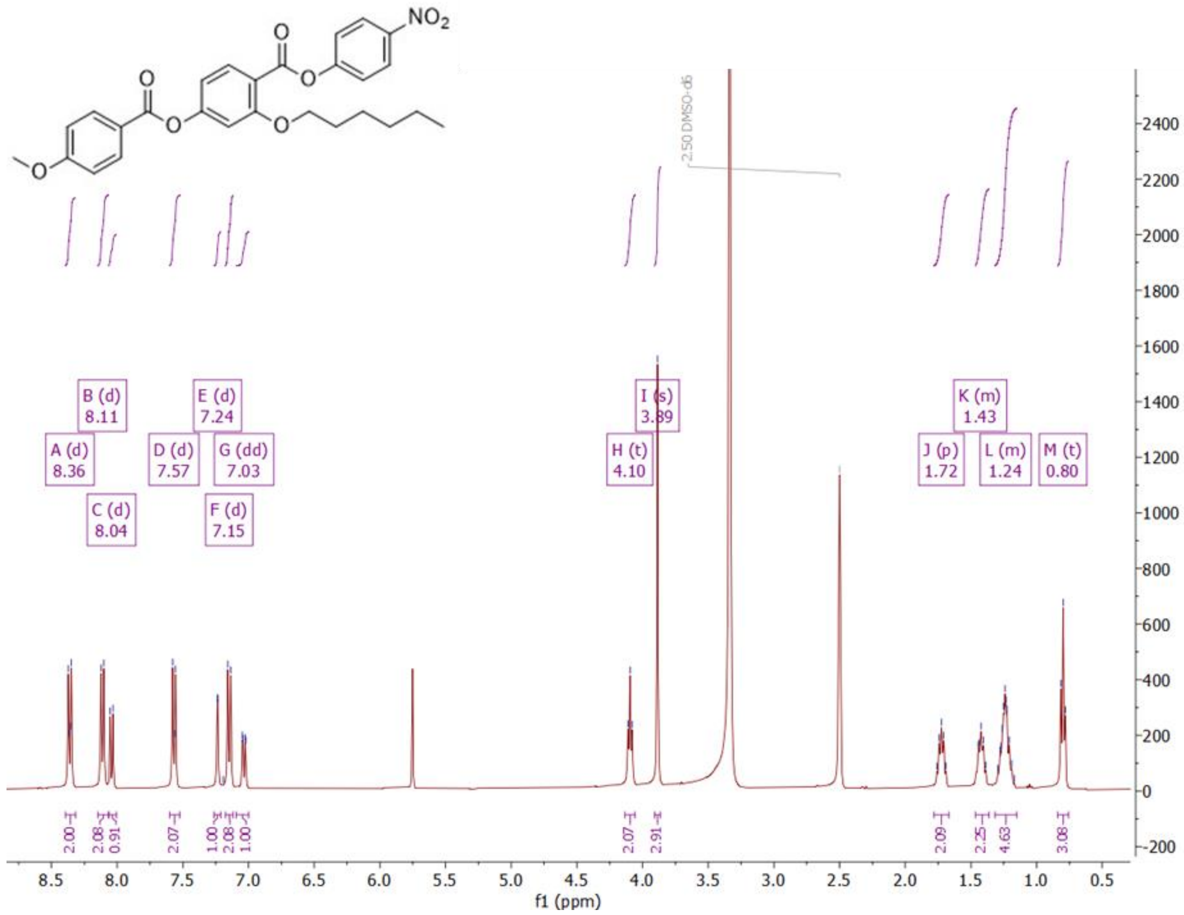

^{13}C NMR (101 MHz, DMSO) δ ppm: 163.95, 163.70, 162.65, 160.12, 155.96, 155.62, 145.05, 133.11, 132.21, 125.36, 123.25, 120.60, 115.23, 114.36, 114.02, 108.00, 68.86, 55.71, 30.83, 28.43, 25.05, 22.03, 13.82.

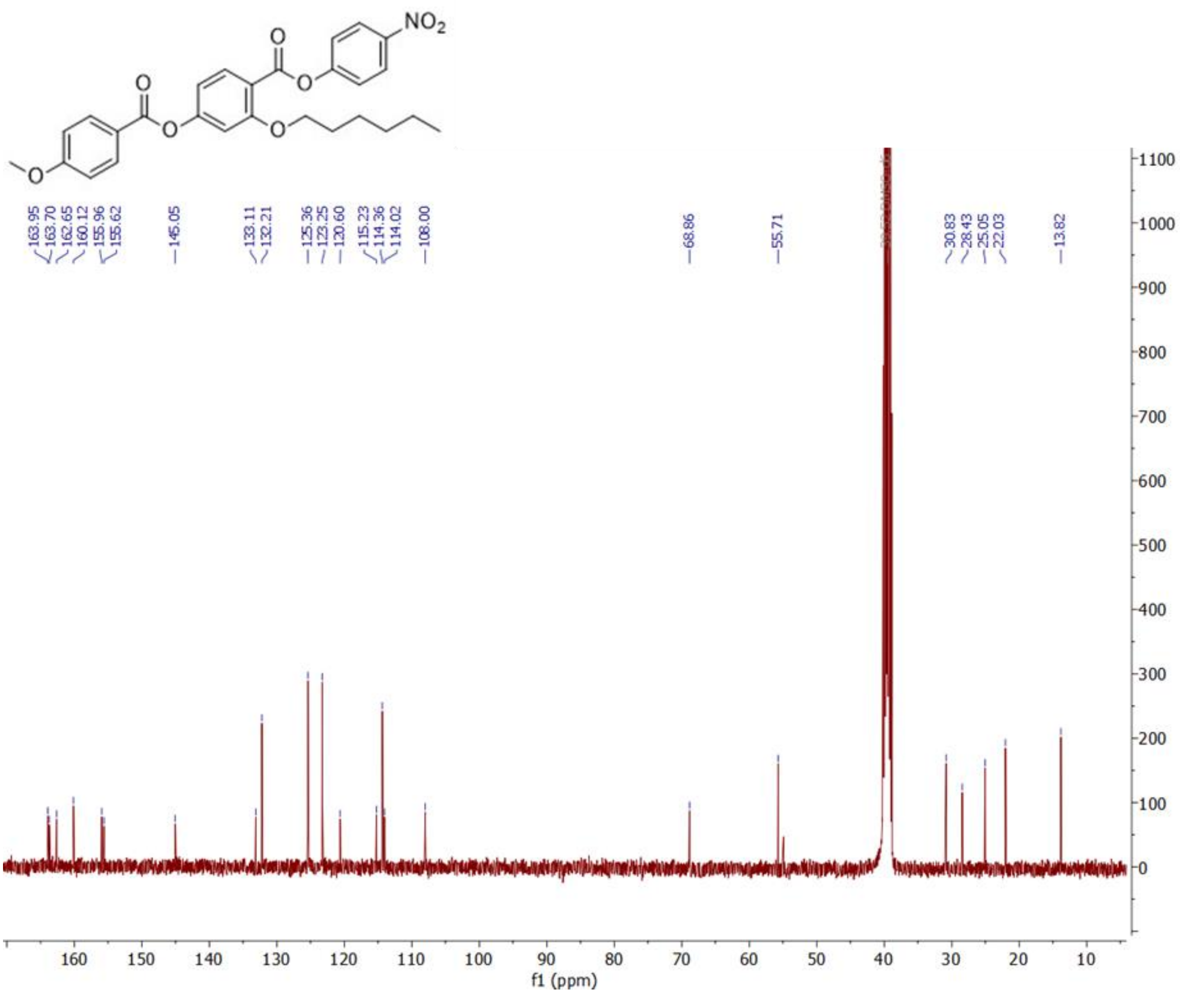

Elemental Analysis: Calculated: C 65.71%, H 5.51%, N 2.84%. Found: C 65.63%, H 5.56%, N 2.79%.

(4-Nitrophenyl) 2-heptoxy-4-(4-methoxybenzoyl)oxybenzoate (NT3.7)
Yield: 0.215 g, 52%. RF: 0.69 (DCM)
T_{CrI} 72 °C T_{NI} (54 °C) T_{N_FN} (51 °C).
IR cm^{-1}: 3066 (Aryl -H), 2927 (-CH_2), 2855, 1733 (COOR), 1710, 1604 (Aryl C=C), 1580, 1512 (NO_2), 1431, 1347, 1252 (C-O-C), 1201, 1163, 1122, 1004, 883, 843, 759, 689, 607, 499, 408.
^{1}H NMR (400 MHz, DMSO) δ ppm: 8.36 (d, J = 9.1 Hz, 2H, Ar), 8.11 (d, J = 8.9 Hz, 2H, Ar), 8.04 (d, J = 8.5 Hz, 1H, Ar), 7.57 (d, J = 8.8 Hz, 2H, Ar), 7.24 (d, J = 2.1 Hz, 1H, Ar), 7.15 (d, J = 8.8 Hz, 2H, Ar), 7.04 (dd, J = 8.6, 2.1 Hz, 1H, Ar), 4.10 (t, J = 6.2 Hz, 2H, -$O\underline{CH_2}CH_2CH_2CH_2CH_2CH_2CH_3$), 3.89 (s, 3H, -$O\underline{CH_3}$), 1.72 (p, J = 6.4 Hz, 2H, -$OCH_2\underline{CH_2}CH_2CH_2CH_2CH_2CH_3$), 1.41 (p, J = 7.2 Hz, 2H, -$OCH_2CH_2\underline{CH_2}CH_2CH_2CH_2CH_3$), 1.33 – 1.12 (m, 6H, -$OCH_2CH_2CH_2\underline{CH_2}\underline{CH_2}\underline{CH_2}CH_3$), 0.79 (t, J = 6.8 Hz, 3H, -$OCH_2CH_2CH_2CH_2CH_2CH_2\underline{CH_3}$).

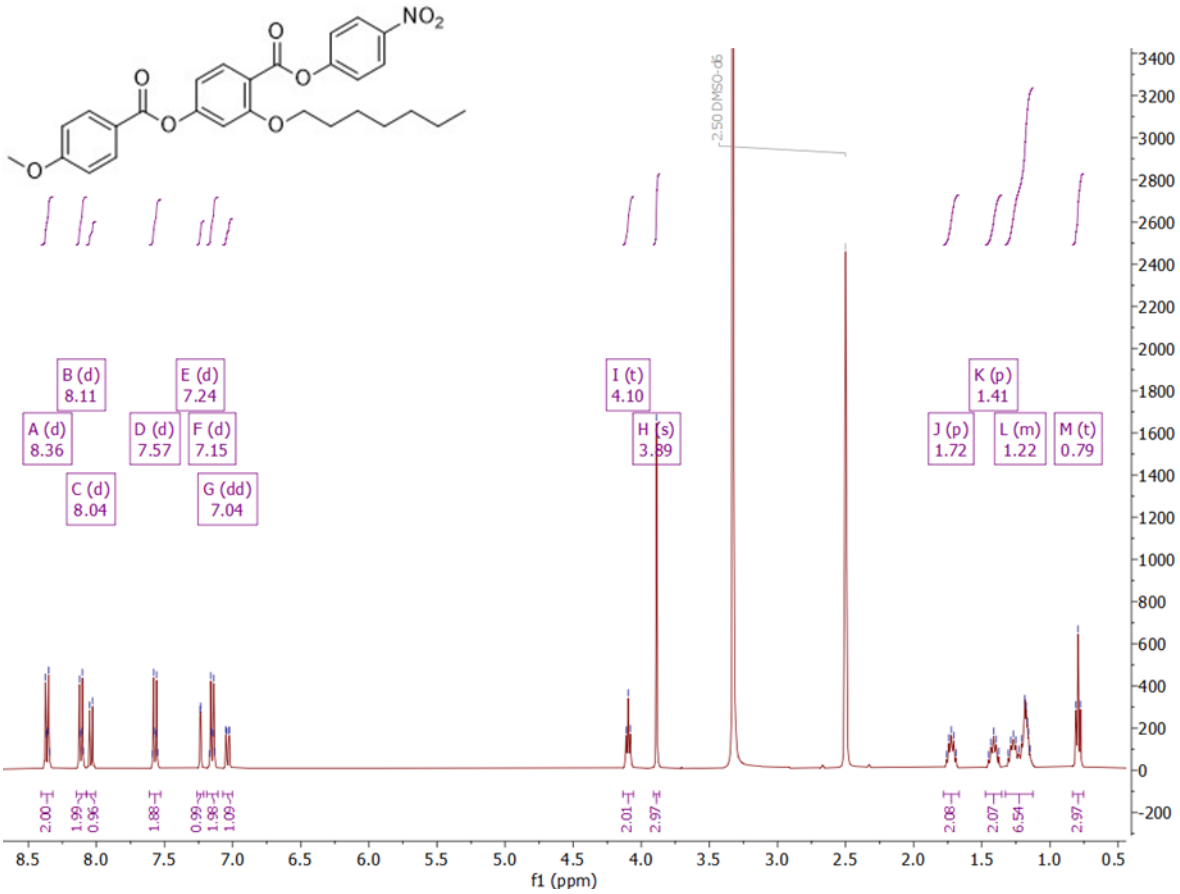

^{13}C NMR (101 MHz, DMSO) δ ppm: 163.94, 163.69, 162.69, 160.10, 155.95, 155.62, 145.03, 133.11, 132.20, 125.35, 123.23, 120.59, 115.24, 114.35, 114.03, 108.00, 68.84, 55.71, 31.21, 28.48, 28.31, 25.36, 21.98, 13.89.

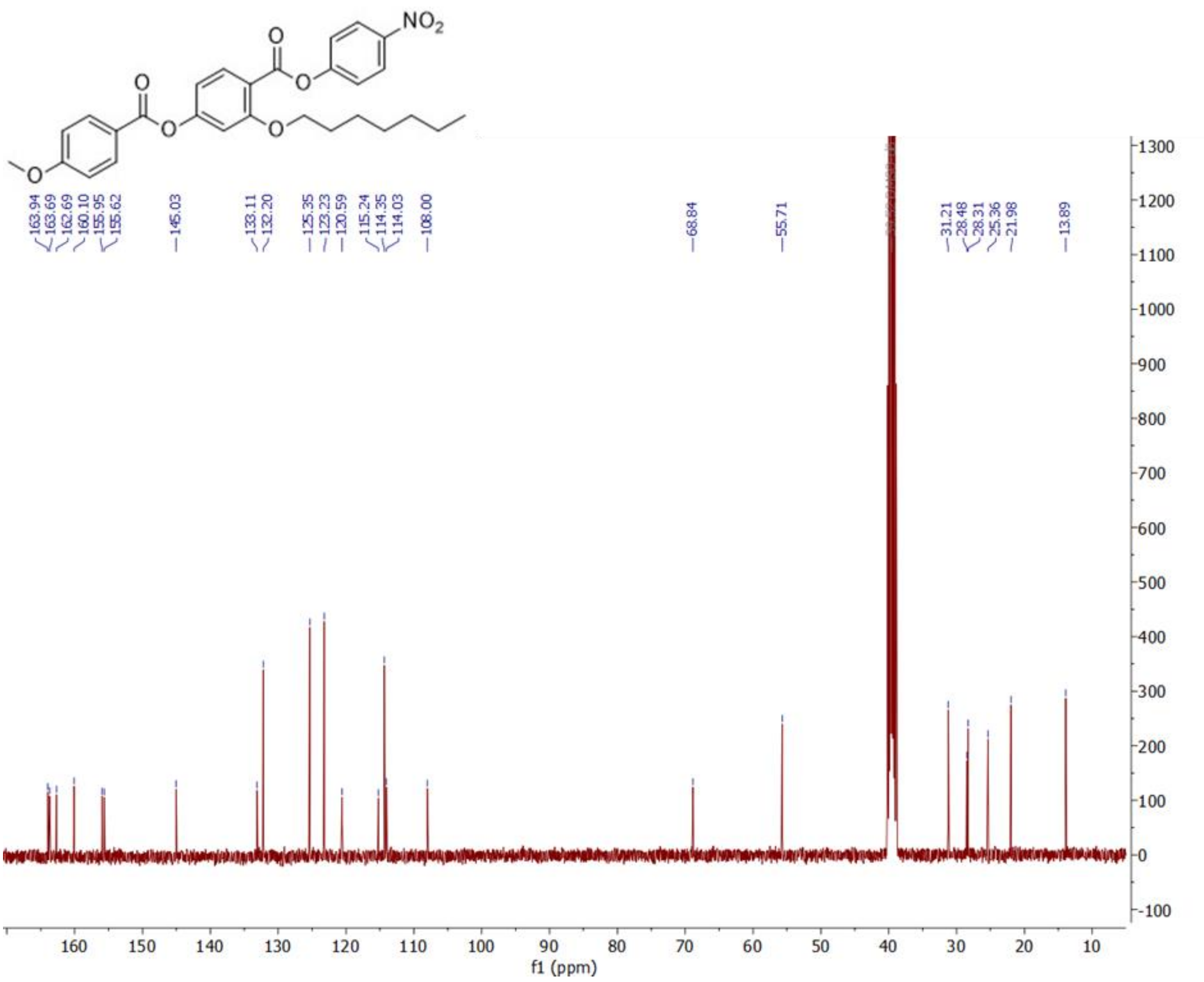

Elemental Analysis: Calculated: C 66.26%, H 5.76%, N 2.76%. Found: C 66.52%, H 5.27%, N 2.72%.

Table SI6: Quantities of reagents used in the synthesis of (3-fluoro-4-nitrophenyl) 2-alkoxy-4-(4-methoxybenzoyl)oxybenzoates, NT3F.*m*.

Product name	**S5**.*m*	3-Fluoro-4-nitrophenol	DCC
NT3F.1	0.300 g, 9.92×10^{-4} mol	0.187 g, 1.19×10^{-4} mol	0.307 g, 1.49×10^{-3} mol
NT3F.2	0.300 g, 8.85×10^{-4} mol	0.112 g, 8.04×10^{-4} mol	0.207 g, 1.01×10^{-3} mol
NT3F.3	0.200 g, 6.05×10^{-4} mol	0.0860 g, 5.50×10^{-4} mol	0.142 g, 6.87×10^{-4} mol
NT3F.4	0.150 g, 4.36×10^{-4} mol	0.0622 g, 3.95×10^{-4} mol	0.101 g, 4.93×10^{-4} mol
NT3F.5	0.200 g, 5.58×10^{-4} mol	0.0791 g, 5.07×10^{-4} mol	0.131 g, 6.33×10^{-4} mol
NT3F.6	0.300 g, 5.73×10^{-4} mol	0.0816 g, 5.20×10^{-4} mol	0.134 g, 6.51×10^{-3} mol
NT3F.7	0.300 g, 7.76×10^{-4} mol	0.110 g, 7.06×10^{-4} mol	0.182 g, 8.82×10^{-4} mol

(3-Fluoro-4-nitrophenyl) 2-ethoxy-4-(4-methoxybenzoyl)oxybenzoate (NT3F.1)

Yield: 0.285 g, 65.1%. RF: 0.31 (DCM)

T_{CrI} 180 °C T_{N_FN} (141 °C). T_{NI} (156 °C).

IR cm^{-1}: 1763 (COOR), 1723, 1716, 1604 (Aryl C=C), 1584, 1531 (NO_2), 1513, 1497, 1472, 1454, 1412, 1350, 1320, 1288, 1259 (C-O-C), 1193, 1141, 1119, 1093, 1028, 1007, 877, 760, 745, 680, 624, 545, 510, 423.

^{1}H NMR (400 MHz, DMSO) δ ppm: 8.30 (t, J = 8.9 Hz, 1H, Ar), 8.11 (m, 3H, Ar), 7.73 (dd, J = 12.1 Hz, 2.3 Hz, 1H, Ar), 7.43 (m, 1H, Ar), 7.26 (d, J = 2.0 Hz, 1H, Ar), 7.15 (d, J = 8.8 Hz, 2H, Ar), 7.07 (dd, J = 8.6 Hz, 2.0 Hz, 1H, Ar), 3.90 (s, 3H, O<u>CH</u>$_3$), 3.89 (s, 3H, O<u>CH</u>$_3$).

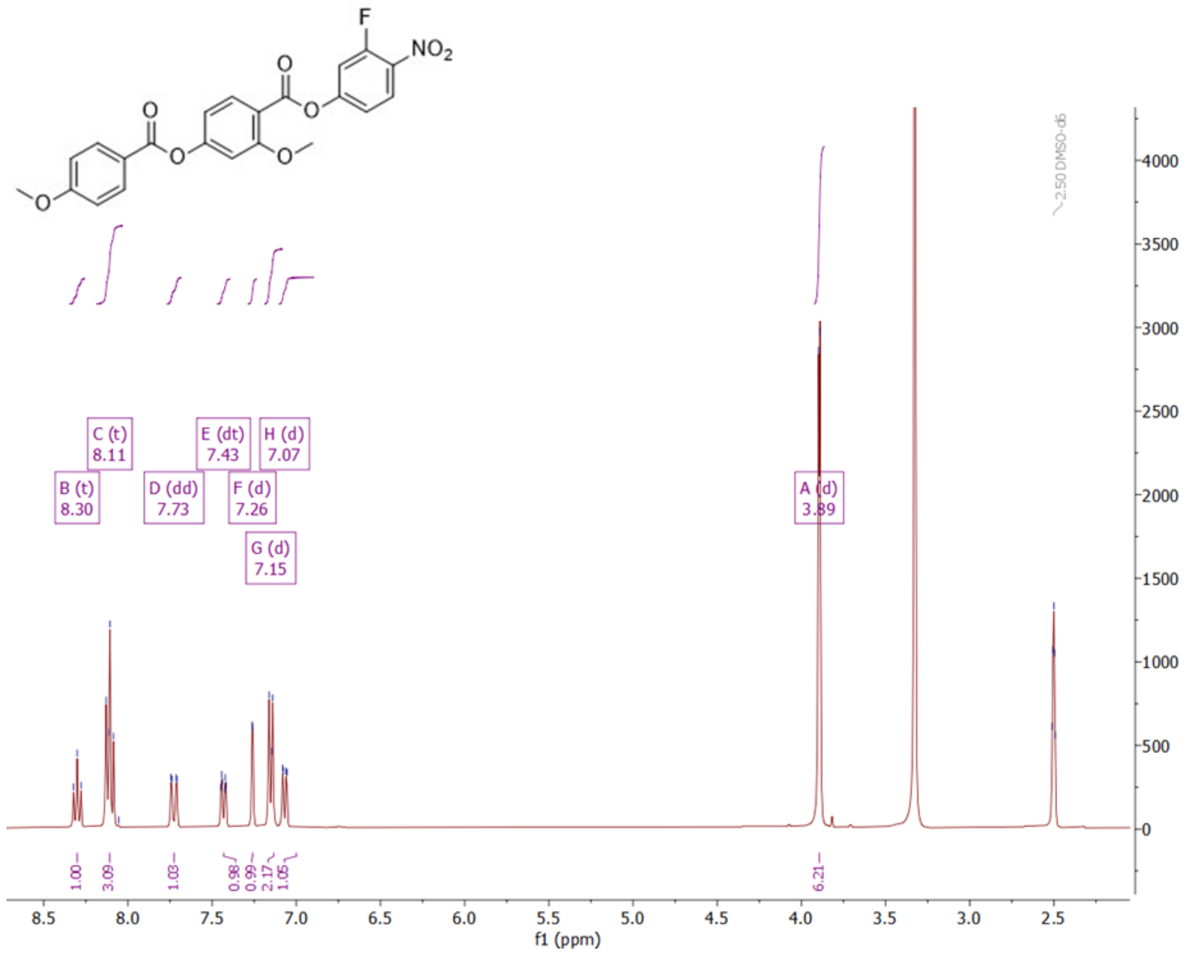

^{13}C NMR (101 MHz, DMSO) δ ppm: 164.44, 164.12, 162.16, 161.47, 156.71, 156.17 (d, *J* = 11.9 Hz), 154.45 (d, *J* = 262.2 Hz), 135.03 (d, *J* = 6.7 Hz), 133.83, 132.68, 127.97 (d, *J* = 1.6 Hz), 121.02, 119.74 (d, *J* = 3.6 Hz), 115.02, 114.84, 114.62, 113.29 (d, *J* = 23.5 Hz), 107.81, 56.99, 56.18.

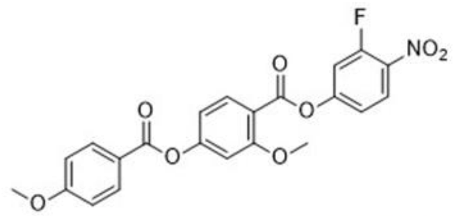

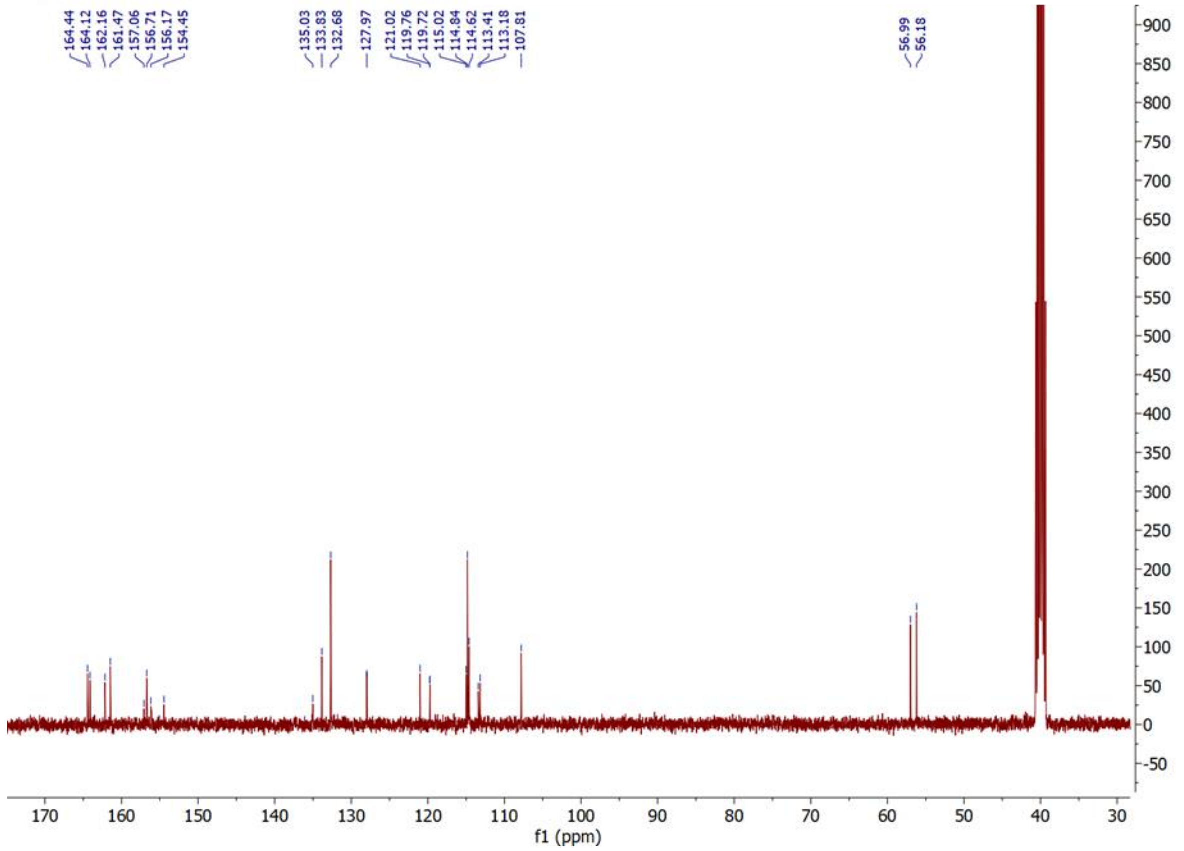

M/Z: $[M+Na]^+$ Calculated mass for $C_{22}H_{16}NO_8FNa$: 464.0758. Found: 464.0777. Difference: 4.1 ppm.

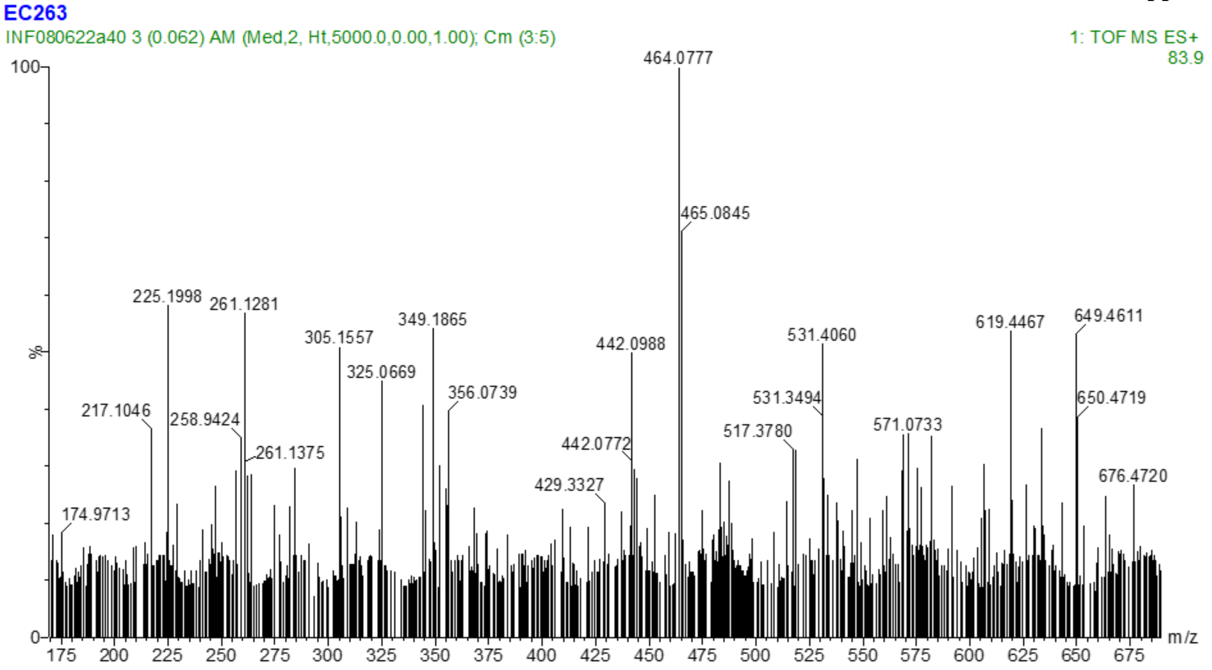

(3-Fluoro-4-nitrophenyl) 2-ethoxy-4-(4-methoxybenzoyl)oxybenzoate (NT3F.2)

Yield: 0.156 g, 32%. RF: 0.63 (97:3 DCM:EtOAc)

T_{CrI} 179 °C $T_{N_F I}$ (118 °C).

IR cm^{-1}: 3062 (Aryl -H), 2995 ($-CH_2$), 2848, 1740 (COOR), 1714, 1601 (Aryl C=C), 1524 (NO_2), 1484, 1433, 1358, 1290, 1255 (C-O-C), 1229 (C-F), 1173, 1143, 1043, 1024, 883, 877, 757, 684, 612, 530, 507, 420.

^{1}H NMR (400 MHz, DMSO) δ ppm: 8.31 (t, J = 8.9 Hz, 1H, Ar), 8.11 (d, J = 8.6 Hz, 2H, Ar), 8.07 (d, J = 8.6 Hz, 1H, Ar), 7.71 (dd, J = 12.0, 2.4 Hz, 1H, Ar), 7.42 (dt, J = 9.1, 1.2 Hz, 1H, Ar), 7.24 (d, J = 2.1 Hz, 1H, Ar), 7.15 (d, J = 8.5 Hz, 2H, Ar), 7.05 (dd, J = 8.6, 2.1 Hz, 1H, Ar), 4.17 (q, J = 6.9 Hz, 2H, -O$\underline{CH_2}CH_3$), 3.89 (s, 3H, -O$\underline{CH_3}$), 1.35 (t, J = 7.0 Hz, 3H, -O$CH_2\underline{CH_3}$).

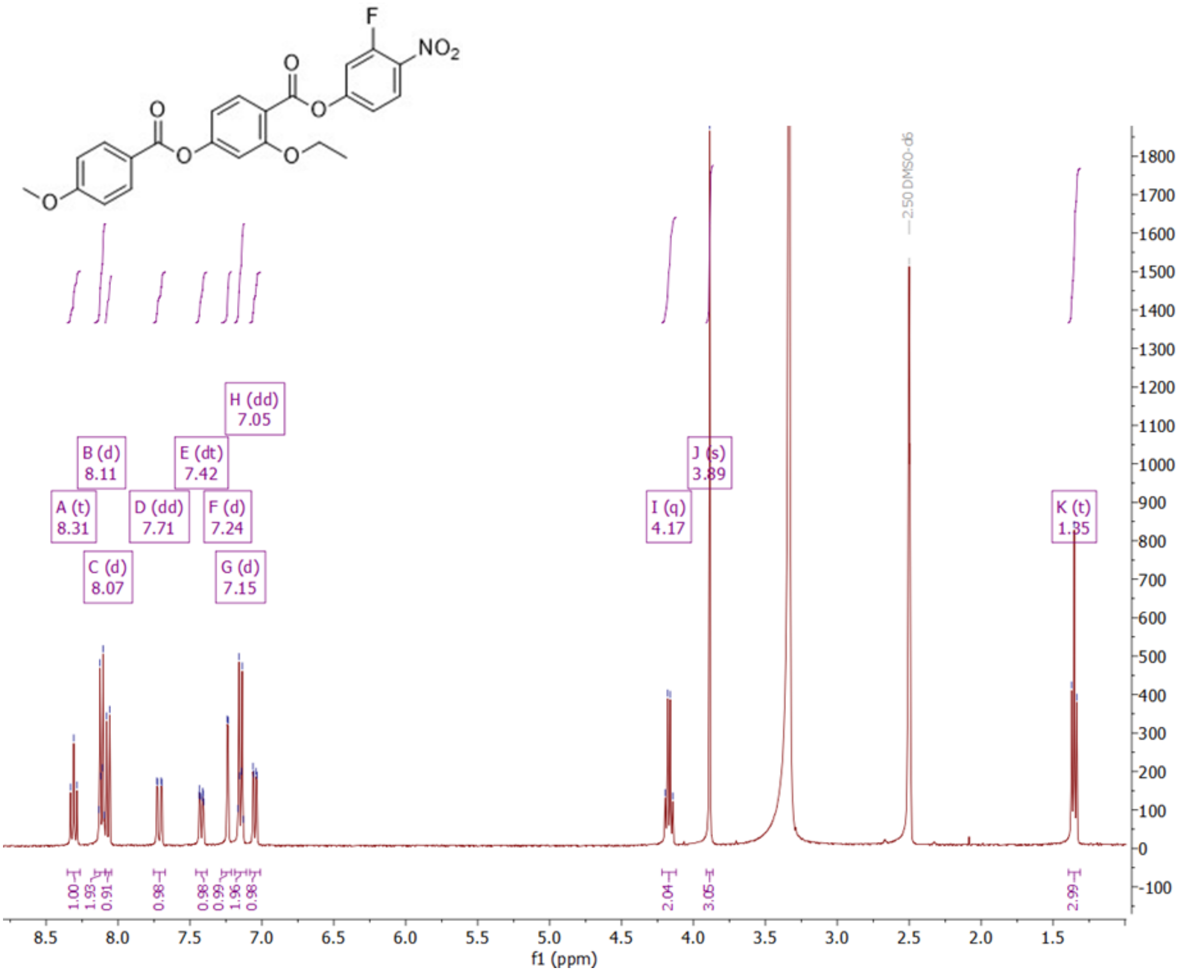

^{13}C NMR (101 MHz, DMSO) δ ppm: 164.00, 163.73, 161.91, 160.25, 156.16, 155.79 (d, *J* = 17.8 Hz), 154.05 (d, *J* = 264.6 Hz), 134.61 (d, *J* = 6.9 Hz), 133.33, 132.28, 127.61 (d, *J* = 1.4 Hz), 120.60, 119.22 (d, *J* = 3.6 Hz), 114.80, 114.41, 114.13, 112.81 (d, *J* = 23.7 Hz), 108.16, 64.80, 55.76, 14.44.

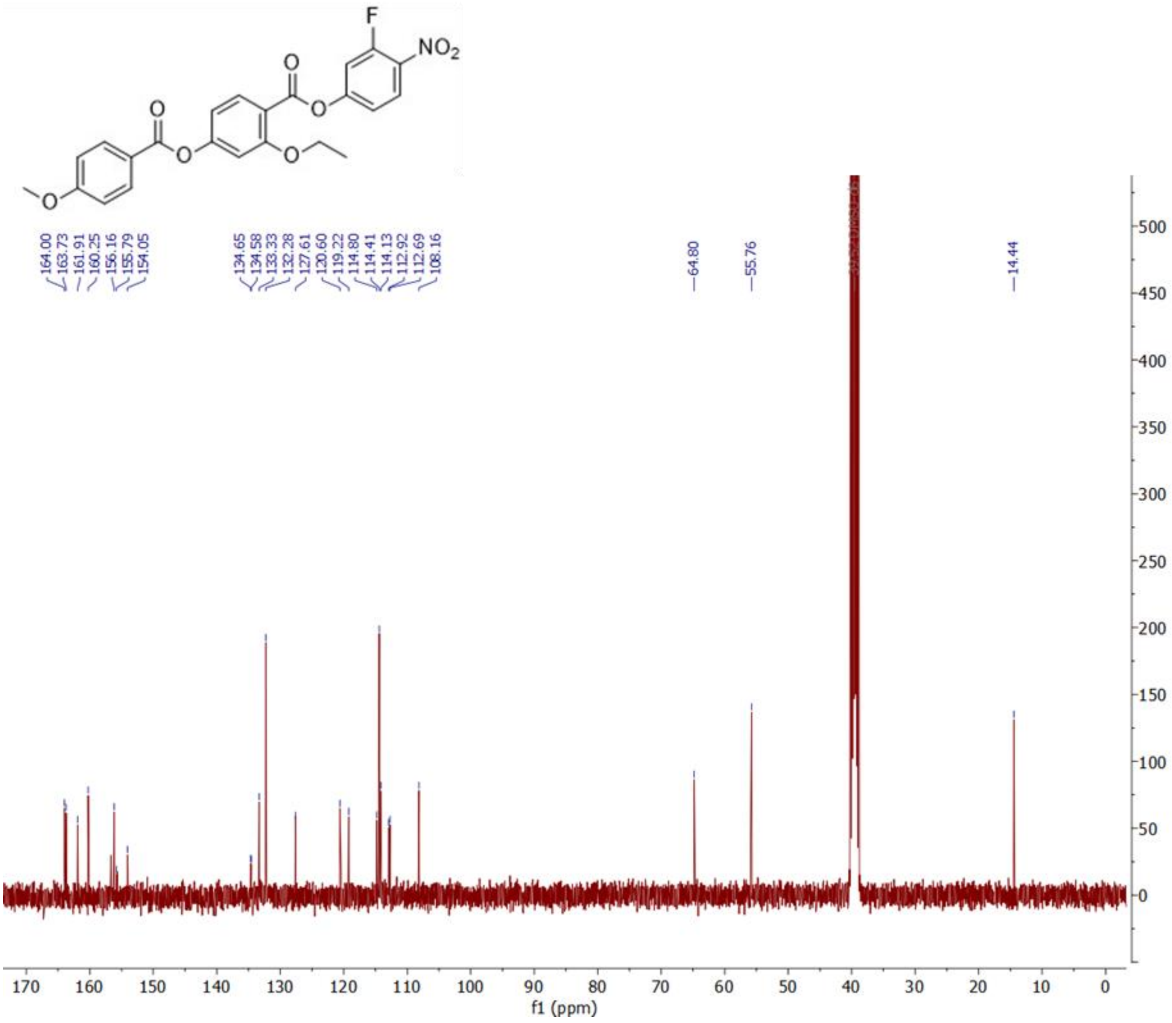

M/Z: [M+Na]$^{+}$ Calculated mass for $C_{23}H_{18}NO_8FNa$: 478.0914. Found: 478.0938. Difference: 5 ppm.

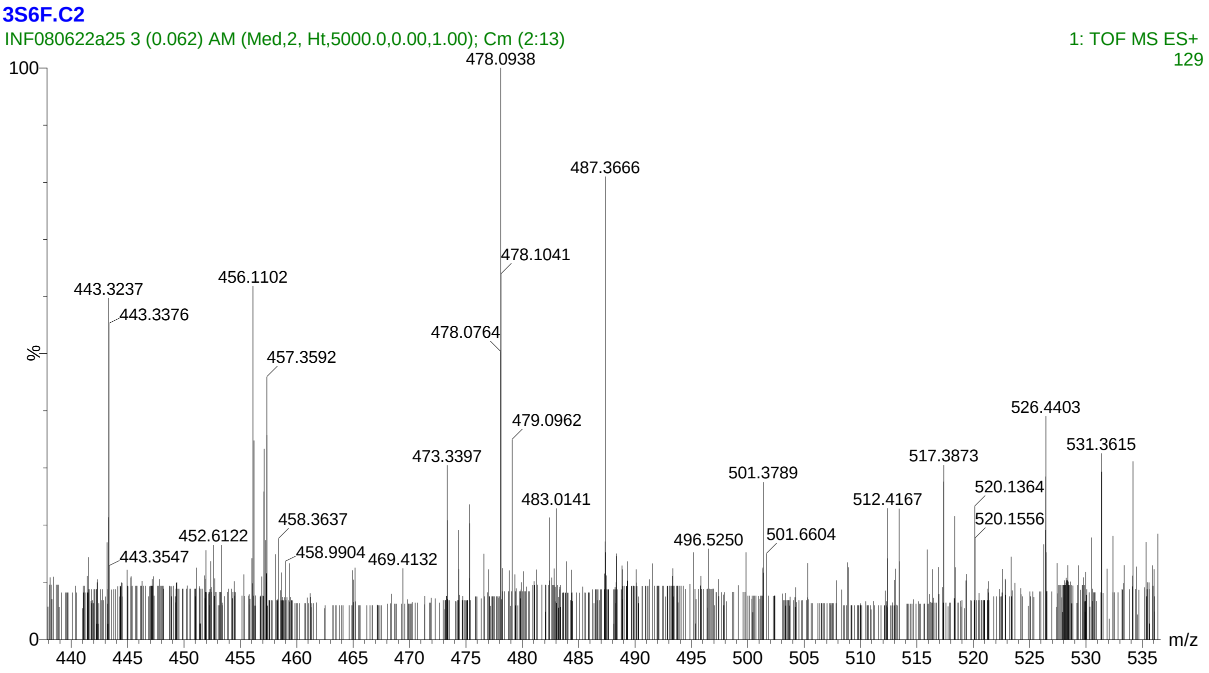

(3-Fluoro-4-nitrophenyl) 2-propoxy-4-(4-methoxybenzoyl)oxybenzoate (NT3F.3)
Yield: 0.116 g, 44%. RF: 0.71 (97:3 DCM:EtOAc)
T_{CrI} 135 °C T_{N_FI} (92 °C).

IR cm^{-1}: 3063 (Aryl -H), 2982 ($-CH_2$), 2848, 1738 (COOR), 1716, 1602 (Aryl C=C), 1525 (NO_2), 1428, 1356, 1287, 1254 (C-O-C), 1225 (C-F), 1168, 1114, 1040, 1018, 880, 841, 760, 685, 612, 531, 412.
^{1}H NMR (400 MHz, DMSO) δ ppm: 8.31 (t, J = 8.9 Hz, 1H, Ar), 8.11 (d, J = 8.9 Hz, 2H, Ar), 8.07 (d, J = 8.6 Hz, 1H, Ar), 7.71 (dd, J = 12.0, 2.4 Hz, 1H, Ar), 7.41 (dt, J = 9.0, 1.2 Hz, 1H, Ar), 7.24 (d, J = 2.1 Hz, 1H, Ar), 7.15 (d, J = 9.2 Hz, 2H, Ar), 7.05 (dd, J = 8.5, 2.1 Hz, 1H, Ar), 4.07 (t, J = 6.3 Hz, 2H, -O$\underline{CH_2}$$CH_2CH_3$), 3.89 (s, 3H, -O$\underline{CH_3}$), 1.75 (h, J = 7.0 Hz, 2H, -$OCH_2$$\underline{CH_2}$$CH_3$), 0.99 (t, J = 7.4 Hz, 3H, -$OCH_2CH_2$$\underline{CH_3}$).

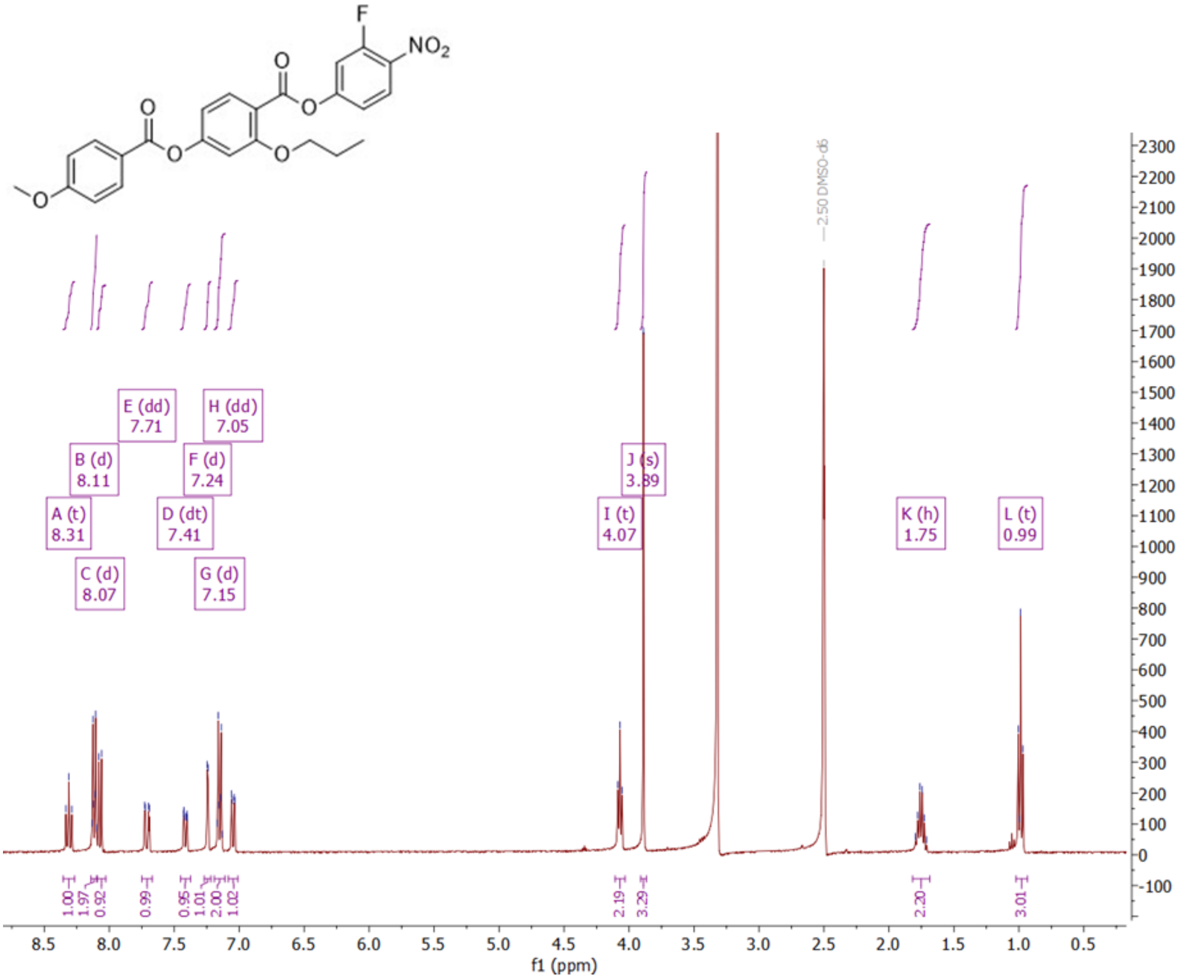

^{13}C NMR (101 MHz, DMSO) δ ppm: 163.98, 163.70, 161.97, 160.40, 155.78 (d, J = 11.0 Hz), 154.02 (d, J = 262.7 Hz), 134.60 (d, J = 7.3 Hz), 133.37, 132.24, 127.60 (d, J = 2.0 Hz), 120.59, 119.18 (d, J = 3.8 Hz), 114.68, 114.38, 114.05, 112.71 (d, J = 23.7 Hz), 108.04, 70.38, 55.73, 21.91, 10.40.

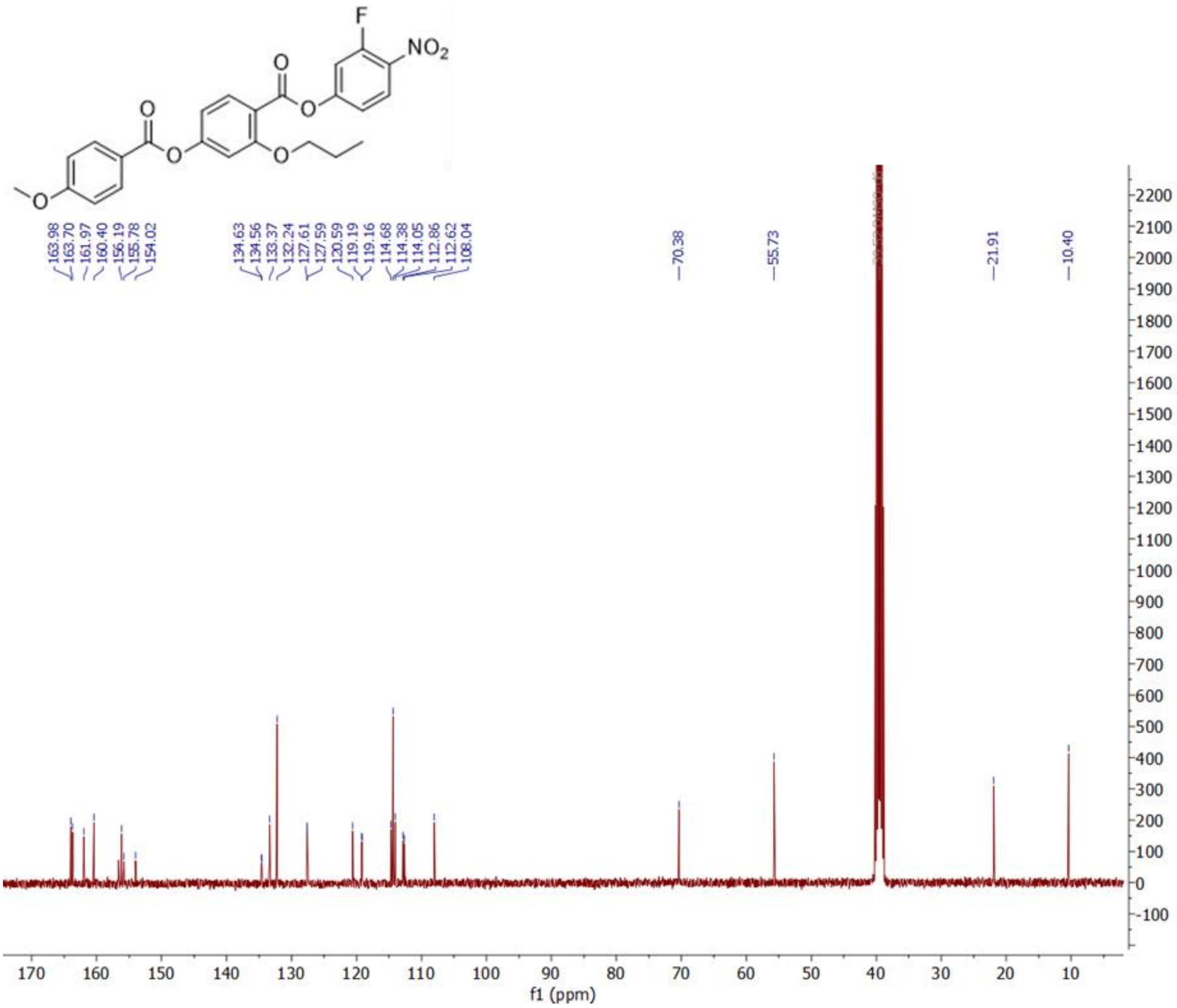

M/Z: [M+Na]$^+$ Calculated mass for $C_{24}H_{20}NO_8FNa$: 492.1071. Found: 492.1084. Difference: 2.6 ppm.

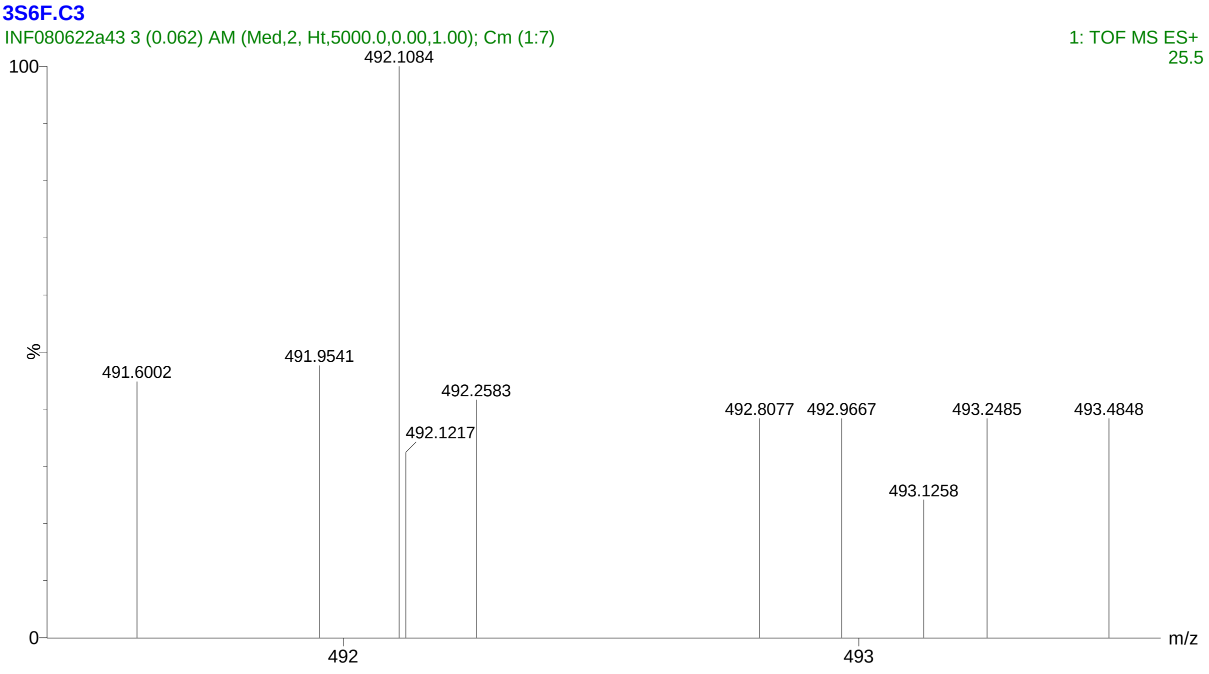

(3-Fluoro-4-nitrophenyl) 2-butoxy-4-(4-methoxybenzoyl)oxybenzoate (NT3F.4)
Yield: 0.041 g, 22%. RF: 0.65 (97:3 DCM:EtOAc)
T_{CrI} 124 °C T_{N_FI} (79 °C).
IR cm^{-1}: 3093 (Aryl -H), 2960 ($-CH_2$), 2874, 1736 (COOR), 1603 (Aryl C=C), 1531, 1510 (NO_2), 1348, 1254 (C-O-C), 1213 (C-F), 1158, 1127, 1004, 842, 759, 687, 604, 509, 408.
^{1}H NMR (400 MHz, DMSO) δ ppm: 8.31 (t, J = 8.9 Hz, 1H, Ar), 8.11 (d, J = 8.6 Hz, 2H, Ar), 8.07 (d, J = 8.6 Hz, 1H, Ar), 7.70 (dd, J = 12.0, 2.4 Hz, 1H, Ar), 7.41 (ddd, J = 9.0, 2.5, 1.2 Hz, 1H, Ar), 7.25 (d, J = 2.1 Hz, 1H, Ar), 7.14 (d, J = 8.4 Hz, 2H, Ar), 7.04 (dd, J = 8.6, 2.1 Hz, 1H, Ar), 4.10 (t, J = 6.3 Hz, 2H, $-O\underline{CH_2}CH_2CH_2CH_3$), 3.89 (s, 3H, $-O\underline{CH_3}$), 1.72 (p, J = 6.3 Hz, 2H, $-OCH_2\underline{CH_2}CH_2CH_3$), 1.46 (h, J = 7.3 Hz, 2H, $-OCH_2CH_2\underline{CH_2}CH_3$), 0.90 (t, J = 7.4 Hz, 3H, $-OCH_2CH_2CH_2\underline{CH_3}$).

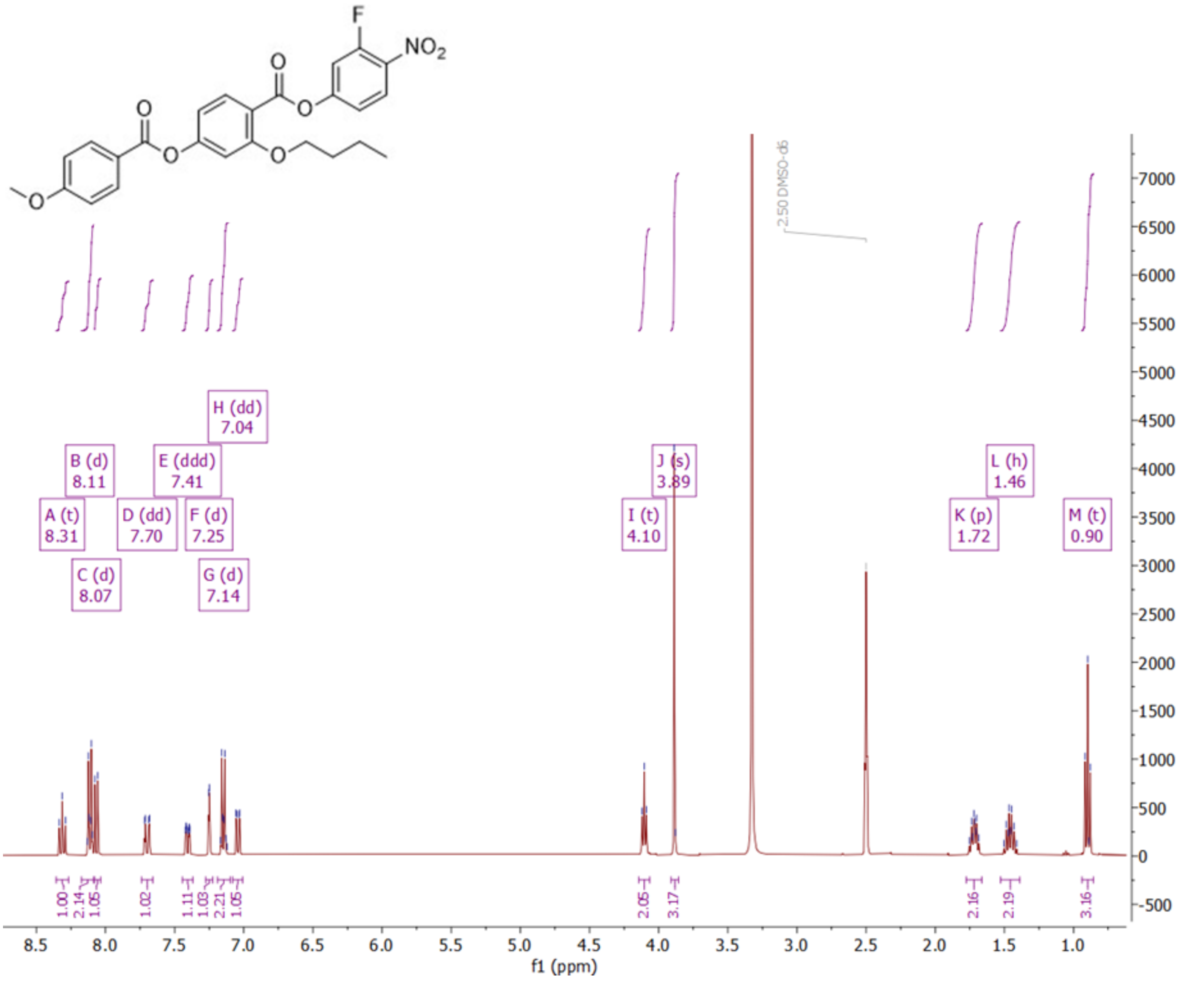

^{13}C NMR (101 MHz, DMSO) δ ppm: 163.95, 163.66, 161.90, 160.39, 156.16, 155.77 (d, J = 12.5 Hz), 153.99 (d, J = 262.7 Hz), 134.53 (d, J = 6.9 Hz), 133.31, 132.21, 127.56, 120.57, 119.16 (d, J = 3.8 Hz), 114.65, 114.35, 114.02, 112.71 (d, J = 23.7 Hz), 108.02, 68.57, 55.71, 30.49, 18.59, 13.57.

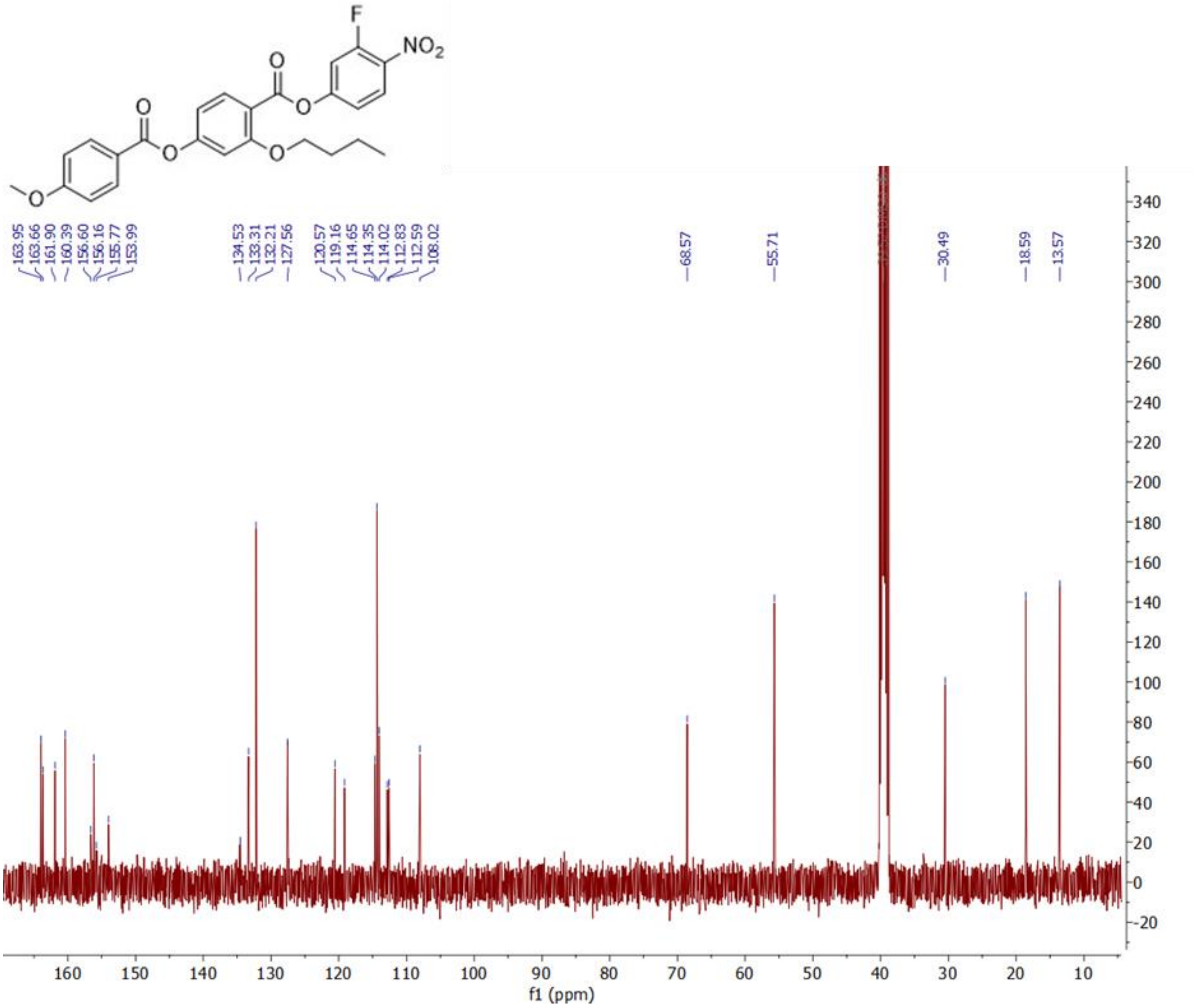

M/Z: $[M+H]^+$ Calculated mass for $C_{25}H_{23}NO_8F$: 484.1418. Found: 484.1408. Difference: 2.1 ppm. $[2M+Na]^+$ Calculated mass for $C_{50}H_{44}N_2O_{16}F_2Na$: 989.2557. Found: 989.2590. Difference: 3.3 ppm.

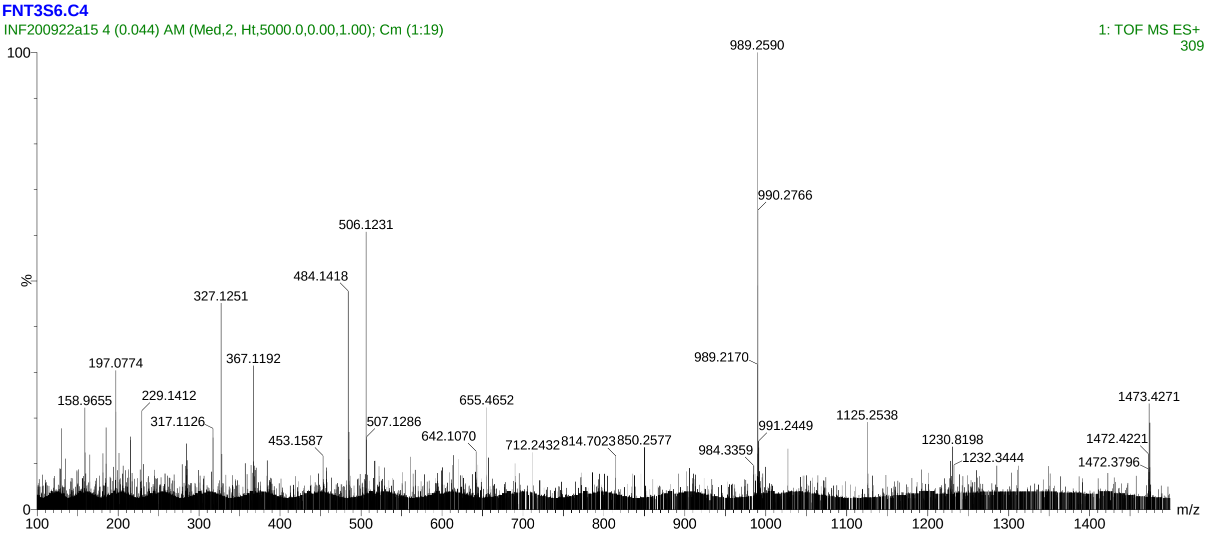

(3-Fluoro-4-nitrophenyl) 2-pentoxy-4-(4-methoxybenzoyl)oxybenzoate (NT3F.5)

Yield: 0.122 g, 44%. RF: 0.70 (97:3 DCM:EtOAc)

T_{CrI} 104 °C T_{N_FI} (68 °C).

IR cm^{-1}: 3085 (Aryl -H), 2941 (-CH_2), 2873, 1759, 1735 (COOR), 1602 (Aryl C=C), 1511 (NO_2), 1428, 1350, 1252 (C-O-C), 1213 (C-F), 1147, 1122, 1003, 841, 759, 682, 607, 567, 509, 408.

^{1}H NMR (400 MHz, DMSO) δ ppm: 8.31 (t, J = 8.8 Hz, 1H, Ar), 8.11 (d, J = 8.6 Hz, 2H, Ar), 8.06 (d, J = 7.6 Hz, 1H, Ar), 7.70 (dd, J = 12.0, 2.4 Hz, 1H, Ar), 7.41 (dd, J = 9.0, 2.5 Hz, 1H, Ar), 7.25 (d, J = 2.1 Hz, 1H, Ar), 7.15 (d, J = 8.6 Hz, 2H, Ar), 7.04 (dd, J = 8.5, 1.7 Hz, 1H, Ar), 4.10 (t, J = 6.2 Hz, 2H, -O$\underline{CH_2}CH_2CH_2CH_2CH_3$), 3.89 (s, 3H, -O$\underline{CH_3}$), 1.74 (p, J = 6.5 Hz, 2H, -$OCH_2\underline{CH_2}CH_2CH_2CH_3$), 1.36 (m, 4H, -$OCH_2CH_2\underline{CH_2CH_2}CH_3$), 0.83 (t, J = 7.2 Hz, 3H, -$OCH_2CH_2CH_2CH_2\underline{CH_3}$).

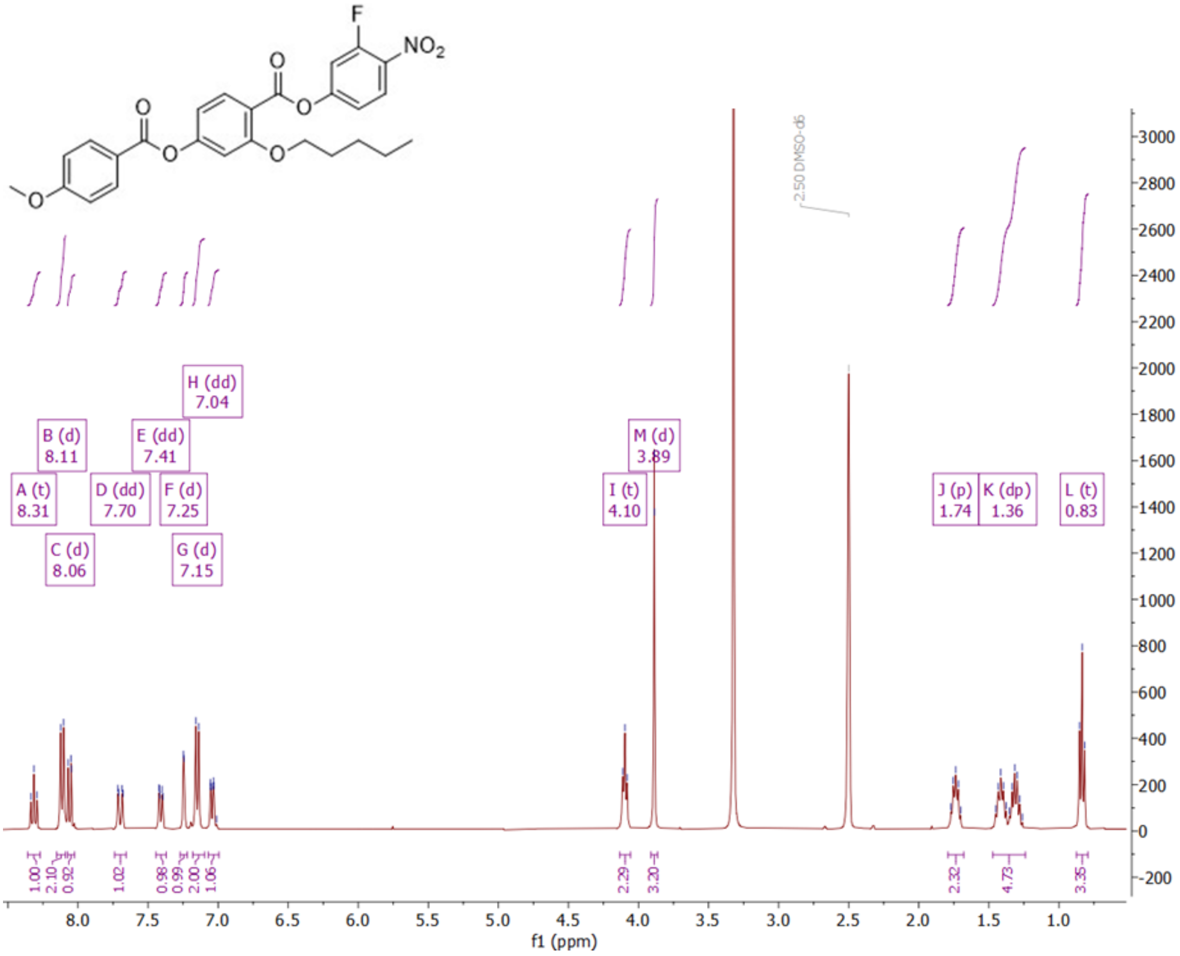

^{13}C NMR (101 MHz, DMSO) δ ppm: 163.97, 163.70, 162.03, 160.38, 156.18, 155.74 (d, J = 11.1 Hz), 154.01 (d, J = 260.8 Hz), 134.58 (d, J = 7.4 Hz), 133.34, 132.23, 127.59, 120.59, 119.14 (d, J = 3.6 Hz), 114.71, 114.37, 114.05, 112.69 (d, J = 23.7 Hz), 108.03, 68.90, 55.73, 28.15, 27.59, 21.76, 13.88.

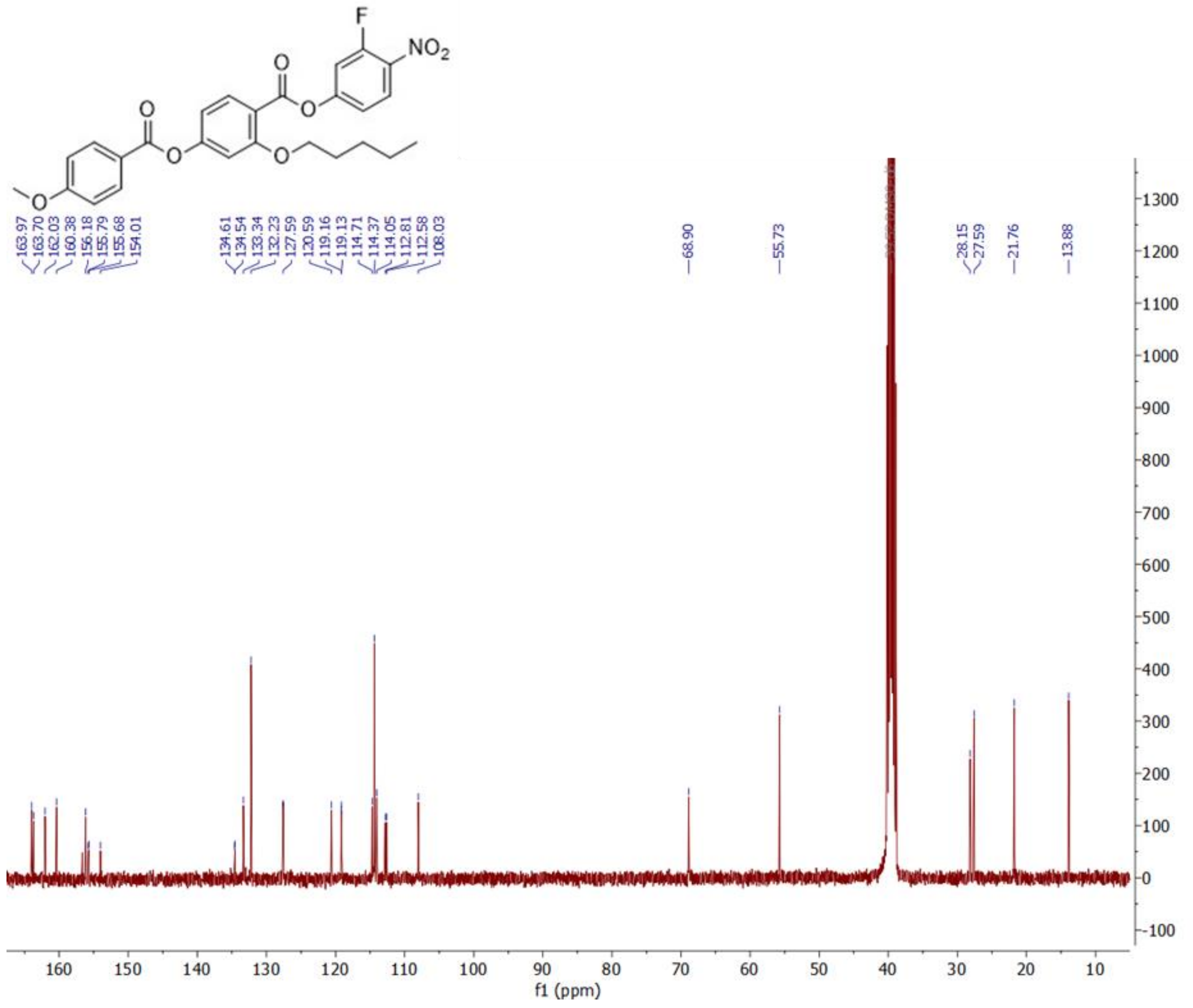

M/Z: $[2M+Na]^+$ Calculated mass for $C_{52}H_{48}N_2O_{16}F_2Na$: 1017.2870. Found: 1017.2914. Difference: 4.3 ppm.

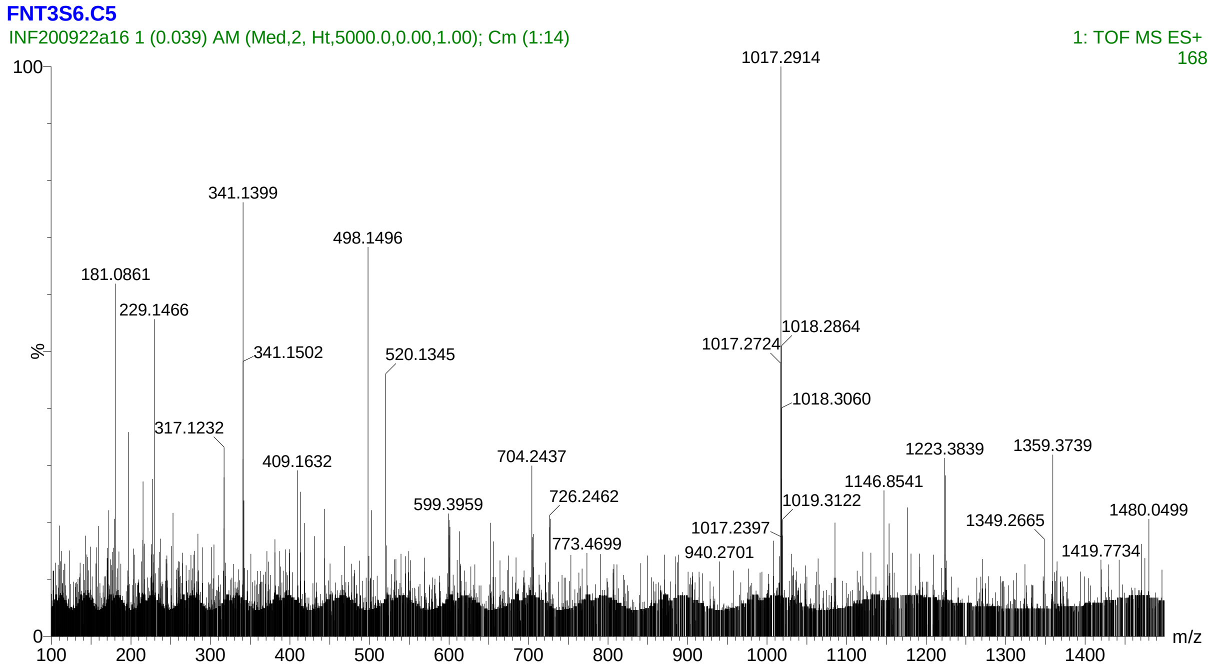

(3-Fluoro-4-nitrophenyl) 2-hexoxy-4-(4-methoxybenzoyl)oxybenzoate (NT3F.6)
Yield: 0.159 g, 59%. RF: 0.23 (DCM)
T_{CrI} 80 °C T_{N_FI} (58 °C).
IR cm^{-1}: 3095 (Aryl -H), 2942 (-CH_2), 2874, 1761, 1731 (COOR), 1603 (Aryl C=C), 1511 (NO_2), 1428, 1352, 1251 (C-O-C), 1216 (C-F), 1167, 1122, 1003, 841, 760, 682, 608, 508, 408.
^{1}H NMR (400 MHz, DMSO) δ ppm: 8.32 (t, J = 8.9 Hz, 1H, Ar), 8.12 (d, J = 8.9 Hz, 2H, Ar), 8.06 (d, J = 8.6 Hz, 1H, Ar), 7.70 (dd, J = 11.9, 2.4 Hz, 1H, Ar), 7.41 (ddd, J = 9.1, 2.5, 1.2 Hz, 1H, Ar), 7.25 (d, J = 2.1 Hz, 1H, Ar), 7.16 (d, J = 8.9 Hz, 2H, Ar), 7.05 (dd, J = 8.6, 2.1 Hz, 1H, Ar), 4.10 (t, J = 6.3 Hz, 2H, -O$\underline{CH_2}CH_2CH_2CH_2CH_2CH_3$), 3.90 (s, 3H, -O$\underline{CH_3}$), 1.73 (p, J = 7.1 Hz, 2H, -$OCH_2\underline{CH_2}CH_2CH_2CH_2CH_3$), 1.49 – 1.38 (m, 2H, -$OCH_2CH_2\underline{CH_2}CH_2CH_2CH_3$), 1.33 – 1.17 (m, 4H, -$OCH_2CH_2CH_2\underline{CH_2CH_2}CH_3$), 0.86 – 0.78 (m, 3H, -$OCH_2CH_2CH_2CH_2CH_2\underline{CH_3}$).

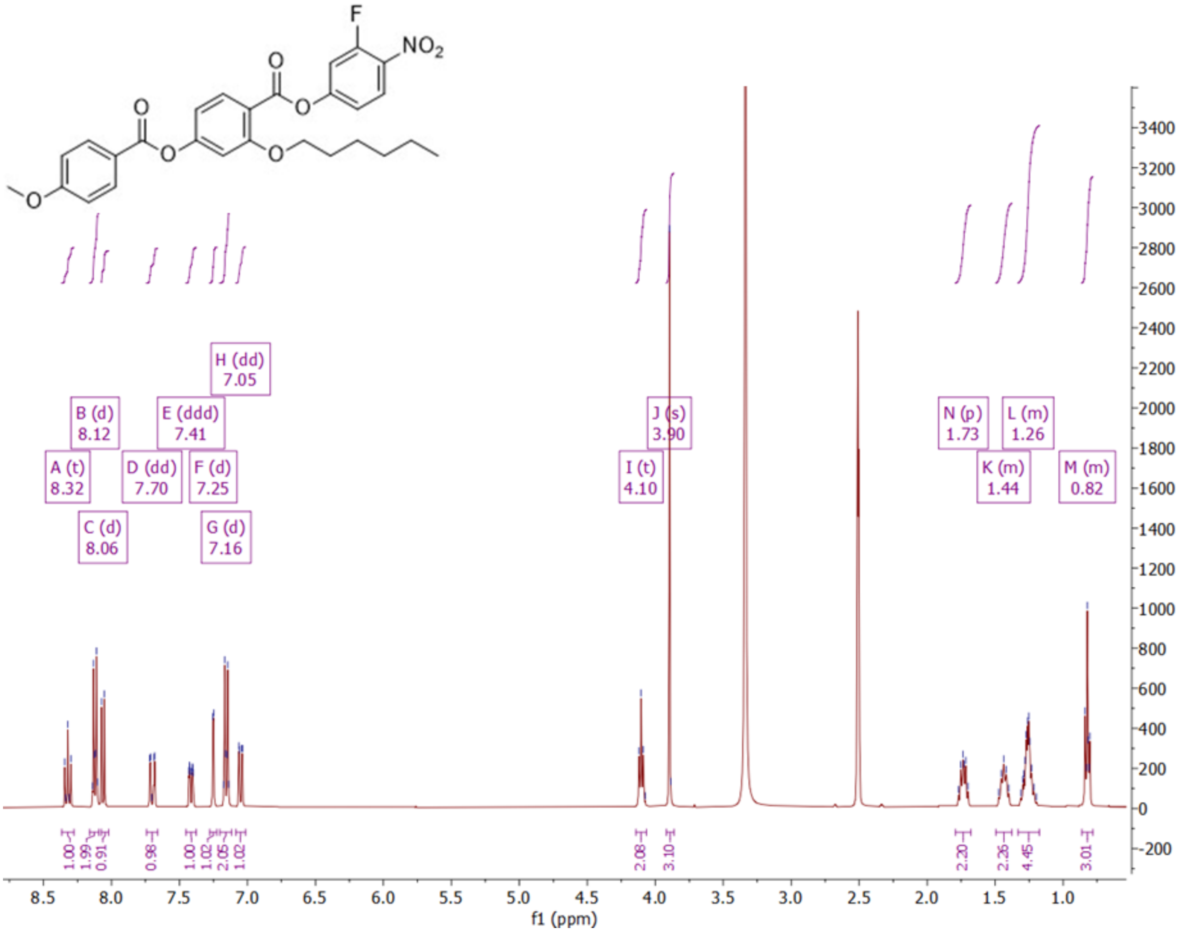

^{13}C NMR (101 MHz, DMSO) δ ppm: 163.95, 163.67, 162.05, 160.33, 156.57 (d, J = 264.3 Hz), 156.15, 155.76 (d, J = 11.6 Hz), 134.56 (d, J = 7.2 Hz), 133.30, 132.21, 127.57 (d, J = 2.0 Hz), 120.57, 119.14 (d, J = 4.1 Hz), 114.72, 114.36, 114.04, 112.54 (d, J = 25.2 Hz), 108.02, 68.89, 55.71, 30.84, 28.41, 25.05, 22.04, 13.81.

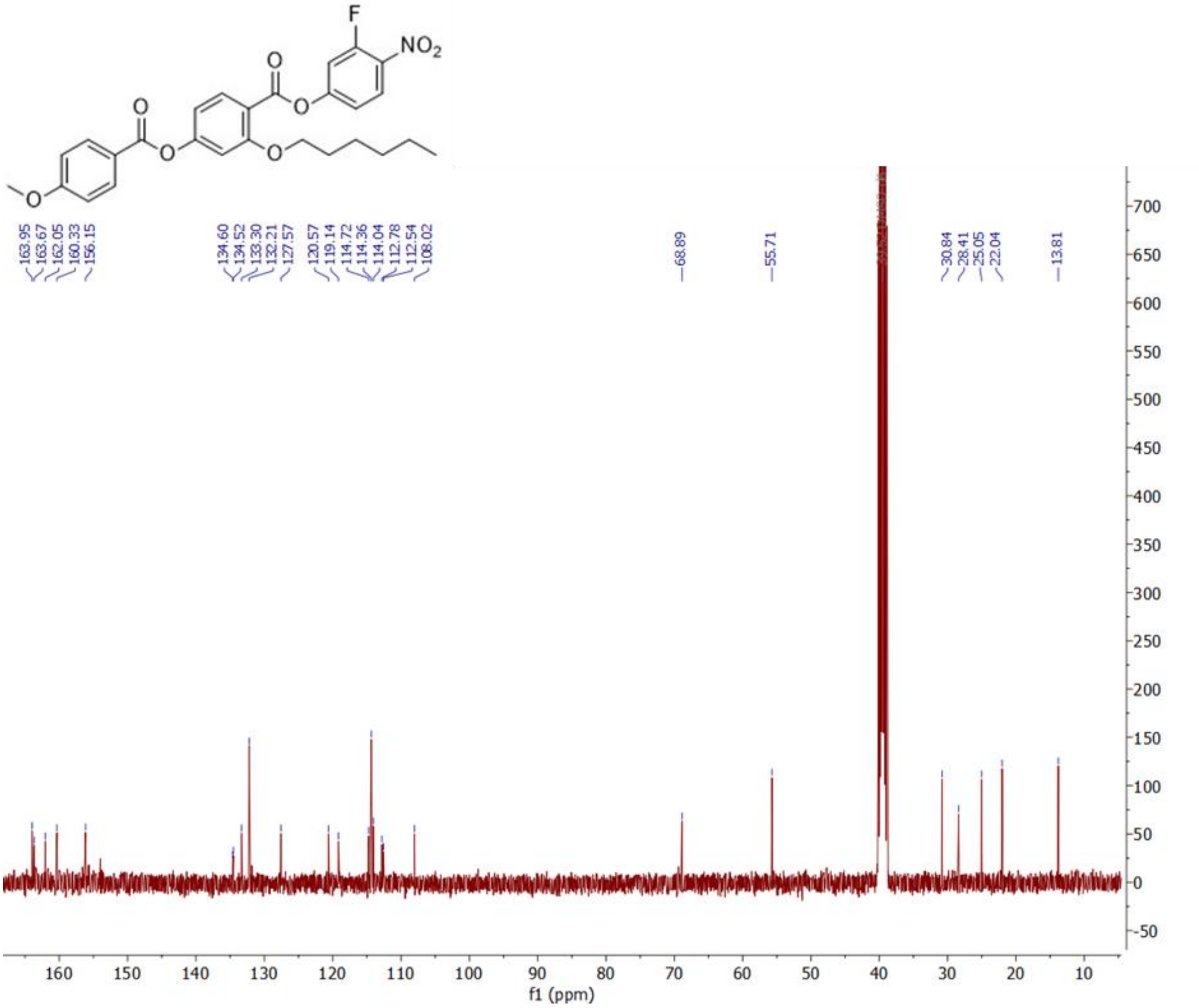

M/Z: $[M+Na]^+$ Calculated mass for $C_{27}H_{26}NO_8FNa$: 534.1540. Found: 534.1525. Difference: 2.8 ppm. $[2M+Na]^+$ Calculated mass for $C_{54}H_{52}NO_{16}F_2Na$: 1045.3183. Found: 1045.3179. Difference: 0.4 ppm.

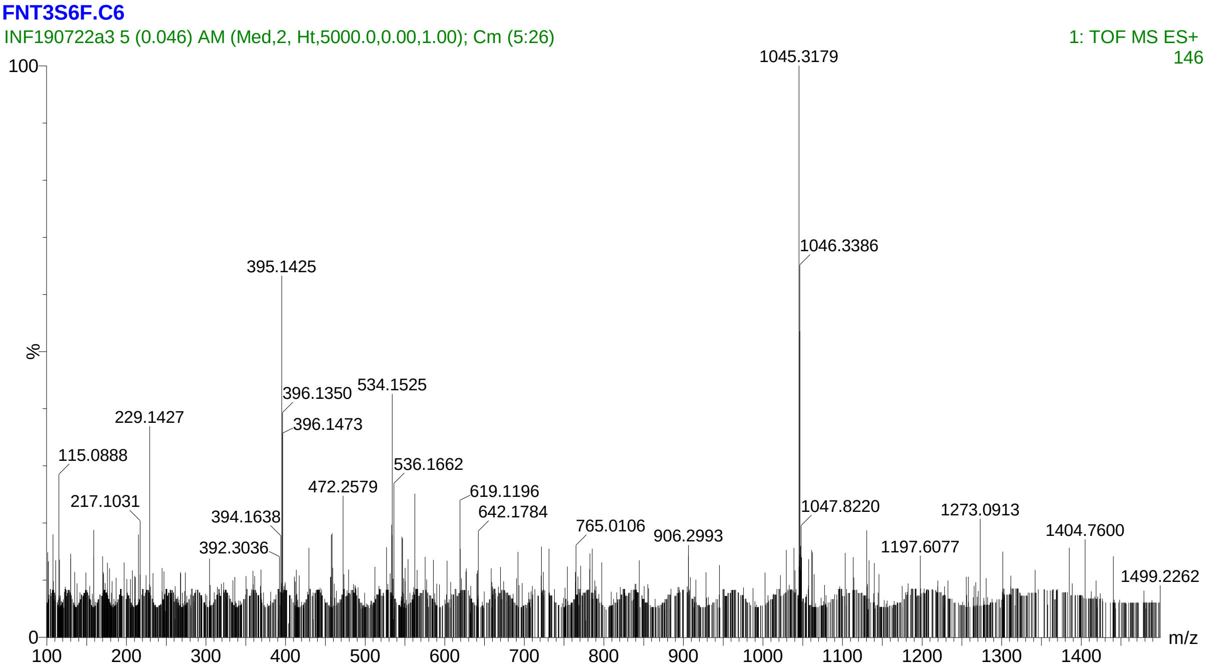

(3-Fluoro-4-nitrophenyl) 2-heptoxy-4-(4-methoxybenzoyl)oxybenzoate (NT3F.7)
Yield: 0.161 g, 40%. RF: 0.52 (DCM)
T_{CrI} 73 °C T_{N_FI} (55 °C).
IR cm^{-1}: 3083 (Aryl -H), 2919 (-CH_2), 2855, 1761, 1730 (COOR), 1602 (Aryl C=C), 1512 (NO_2), 1427, 1353, 1254 (C-O-C), 1216 (C-F), 1168, 1147, 1122, 1004, 877, 841, 760, 681, 608, 508, 409.
^{1}H NMR (400 MHz, DMSO) δ ppm: 8.32 (t, J = 8.8 Hz, 1H, Ar), 8.12 (d, J = 8.5 Hz, 2H, Ar), 8.06 (d, J = 8.6 Hz, 1H, Ar), 7.70 (dd, J = 12.0, 2.4 Hz, 1H, Ar), 7.42 (ddd, J = 9.1, 2.4, 1.2 Hz, 1H, Ar), 7.25 (d, J = 2.1 Hz, 1H, Ar), 7.16 (d, J = 8.8 Hz, 2H, Ar), 7.05 (dd, J = 8.6, 2.1 Hz, 1H, Ar), 4.10 (t, J = 6.2 Hz, 2H, -$O\underline{CH_2}CH_2CH_2CH_2CH_2CH_2CH_3$), 3.89 (s, 3H, -$O\underline{CH_3}$), 1.73 (p, J = 6.4 Hz, 2H, -$OCH_2\underline{CH_2}CH_2CH_2CH_2CH_2CH_3$), 1.42 (p, J = 7.2 Hz, 2H, -$OCH_2CH_2\underline{CH_2}CH_2CH_2CH_2CH_3$), 1.34 – 1.15 (m, 6H, -$OCH_2CH_2CH_2\underline{CH_2}\underline{CH_2}\underline{CH_2}CH_3$), 0.81 (t, J = 6.8 Hz, 3H, -$OCH_2CH_2CH_2CH_2CH_2CH_2\underline{CH_3}$).

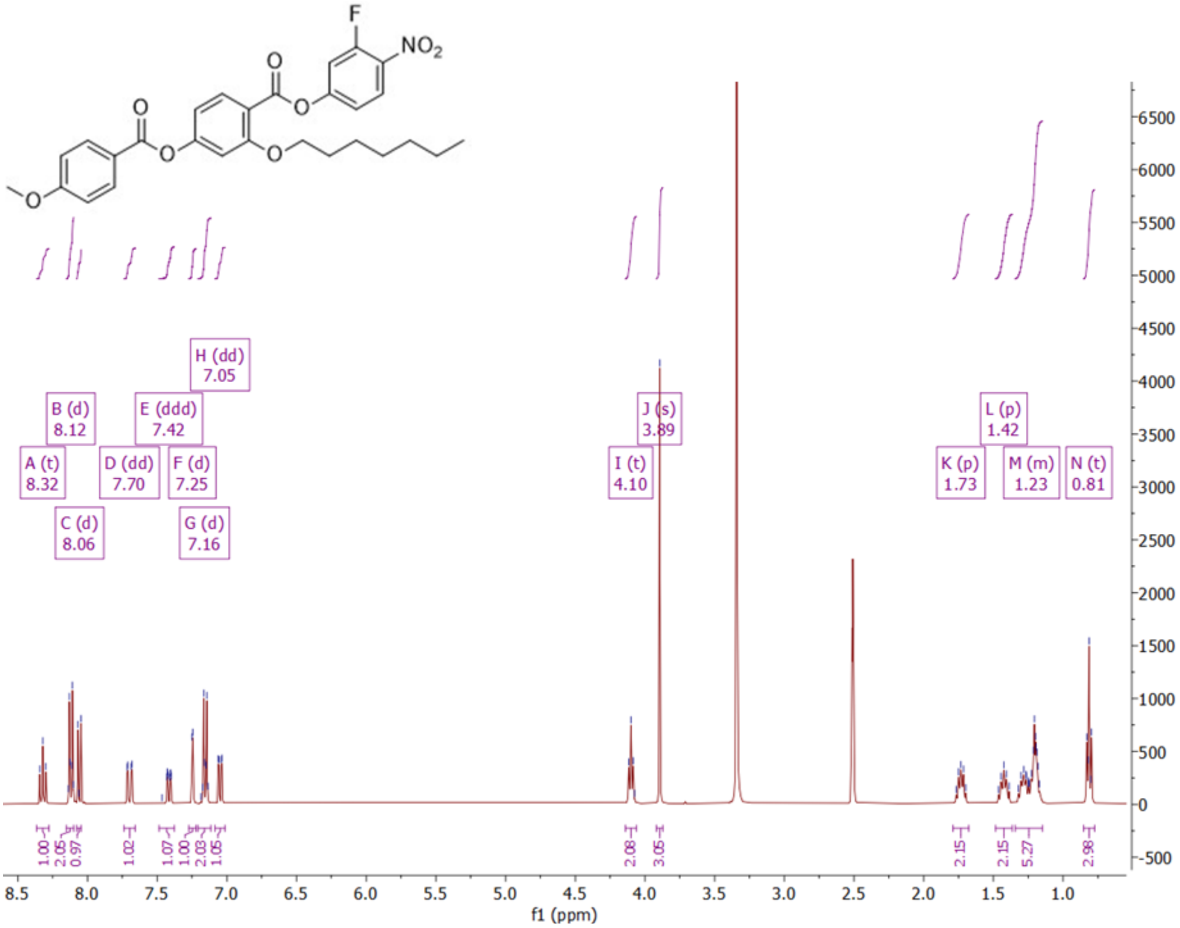

^{13}C NMR (101 MHz, DMSO) δ ppm: 163.94, 163.66, 162.10, 160.31, 156.14, 155.77 (d, J = 82.3 Hz), 153.98 (d, J = 263.9 Hz), 134.57 (d, J = 7.6 Hz), 133.30, 132.19, 127.53 (d, J = 1.7 Hz), 120.56, 119.08 (d, J = 3.4 Hz), 114.72, 114.34, 114.03, 112.75 (d, J = 23.6 Hz), 108.01, 68.87, 55.70, 31.22, 28.46, 28.320, 25.36, 21.98, 13.88.

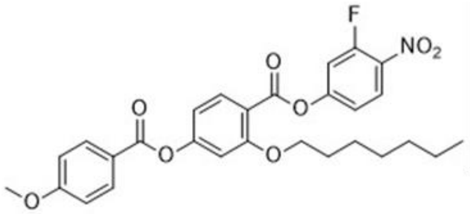

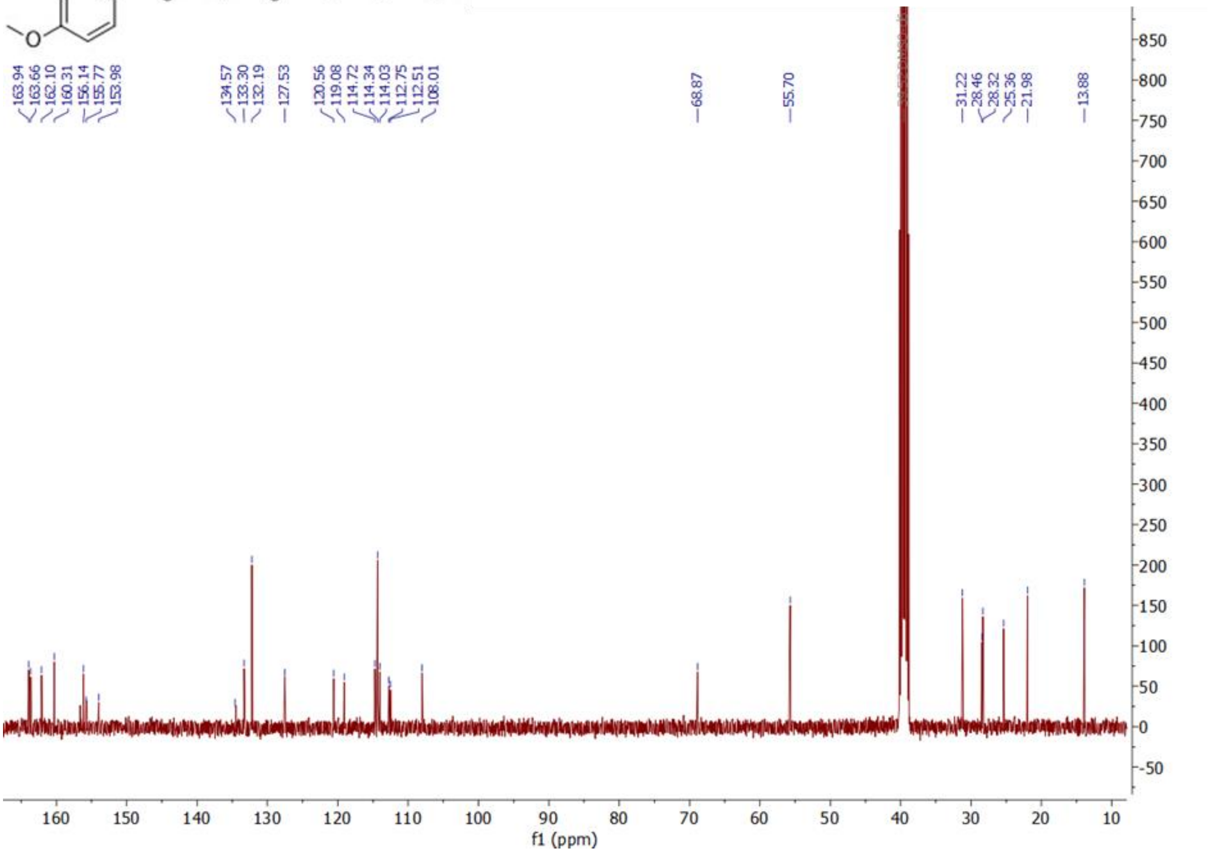

M/Z: $[M+H]^+$ Calculated mass for $C_{28}H_{29}NO_8F$: 526.1877. Found: 526.1885. Difference: 1.5 ppm.

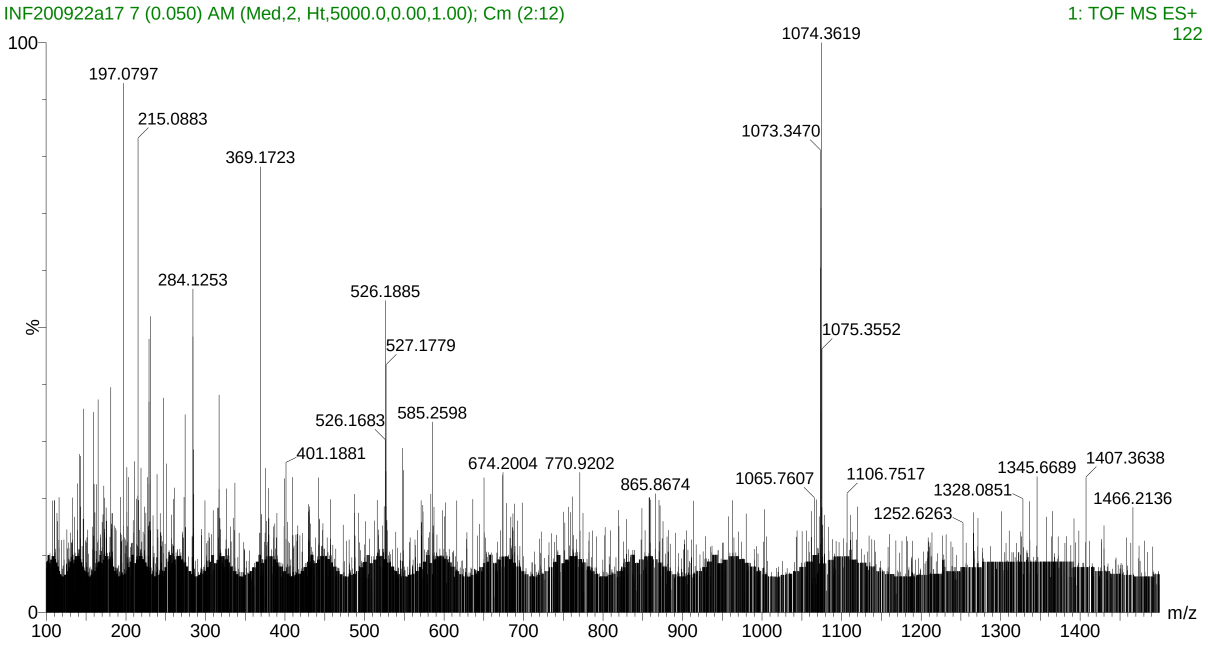

Additional Experimental Results

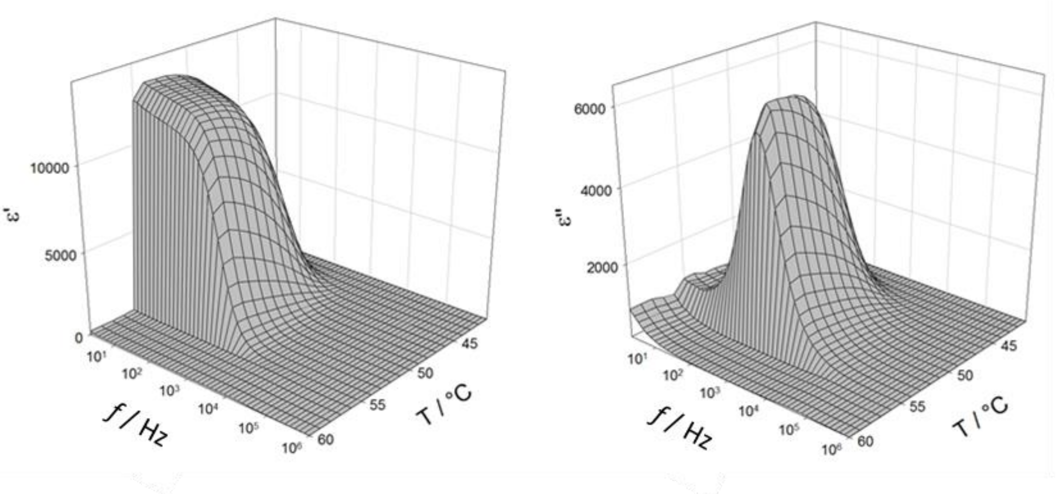

Figure SI1: (a) Real and (b) imaginary parts of the complex dielectric permittivity measured vs temperature and frequency for NT3F.7, in a 9.7-μm-thick cell with ITO electrodes and no alignment layer.

Table SI7: Calculated molecular dipole moments for the lowest energy conformations of the NT3.*m* and NT3F.*m* series. Calculated at the B3LYP/6-31(d) level of theory.

m	NT3.*m* μ / D	NT3F.*m* μ / D
1	11.59	12.64
2	11.60	12.64
3	11.63	12.68
4	11.69	12.79
5	11.72	12.82
6	11.74	12.84
7	11.72	12.86

References

1. J. Li, H. Nishikawa, J. Kougo, J. Zhou, S. Dai, W. Tang, X. Zhao, Y. Hisai, M. Huang, S. Aya. *Sci. Adv.* (2021), **7**, eabf5047.
